# Discovery of Dimer-Dependent Aminoacrylamide Molecular Glues for 14–3–3 Protein–Protein Interactions

**DOI:** 10.1021/acsmedchemlett.5c00686

**Published:** 2026-01-12

**Authors:** Paulo Pitasse-Santos, Marta Falcicchio, Rajdeep Sahota, Hadeeqa G. Raza, Aneika C. Leney, Richard H. Cowan, Gareth Hall, Richard G. Doveston

**Affiliations:** † School of Chemistry and Institute of Structural and Chemical Biology,University of Leicester, University Road, Leicester LE1 7RH, U.K.; ‡ School of Bioscience, 1724 University of Birmingham, Edgbaston, Birmingham, B15 2TT, U.K.; § School of Biological Sciences and Institute of Structural and Chemical Biology,University of Leicester, University Road, Leicester, LE1 7RH, U.K.

**Keywords:** Molecular glue, protein−protein interactions, 14−3−3 proteins, dimeric interface

## Abstract

Molecular glues (MGs) offer a promising strategy for stabilizing protein–protein interactions (PPIs), particularly within the 14–3–3 protein family, which regulates diverse cellular processes and is implicated in many disease pathways. This study reports on the discovery of an aminoacrylamide MG (**7**) for 14–3–3 PPIs. Structure–activity relationship analysis using a fluorescence polarization (FP) assay revealed that both a basic amine and acrylamide moiety are essential for activity. However, further investigation using FP, mass spectrometry, and a thermal shift assay revealed that **7** has a cysteine-independent mode of action, distinguishing it from other covalent 14–3–3 MGs. Furthermore, its activity is reliant on 14–3–3 dimerization suggesting that it targets the 14–3–3 dimer interface. Aminoacrylamide **7** differentially affected interactions with ERα, LRRK2, and AHA2, suggesting that 14–3–3 dimerization plays an important role in 14–3–3 client recognition. These findings further validate the 14–3–3 dimer interface as a novel MG target and underscore the complexities of 14–3–3 molecular recognition and small-molecule modulation.

## Graphical abstract

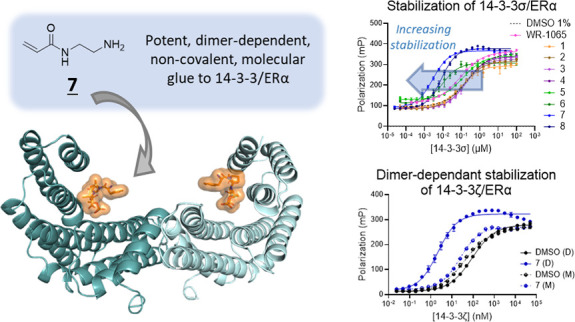

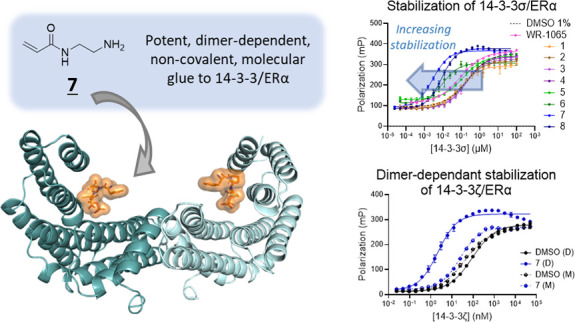

Molecular glues (MGs) that stabilize the interaction between two proteins provide an opportunity to expand the druggable proteome.^1,2^ MGs stabilize weak native interactions via binding to either a composite pocket at the interface of two interacting proteins (e.g., rapamycin)^3^ or at an allosteric site distal to the interface (e.g., taxol).^4,5^ In particular, MG degraders have recently emerged as effective pharmaceutical agents in a range of disease settings.^6^ These compounds stabilize the interaction of protein targets with E3 ligase effector proteins, thus promoting targeted protein degradation without the need for large bifunctional molecules (i.e., ProTaCs). Using MGs to harness other effector proteins such as kinases^7^ and phosphatases^8^ has enormous potential, but development lags behind that for MG degraders.

14–3–3 proteins are a family of hub proteins that interact with several hundred protein clients in a phosphorylation-dependent manner to modulate client cellular localization, signal transduction, transcription, and the cell cycle.^9,10^ Many 14–3–3 protein–protein interactions (PPIs) are critical for mediating cellular responses to diseases such as cancer^11^ and neurodegeneration.^12^ Therefore, they are an important class of effector protein whereby stabilization of their interactions with clients could be an effective therapeutic strategy.^13^

The 14–3–3 family comprises seven structurally homologous dimeric isoforms in humans: β, γ, ε, η, σ, τ, and ζ. They recognize their clients via disordered consensus motifs containing phosphorylated serine (pS) or threonine (pT) residues that bind in the highly conserved amphipathic binding groove on each 14–3–3 monomer.^14^ There are three main classes of consensus motifs: mode I [RSX­(pS/T)­XP], mode II [RX­(Y/F)­X­(PS/T)­XP], or mode III [(pS/T)­X-COOH].^15^ However, not all 14–3–3 clients contain a motif that falls within these classifications. In a number of cases, client binding requires two phosphorylated motifs which interact with the 14–3–3 dimer in a multivalent fashion^16^ or ‘rock’ between the two binding grooves.^17,18^ 14–3–3 dimerization is an important feature that facilitates molecular recognition,^19^ and modulation of the dimer interface using small molecules can influence client binding affinity.^20–23^

14–3–3 PPIs are amenable to stabilization by MGs, especially where clients contain a C-terminal mode III consensus motif.^24^ In these cases, the formation of a binary complex reveals a composite ligand binding pocket. This pocket can be targeted by MGs to form stabilized ternary complexes.^25^ A focal point for 14–3–3 MG development has been on the interaction with estrogen receptor alpha (ERα). 14–3–3 interacts with ERα via a mode III motif requiring phosphorylation of the penultimate T594 residue [(pT)­V-COOH].^26,27^ This interaction impedes ERα dimerization, DNA binding, and, thus, transcriptional activity, which is a key driver of breast cancer. Stabilization of the 14–3–3/ERα by the natural product fusicoccin A (FC-A, Figure 1A) enhances the negative regulatory effect of 14–3–3 on ERα leading to reduced breast cancer cell proliferation.^27,28^ Rational design approaches have subsequently led to the denovo development of more synthetically accessible reversible MGs for this interaction.^29,30^

**Figure 1:**
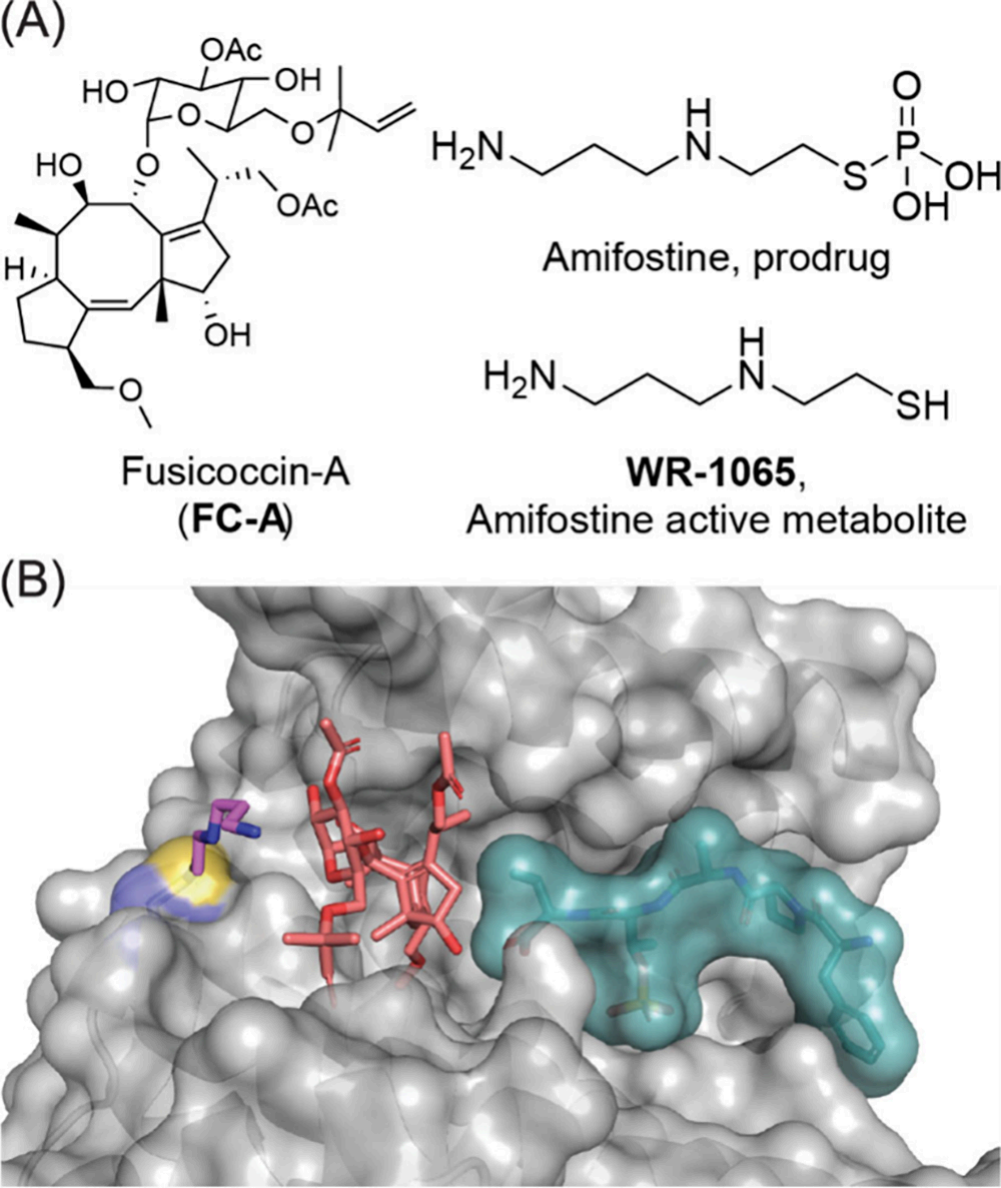
(A) Chemical structures of fusicoccin A (FC-A), amifostine, and its active metabolite WR-1065. (B) Crystal structure of 14–3–3σ (gray) covalently bound to WR-1065 (pink), in complex FC-A (red), and ERα phosphopeptide (cyan) (PDB 7NIZ).

Irreversible 14–3–3 MGs have emerged as a powerful modality for stabilizing 14–3–3 PPIs with isoform and client selectivity.^31^ The 14–3–3σ isoform contains a unique cysteine residue (C38) adjacent to the 14–3–3 binding groove and MG pocket which can be exploited both for ligand discovery^32^ and drug development.^33^ Among the first examples of C38-targeting covalent MGs was the mercaptodiamine WR-1065, which is the active metabolite of radioprotective prodrug amifostine (Figure 1A).^34^ Formation of a disulfide bond between C38 and WR-1065 led to a modest 2.8-fold stabilization of the 14–3–3/ERα PPI. This was further enhanced by FC-A binding into its typical pocket (Figure 1B).^34^

The fragment-like properties of WR-1065 and its distinct binding pose made it an attractive starting point for optimization into MGs. In addition to isoform selectivity, it provided an opportunity to develop MGs that could target 14–3–3 clients with recognition motifs that do not reveal a composite ligand binding pocket. Here, we report on how a rational design approach to optimize WR-1065 potency led to the unexpected discovery of a reversible aminoacrylamide-containing MG (**7**) for 14–3–3 PPIs that has a mode of action which is specific to dimeric 14–3–3.

A principal objective of the design strategy was to replace the reversible disulfide covalent tether WR-1065 made to 14–3–3σ C38 with an irreversible cysteine modification because it was predicted this would lead to improved potency. A series of structural analogues of WR-1065 were synthesized that retained the pharmacophorically important primary amine but varied the electrophilic warhead (2-chloro-5-nitrobenzamide, iodoacetamide, maleimide, vinyl sulfonamide, acrylamide) and chain length (**1**–**8**, Figure 2A). A fluorescence polarization substrate-binding assay was used to determine the stabilizing effect of compounds **1**–**8**. 14–3–3σ was titrated to a fixed concentration (10 nM) of a TAMRA-labeled synthetic 8 amino acid peptide that mimicked the ERα C-terminal pT584 14–3–3 recognition motif (herein referred to as TAMRA-ERα). This established a baseline affinity of 14–3–3σ for TAMRA-ERα defined by an apparent dissociation constant (*k*_d_-App) of 206 ± 31 nM (Figure 2B). The experiment was repeated in the presence of electrophilic WR-1065 analogues **1–8** (1.0 mM) to determine if they stabilized the 14–3–3/TAMRA-ERα interaction as indicated by a reduction in *k*_d_-App relative to the control. The 2-chloro-5-nitrobenzamide (**1**), iodoacetamide (**2**), and maleimide (**3**, **4**) analogues did not show any significant stabilization effect. However, the vinyl sulfonamide (**6**) and acrylamide analogues (**7**, **8**) had a far greater stabilizing effect compared to WR-1065, with a 22×, 76×, and 26× reduction in *k*_d_-App, respectively (Figure 2B).

**Figure 2:**
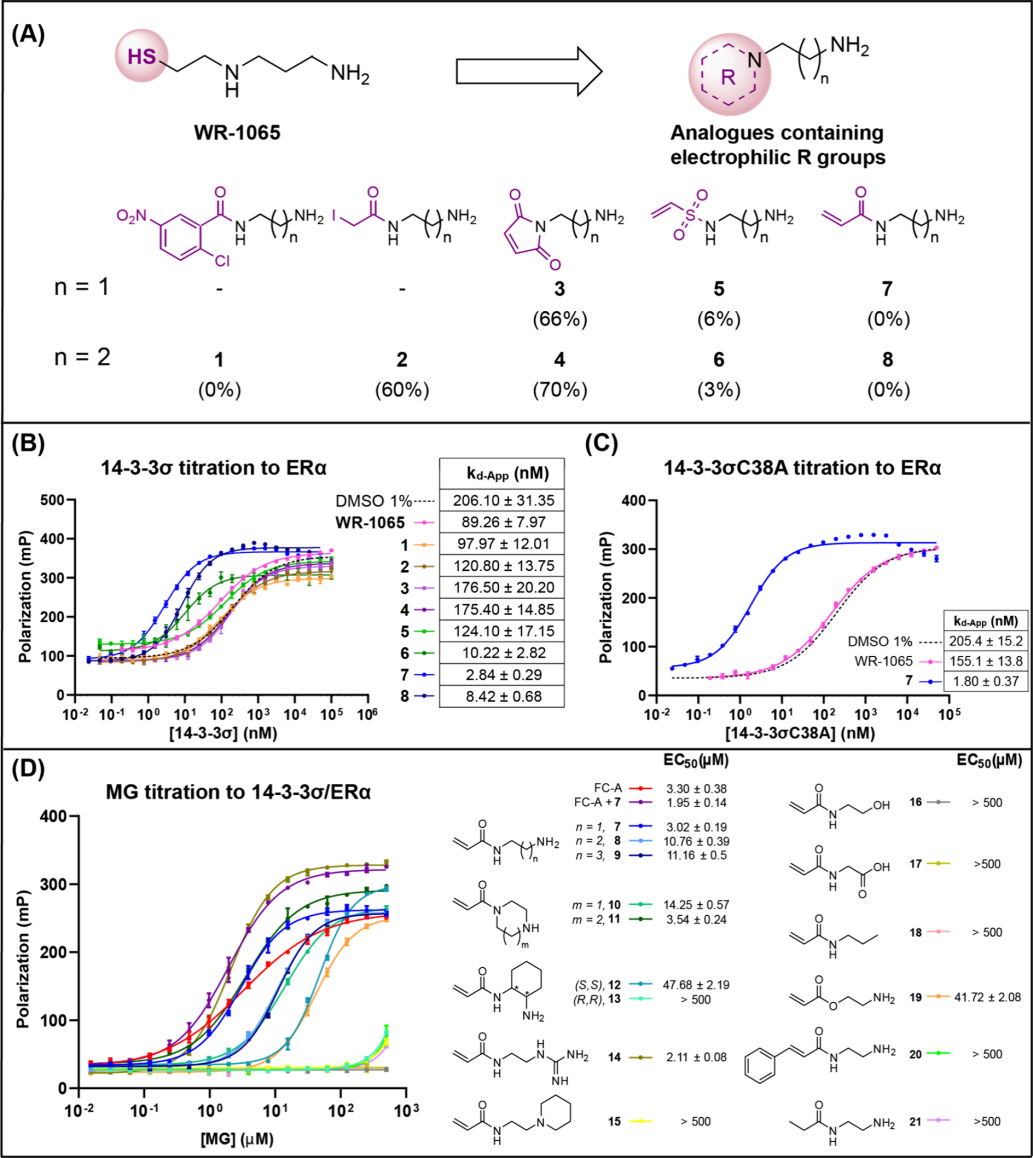
(A) Structure of electrophilic analogues to WR-1065. Percentage of covalent binding to 14–3–3σ, assessed by LCMS, shown in parentheses below each structure. Compounds with variable chain length are indicated by *n* = 1 or *n* = 2. (B) FP binding curves for the titration of 14–3–3σ to TAMRA-ERα (10 nM) in the presence of MGs (1 mM). (C) FP binding curves for the titration of 14–3–3σC38A to TAMRA-ERα (10 nM) in the presence of WR-1065 and **7** (1 mM). (D) FP binding curves for the titration of MGs to 14–3–3σ (50 nM) and TAMRA-ERα (10 nM).

Liquid chromatography – mass spectrometry (LCMS) was used to confirm covalent modification of 14–3–3σ by electrophiles **1–8** (Figure 2A, Figures S1–S15). Contrary to our expectation, the active acrylamides (**7**, **8**; 1 mol equiv) showed no protein modification after incubation with apo-14–3–3σ after 24 h (Figures S12, S15). To determine if covalent modification was dependent on binary complex formation, the same experiments were conducted in the presence of an unlabeled ERα peptide (1:1 molar ratio of 14–3–3σ:ERα) at 1 and 10 mol equiv of **7** (Figures S13–S14). However, still no covalent modification of 14–3–3 was observed. The inactive iodoacetamide (**2**) and maleimides (**3**, **4**) efficiently covalently modified 14–3–3 under the same conditions. A lack of reactivity for C38 toward acrylamides has been previously reported,^35^ but this warhead also generated covalent hits in a cysteine activity-based protein profiling screen^36^ and was used to generate covalent fusicoccin analogues.^37^ The reactivity of C38 toward different electrophiles, therefore, appears to be ligand-specific. To confirm that C38 was not critical to the activity of the most effective MG, acrylamide (**7**), further FP binding experiments were conducted using a 14–3–3σ C38A mutant and the 14–3–3ζ isoform which lacks C38. Acrylamide (**7**) retained its MG activity in both cases, exhibiting a 114-fold and 63-fold reduction in *k*_d_-App, respectively, whereas the cognate mercaptodiamine WR-1065 was inactive (Figure 2C). Thus, the MG activity of aminoacrylamide (**7**) has a reversible and C38-independent mode of action.

A structure–activity relationship (SAR) study of acrylamide (**7**) was conducted to establish the MG pharmacophore. Structural analogues of (**7**) were synthesized and screened using a ligand-titration FP experiment whereby compounds were titrated to fixed concentrations of 14–3–3σ (50 nM) and TAMRA-ERα (10 nM) to determine EC_50_ values (Figure 2D). Acrylamide (**7**) (EC_50_ = 3.0 ± 0.2 μM) had potency comparable to that of FC-A (3.3 ± 0.4 μM). Increasing the chain length between the acrylamide group and the basic amine was detrimental to activity (**8–9**), as was rigidifying the linker as a piperazine (**10**). However, the larger azepane (**11**) retained comparable activity to the initial hit (**7**). The introduction of stereochemically restricted linkers caused a 16-fold loss in activity for the (*S,S*)-1,2-diaminocyclohexyl derivative (**12**) and no quantifiable stabilization for the (*R,R*)-1,2-diaminocyclohexyl derivative (**13**). Changing the primary amine group to a guanidine moiety (**14**) slightly increased stabilization by 1.5-fold, while the substitution of the primary amine to a tertiary amine within a piperidine ring (**15**) led to loss of MG activity. The importance of a basic amine group was further confirmed by compounds **16**–**18** whereby replacement of the primary amine with hydroxyl, carboxylate, and alkyl groups led to complete loss of MG activity. The acrylamide group was also critical for activity: the aminoethyl acrylate derivative (**19**) was 14 times less potent than acrylamide (**7**), while substitution of the acryloyl by a cinnamoyl (**20**) or a propionyl group (**21**) led to a complete loss of activity. The MG pharmacophore of (**7**), therefore, required both a primary basic amine and acrylamide functionality connected by a short flexible linker.

To further validate the MG effect of acrylamide (**7**), a series of 14–3–3 titration FP experiments were performed in the presence of increasing concentrations of **7** (1 – 100 μM, Figure 3A). Maximal stabilization was reached in the presence of **7** at 10 μM. Intriguingly, at intermediate concentrations close to the observed EC_50_ value (2 – 5 μM), the binding curves displayed biphasic characteristics (Figure 3A). Similar observations have previously been reported with respect to 14–3–3 binding to peptide substrates whereby diphosphorylation, and the dimeric nature of 14–3–3, are important for affinity.^17,18^ The precise explanation for these observations in the context of FP is a source of debate, but the data point toward a mechanism of action for acrylamide **7** that is related to the 14–3–3 dimeric interface. Indeed, ligands containing positively charged basic functionality have previously been shown to target the 14–3–3 dimer interface^20^ leading to both dimer stabilization^21^ and dimer disruption.^22^ If acrylamide **7** interacts with the 14–3–3 dimeric interface, then it should not (directly) compete for binding with the well-characterized MG FC-A which targets the 14–3–3 binding groove. To this end, an FP protein titration experiment was conducted in the presence of both MGs (Figure 3B). This gave rise to an additive stabilization effect which is indicative that the two MGs have different binding sites and complementary MG effects.

**Figure 3:**
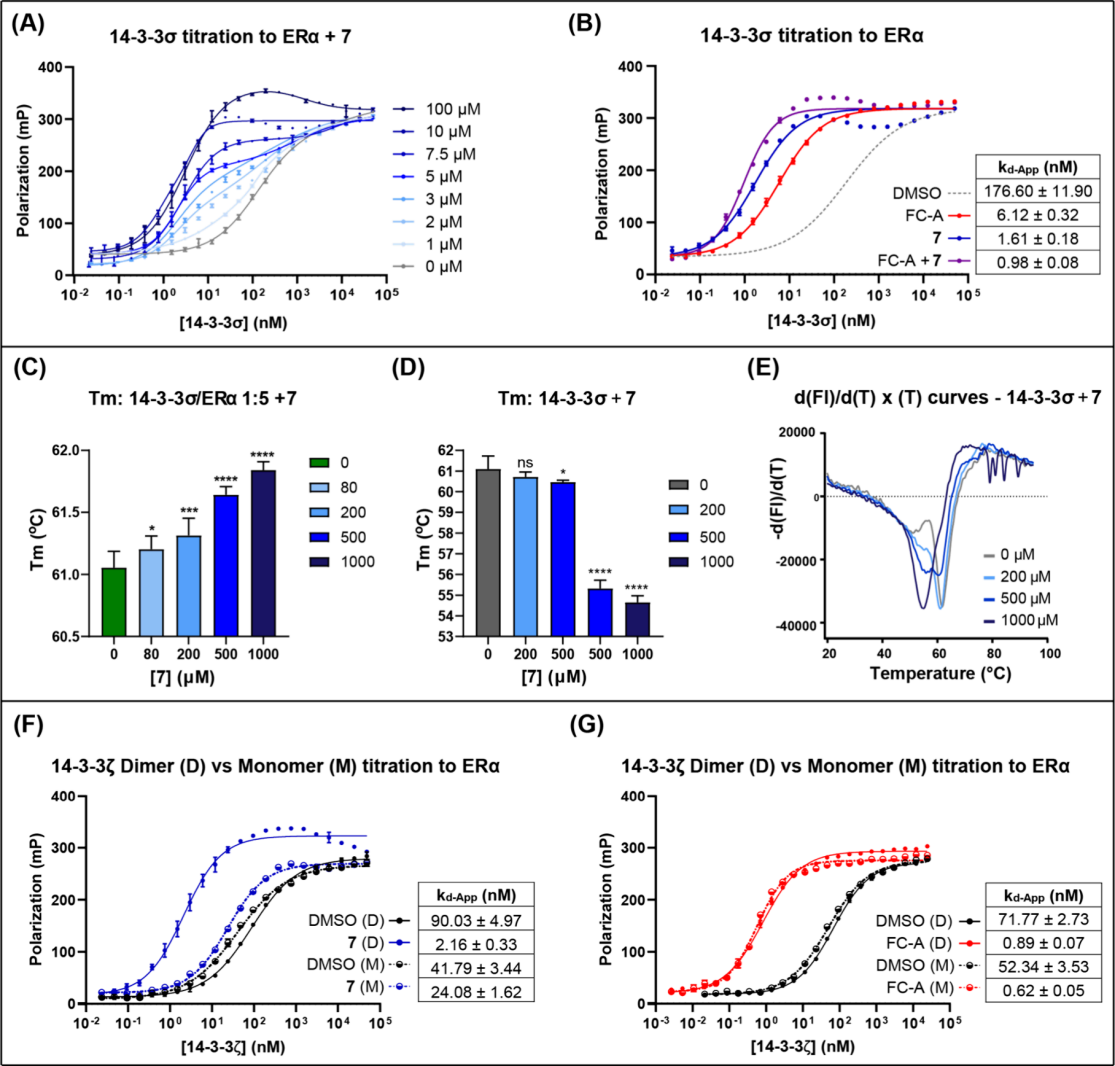
(A) FP binding curves for the titration of 14–3–3σ to TAMRA-ERα (10 nM) in the presence of **7** (0–100 μM) and biphasic curve fitting (R^2^ > 0.99). (B) FP binding curves for the titration of 14–3–3σ to TAMRA-ERα (10 nM) in the presence of **7** and/or FC-A and/or (10 μM). (C) Changes in melting temperature (Tm) for the 14–3–3σ (25 μM)/ERα (125 μM) complex upon treatment with increasing concentrations of aminoacrylamide **7** (Dunnett’s test: ns = *P* > 0.1; * = *P* < 0.1, ** = *P* < 0.01, *** = *P* < 0.001, **** = *P* < 0.0001). (D) Changes in melting temperature (Tm) for apo 14–3–3σ (25 μM) in the presence of **7**. (E) First derivative curves [d­(F)/d­(T)] for the thermo shift assay showing the melting points for apo 14–3–3σ in the presence of **7** (0–100 μM). (F) FP binding curves for the titration of dimeric 14–3–3ζ WT (D) and monomeric mutant 14–3–3ζ ^12^LAE^14^-^12^QQR^14^ (M) to TAMRA-ERα (10 nM) in the presence or absence of **7** or FC-A (100 μM).

Compounds that modulate 14–3–3 dimerization could impact the protein stability. Ligand binding also usually influences protein thermal stability, and changes in protein melting temperature (Tm) can provide a strong indication if binary or ternary complexes are formed.^26,38,39^ To determine whether aminoacrylamide **7** modulated the structural stability of 14–3–3σ, a fluorescence-based thermal shift assay was used. The melting temperature (Tm) of apo 14–3–3σ (25 μM) was 60.1 °C, and this increased to 61.1 °C upon binary complex formation with the addition of an unlabeled ERα peptide (15 amino acids, 125 μM) (Figure S16). Adding increasing concentrations of aminoacrylamide **7** (80–1000 μM) to the binary complex led to a concentration-dependent increase in 14–3–3σ Tm up to 0.8 °C (Figure 3C), suggestive of the formation of a stabilized ternary complex. Intriguingly, treatment of apo 14–3–3σ with high concentrations of **7** (500–1000 μM) caused a decrease in 14–3–3σ Tm from 61.1 to 54.7 °C (Figure 3D). At the intermediate concentration of **7** (500 μM), two melting events were observed simultaneously suggesting two distinct protein denaturation pathways (Figure 3E). This could indicate a consequence of acrylamide **7** influencing the 14–3–3 dimeric interface, leading to dimer dissociation at a lower temperature relative to global protein denaturation.

Monomeric 14–3–3ζ retains the WT protein’s ability to bind to biologically relevant phosphorylated partners.^40,41^ However, if the MG activity of **7** was dimer-dependent, it should have no effect on the interactions of monomeric 14–3–3ζ. To investigate this, both WT (dimeric) and mutant 14–3–3ζ proteins were used in an FP assay. In the mutant 14–3–3ζ the key salt bridges stabilizing the 14–3–3ζ dimer are disrupted (^12^LAE^14^ was mutated to ^12^QQR^14^) resulting in a monomeric protein.^40^ Titration of WT (dimeric) 14–3–3ζ to a fixed concentration of TAMRA-ERα (10 nM) in the presence of acrylamide **7** (100 μM) resulted in a 41-fold increase in binding affinity to ERα compared to the control (*k*_d_-App = 90.0 ± 5.0 → 2.2 ± 0.3 μM Figure 3F). In stark contrast, when mutant (monomeric) 14–3–3ζ was used in the same experiment, only a small 1.7-fold stabilization was observed (*k*_d_-App = 41.8 ± 3.4 → 24.1 ± 1.6 μM, Figure 3F). Treatment of both dimeric and monomeric 14–3–3 with FC-A (100 μM) resulted in an 80- and 84-fold stabilizing effect, respectively, further confirming the integrity of the 14–3–3 binding groove in the monomeric protein (Figure 3G). These data provide a further strong indication that the MG activity of acrylamide **7** results from interaction with and/or modulation of the 14–3–3 dimeric interface.

Selective stabilization of the interactions between 14 and 3–3 proteins and specific clients poses a significant challenge. To establish if acrylamide **7** displayed any selectivity for certain 14–3–3 clients, the binding of 14–3–3σ to three fluorescently labeled phosphopetides mimicking distinct 14–3–3 binding motifs was investigated using the FP assay. The LRRK2 (Cy5-LRRK2_928–942_^pS935^) binding motif extends through the 14–3–3 binding groove. This obscures the FC-A pocket, and, thus, LRRK2 is not stabilized by FC-A.^42^ p53 (TAMRA-p53_379–393_^pT387^) adopts a unique turn conformation within the binding groove which has limited complementarity with FC-A binding, and thus it is only stabilized to a small degree.^43^ AHA2 (Cy5-AHA2_384–398_^pT397^) has a mode 3 binding motif analogous to that of ERα, and FC-A induces a large stabilizing effect.^44^

14–3–3σ was titrated with the three peptides (10 nM). In the absence of a MG, the *k*_d_-App for 14–3–3 binding to LRRK2, p53, and AHA2 was 5544 ± 818 nM, 1142 ± 321 nM, and 37.5 ± 2.8 nM, respectively (Figure 4A-C). As expected, FC-A (500 μM) had no effect on the LRRK2 interaction, induced moderate stabilization of the p53 interaction (9.8-fold), and significant stabilization of the interaction with AHA2 (89-fold) (Figure 4A-C). Acrylamide **7** had a different selectivity profile, consistent with the hypothesis that it has a mechanism of action distinct from that of FC-A. Acrylamide **7** (500 μM) had a significant stabilizing effect on the interaction with LRRK2 (39-fold), no effect on the p53 interaction, and only a moderate effect on the interaction with AHA2 (4.5-fold). The AHA2 interaction was stabilized by both MGs, and thus, the effect of the two ligands in combination was investigated (Figure 4C). Surprisingly, and in contrast to the additive effect observed for ERα, acrylamide **7** had a deleterious effect on FC-A stabilization of the AHA2 interaction. Direct competition would most likely be observed for both clients, and therefore this observation could point to a client-specific allosteric effect.

**Figure 4:**
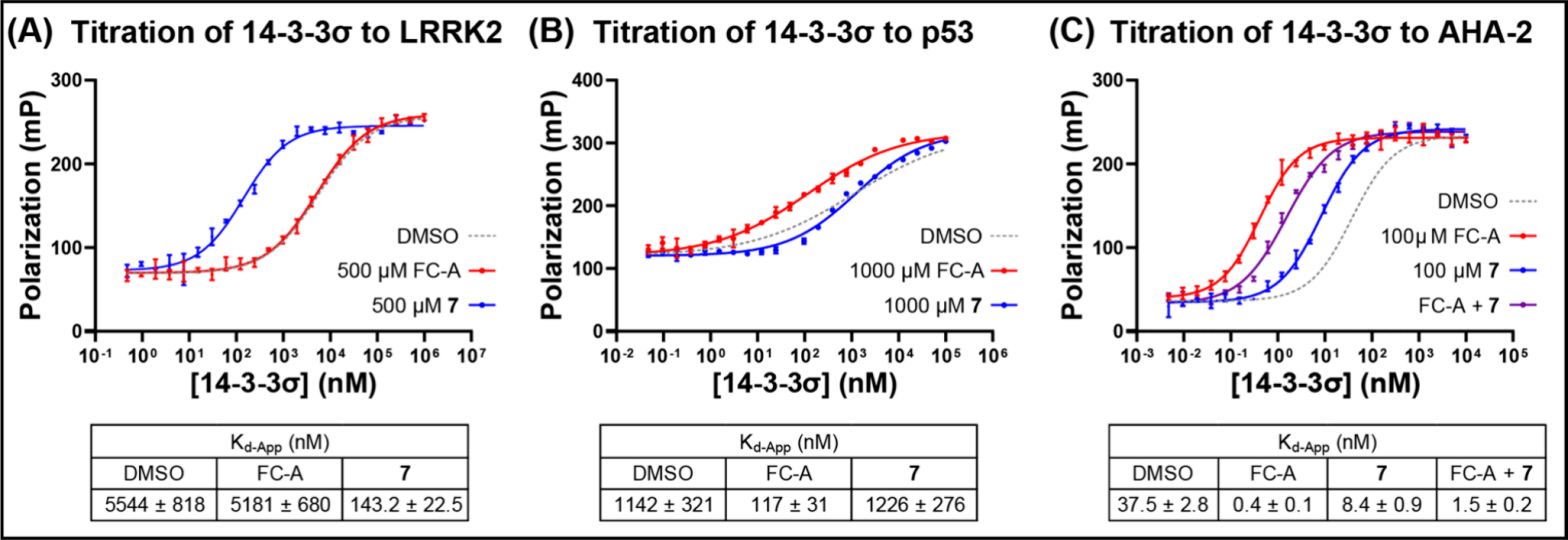
FP binding curves for the titration of 14–3–3σ to (A) LRRK2, (B) p53, or (C) AHA2 in the presence of FC-A (500 μM), **7** (500 μM), or both.

Despite very high degrees of sequence and structural homology, the seven 14–3–3 isoforms exhibit different binding affinities to the same client motifs.^45^ Similarly, individual 14–3–3 isoforms bind to subtly different binding motifs with differing affinities (e.g., *k*_d_-App for 14–3–3σ binding to the mode 3 ERα and AHA2 motifs in this work is 222 and 37 nM, respectively). Given that there is nearly 100% sequence homology in the 14–3–3 binding groove, it is possible that dimer interface dynamics, which differ between isoforms, play an important but poorly understood role in 14–3–3 client recognition. Thus, the selectivity profile observed by acrylamide **7** could reflect the sensitivity of different substrates to the 14–3–3 dimerization state and support the proposed mechanism of action. However, this aspect of 14–3–3 molecular biology is poorly understood, in part because the subtle conformational changes are not resolved by X-ray crystallography.

In conclusion, we report on the discovery and structure–activity relationship of aminoacrylamide **7** as a reversible MG for 14–3–3 PPIs. The core pharmacophore requires basic amine and acrylamide functionality but has a mode of action that is cysteine-independent. Thermal stability experiments indicate that aminoacrylamide **7** induces formation of a stabilized 14–3–3/client/MG ternary complex but destabilizes apo-14–3–3. The MG effect of **7** is only observed for dimeric 14–3–3 suggesting that **7** has an allosteric MG effect via interaction with the dimeric interface. This interaction appears to differentially modulate 14–3–3 interactions with clients containing different recognition motifs, supporting an emerging hypothesis that 14–3–3 dimerization is a critical aspect of molecular recognition. These findings provide further validation of the 14–3–3 dimeric interface as a viable allosteric target for 14–3–3 MGs. The evolution of MG discovery has been synonymous with serendipity and posthoc mode of action studies. Systematic, bottom-up approaches to MG discovery are important for accelerating development. However, as this work highlights, even rational design approaches can lead to unexpected outcomes that provide valuable alternative strategies for PPI stabilization.

## Supplementary Material



## Notes

Protein mass spectra; figures showing data for for fluorescence polarization and protein thermal stability control experiments; full experimental details; ^1^H, ^13^C and mass spectra for compounds **1–21** (PDF)
