# Supplementary material for: Discovery of Dimer-Dependent Aminoacrylamide Molecular Glues for 14–3–3 Protein–Protein Interactions
Source: ACS Med Chem Lett. 2026 Jan 12;17(2):476–83. doi: 10.1021/acsmedchemlett.5c00686 (PMC12907963; doi:10.1021/acsmedchemlett.5c00686)
Supplement: Supplementary file 1 [file ml5c00686_si_001.pdf]

Supporting Information

Discovery of Dimer-Dependent Aminoacrylamide Molecular Glues for 14-3-3 Protein-Protein Interactions

Paulo Pitasse-Santos,[1] Marta Falcicchio,[1] Rajdeep Sahota,[1] Hadeeqa G. Raza,[1,2] Aneika C. Leney,[2] Richard H. Cowan,[3] Gareth Hall,[3] Richard G. Doveston[1,*]

1. School of Chemistry and Institute of Structural and Chemical Biology, University of Leicester, University Road, Leicester, LE1 7RH, UK.
2. School of Bioscience, University of Birmingham, Edgbaston, Birmingham, B15 2TT, UK.
3. School of Biological Sciences and Institute of Structural and Chemical Biology, University of Leicester, University Road, Leicester, LE1 7RH, UK.

Table of Contents

1. Supplementary Figures
2. Chemistry experimental
3. Protein and peptide information
4. Biophysical assays
5. ^{1}H, ^{13}C, and HRMS spectra
6. References

Data Availability: All data are available on University of Leicester Figshare – DOI available on request (Email: r.g.doveston@leicester.ac.uk).

1. Supplementary Figures

1.1. Mass spectra: protein and molecular glues

14-3-3σ

Calculated Molecular Weight: 31014.49

Observed Mass: 31014.85

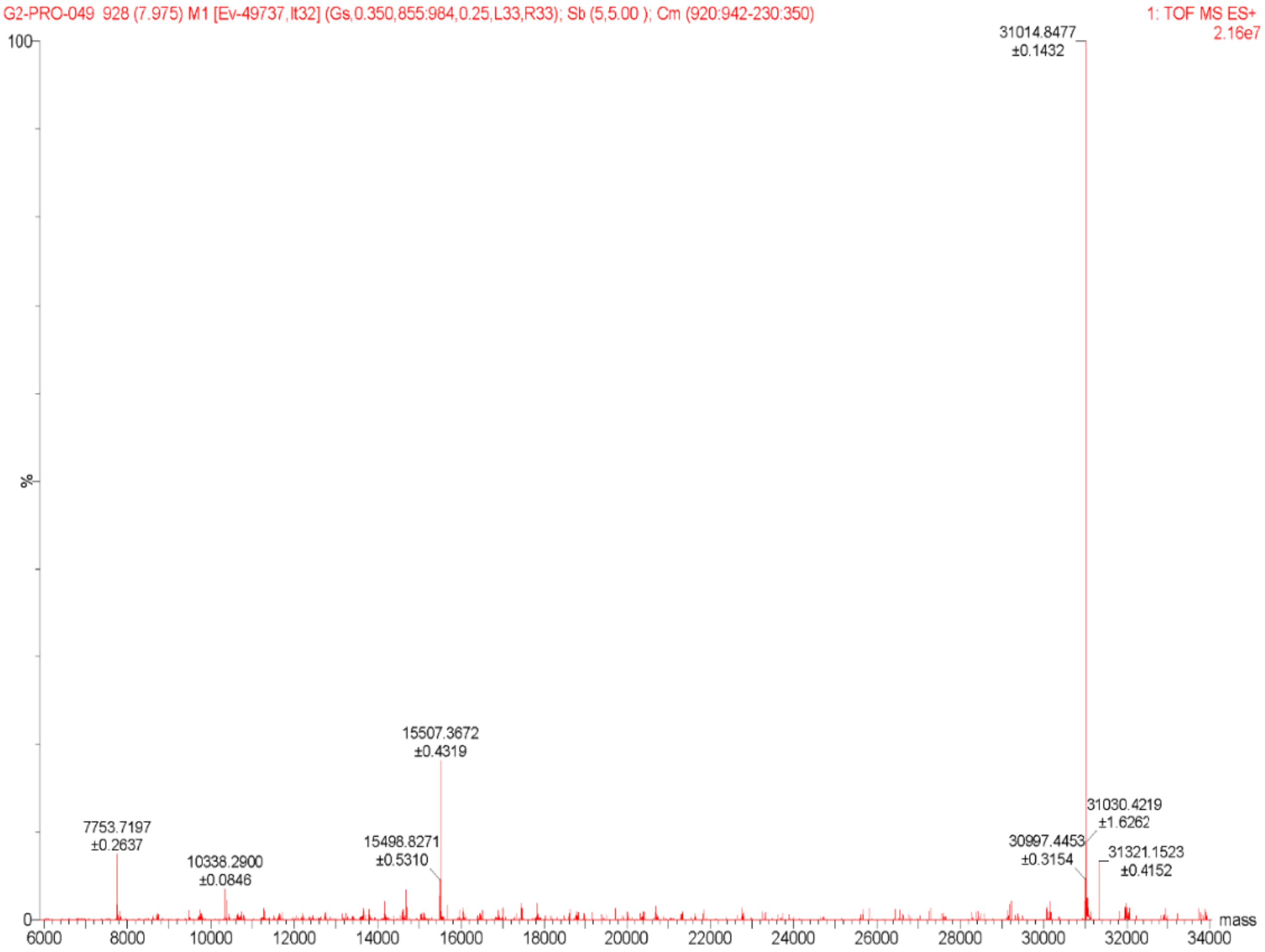

Figure S1. Deconvoluted spectrum of 14-3-3σ.

14-3-3σ + WR-1065

Obs.: WR-1065, MW = 134.2 Da, loss of 2H (MW = 2.0 Da) upon cysteine labelling.

Calculated Molecular Weight:

Protein: : 31014.49

Adduct, protein + WR-1065: 31146.73

Observed Mass: 31147.4

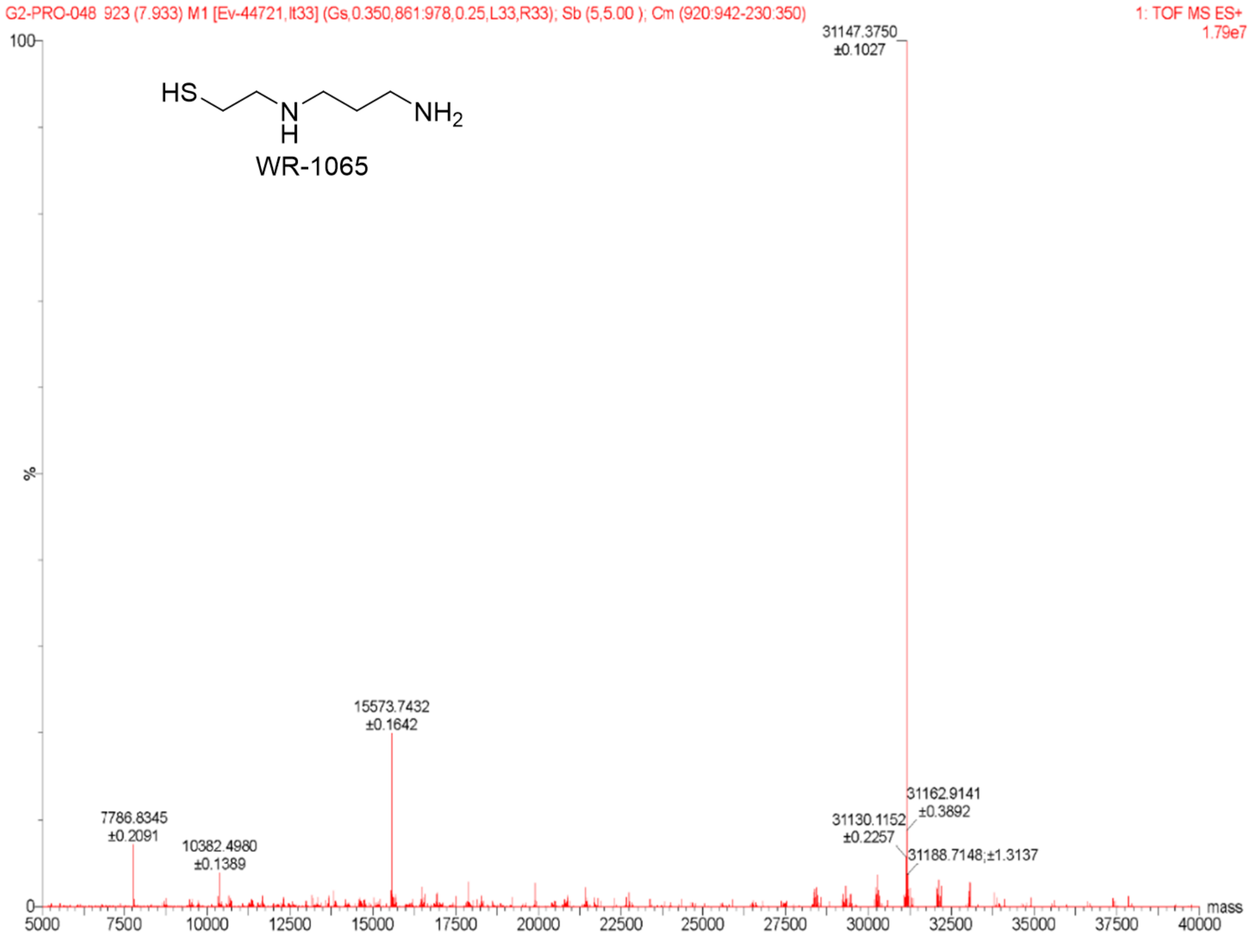

Figure S2. Deconvoluted spectrum of 14-3-3σ after incubation with WR-1065 (1 molar equivalent, 24 h).

14-3-3σ C38A
Calculated Molecular Weight: 30982.43
Observed Mass: 30982.16

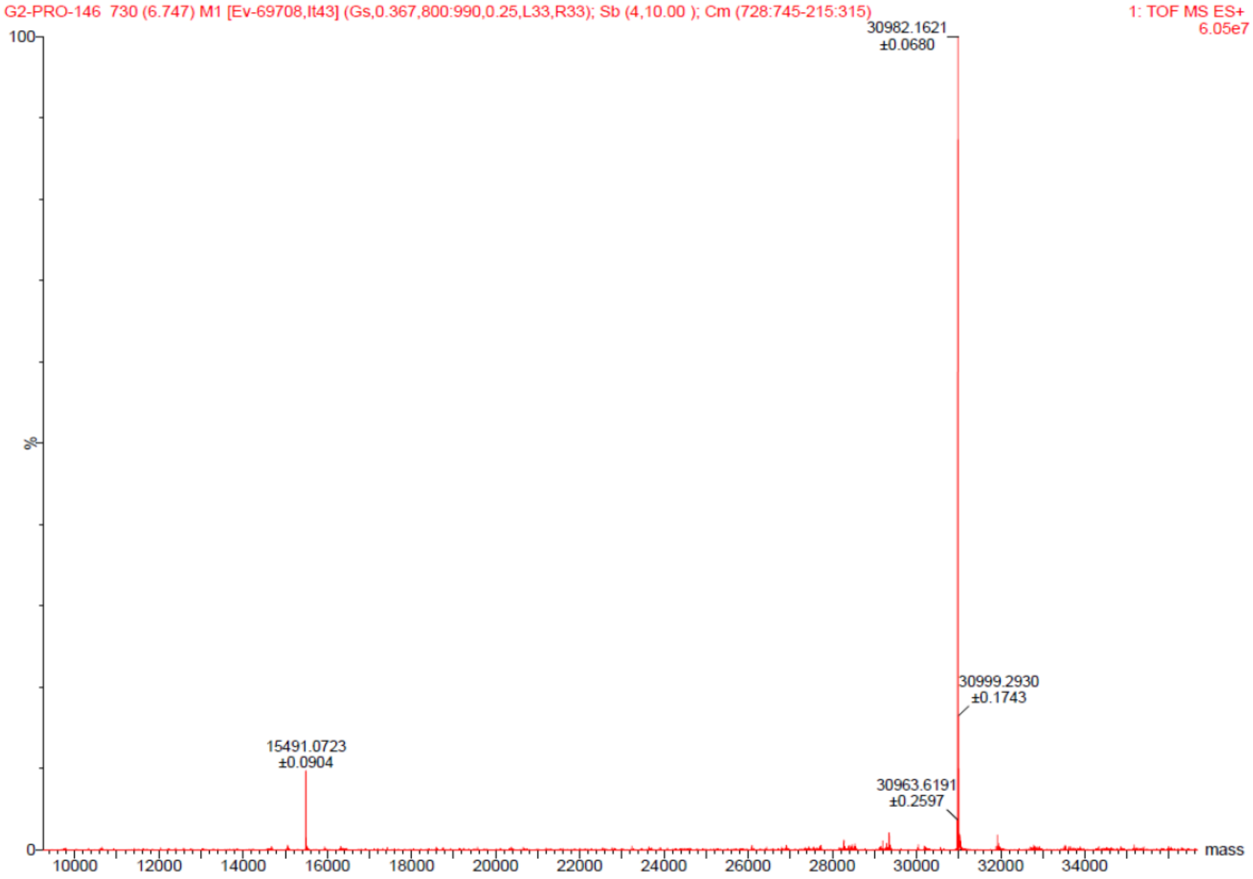

Figure S3. Deconvoluted spectrum of 14-3-3σ C38A.

14-3-3σ C38A + WR-1065

Obs.: WR-1065, MW = 134.2 Da, loss of 2H (MW = 2.0 Da) upon cysteine labelling.
Calculated Molecular Weight:
Protein: 30982.43
Adduct, protein + WR-1065: 31116.7

Observed Mass: 30982.3

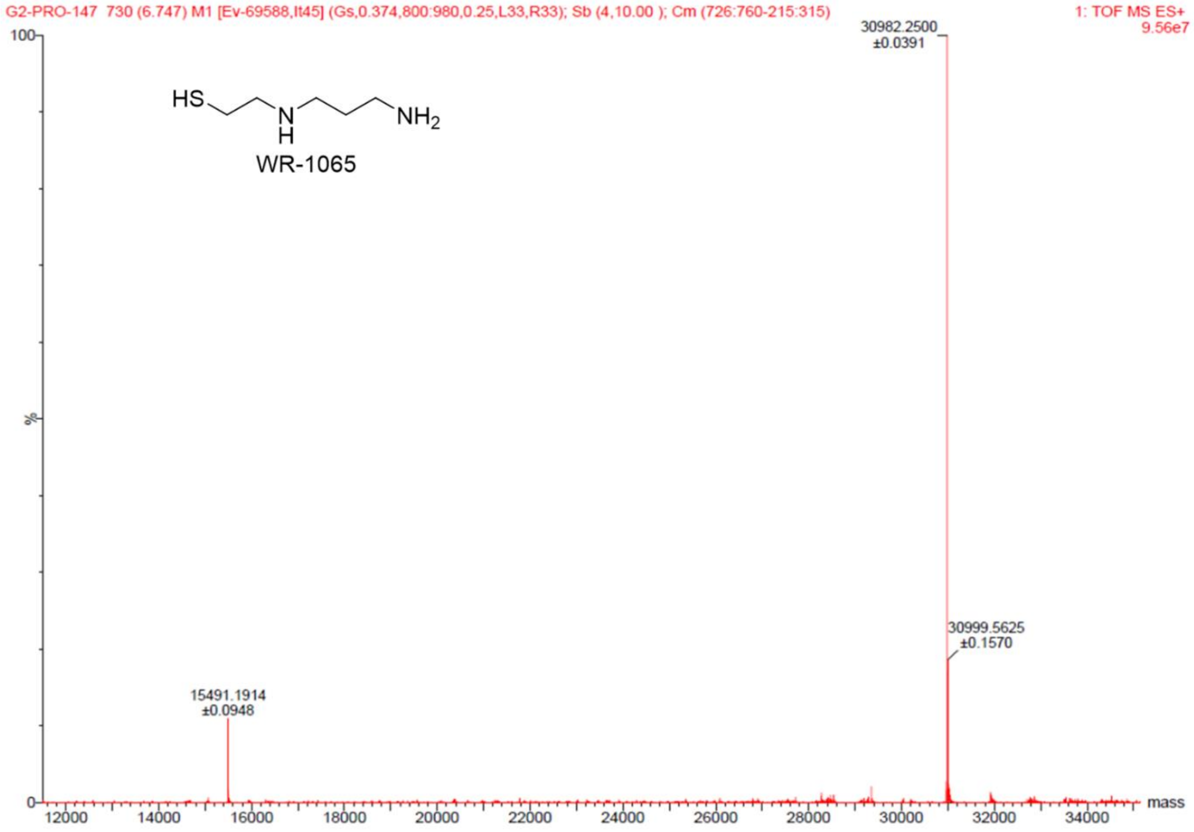

Figure S4. Deconvoluted spectrum of 14-3-3σ C38A after incubation with WR-1065 (1 molar equivalent, 24 h).

14-3-3σ + 1

Obs.: **1**, MW = 257.1 Da; loss of HCl (MW = 36.0 Da) upon cysteine labelling.

Calculated Molecular Weight:

Protein: 31014.49

Adduct, protein + **1**: 31116.7

Observed Mass: 31016.0

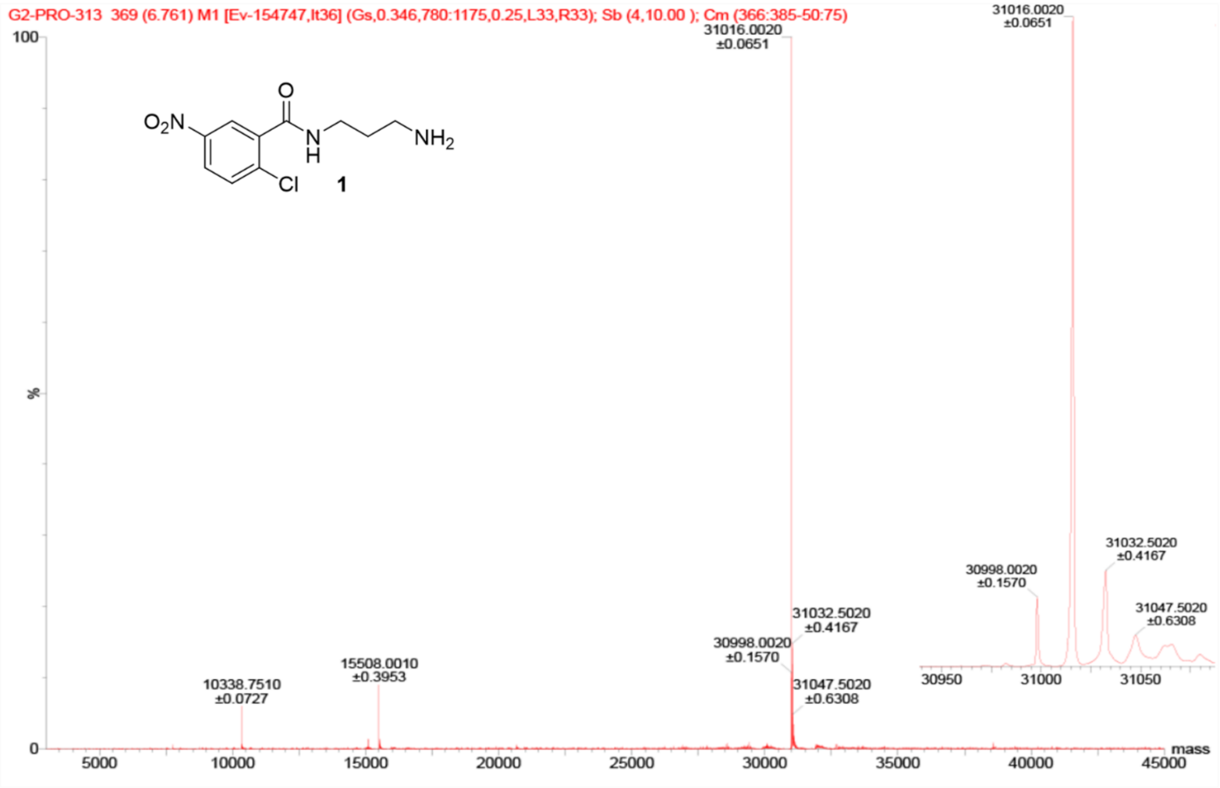

Figure S5. Deconvoluted spectrum of 14-3-3σ C38A after incubation with **1** (1 molar equivalent, 24 h).

14-3-3σ + 2

Obs.: **2**, MW = 242.0 Da; loss of HI (MW = 127.9 Da) upon cysteine labelling

Calculated Molecular Weight:

Protein: 31014.49

Adduct, protein + **2**: 31127.0

Observed Mass: 31129.5

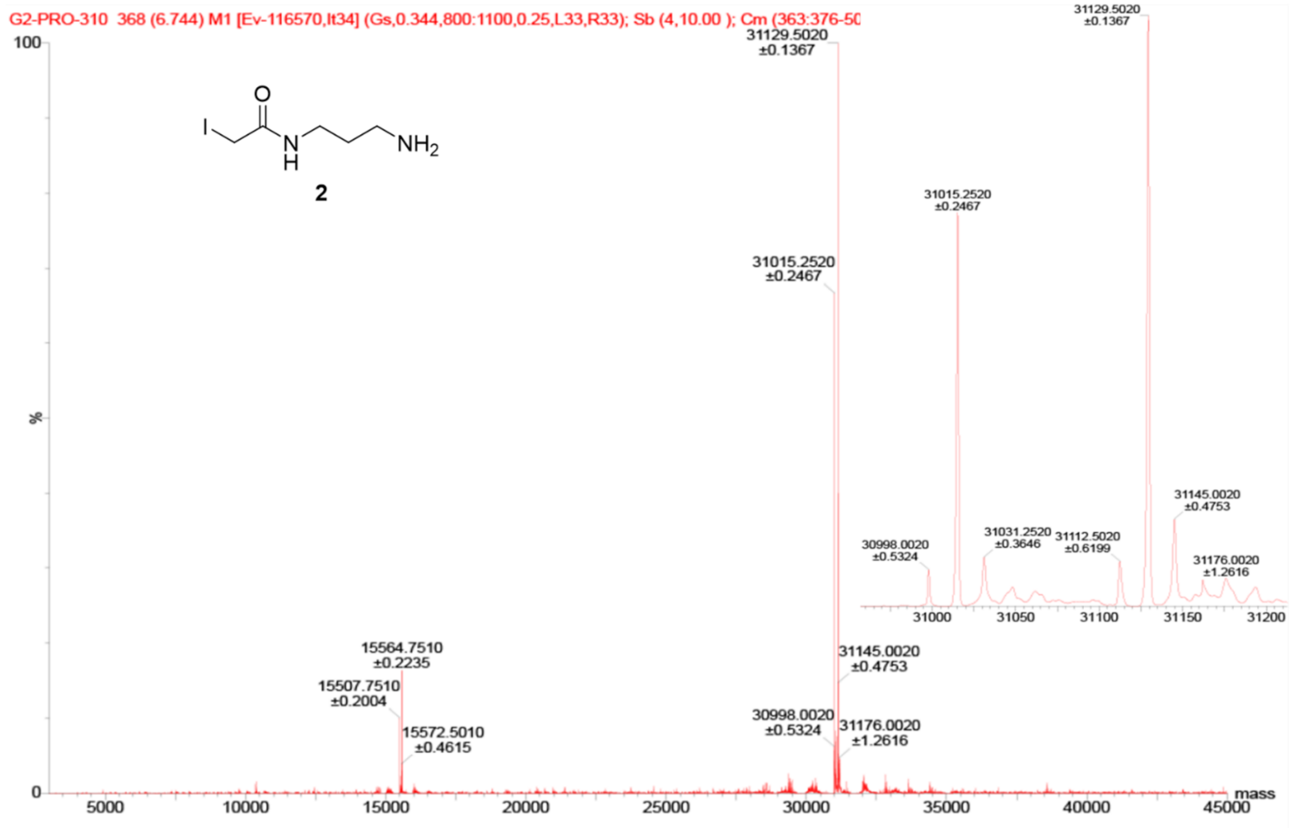

Figure S7. Deconvoluted spectrum of 14-3-3σ after incubation with **2** (1 molar equivalent, 24 h).

14-3-3σ + 3
Obs.: **3**, MW = 140.1 Da
Calculated Molecular Weight:
Protein: 31014.49
Adduct, protein + **3**: 31154.5

Observed Masses:
31015.3
31173.8 (adduct + water)

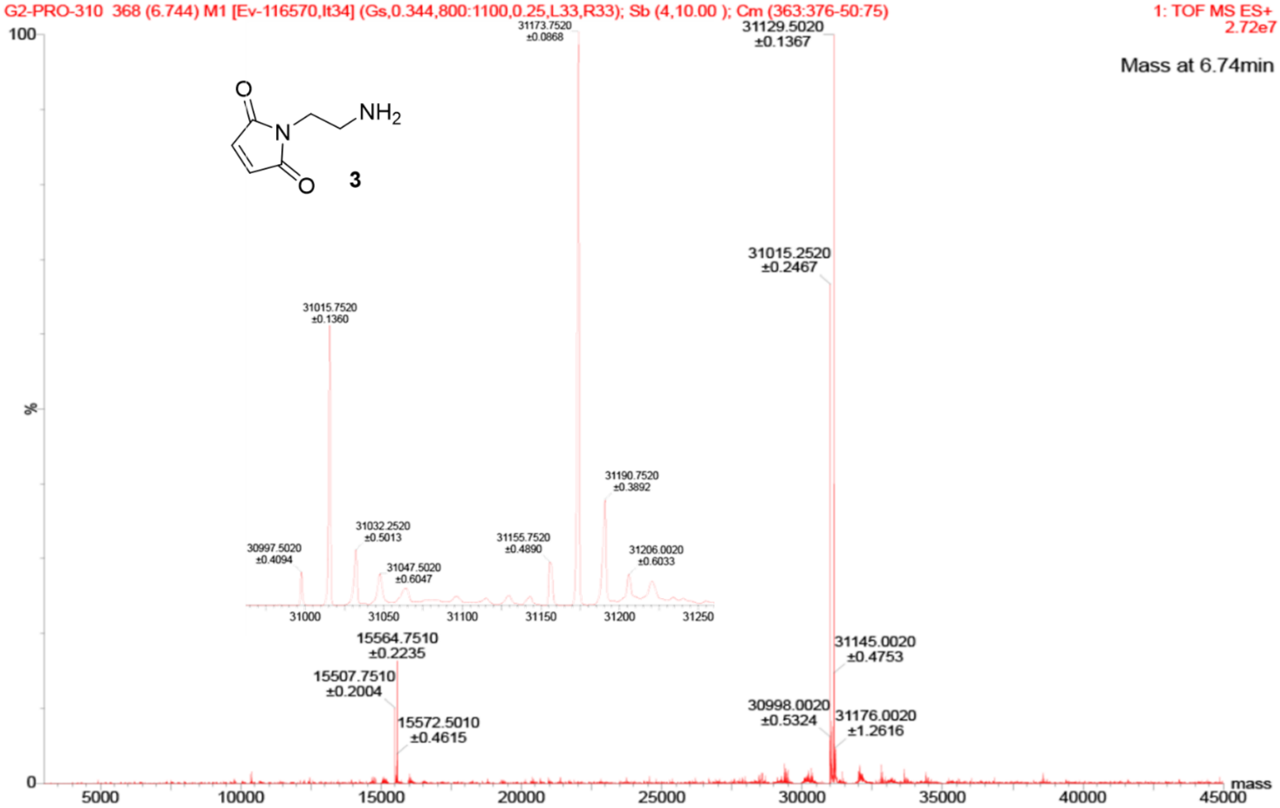

Figure S8. Deconvoluted spectrum of 14-3-3σ after incubation with **3** (1 molar equivalent, 24 h).

14-3-3σ + 4
Obs.: **4**, MW = 154.1 Da
Calculated Molecular Weight:
Protein: 31014.5
Adduct, protein + **4**: 31168.6

Observed Masses:
31014.0
31168.0

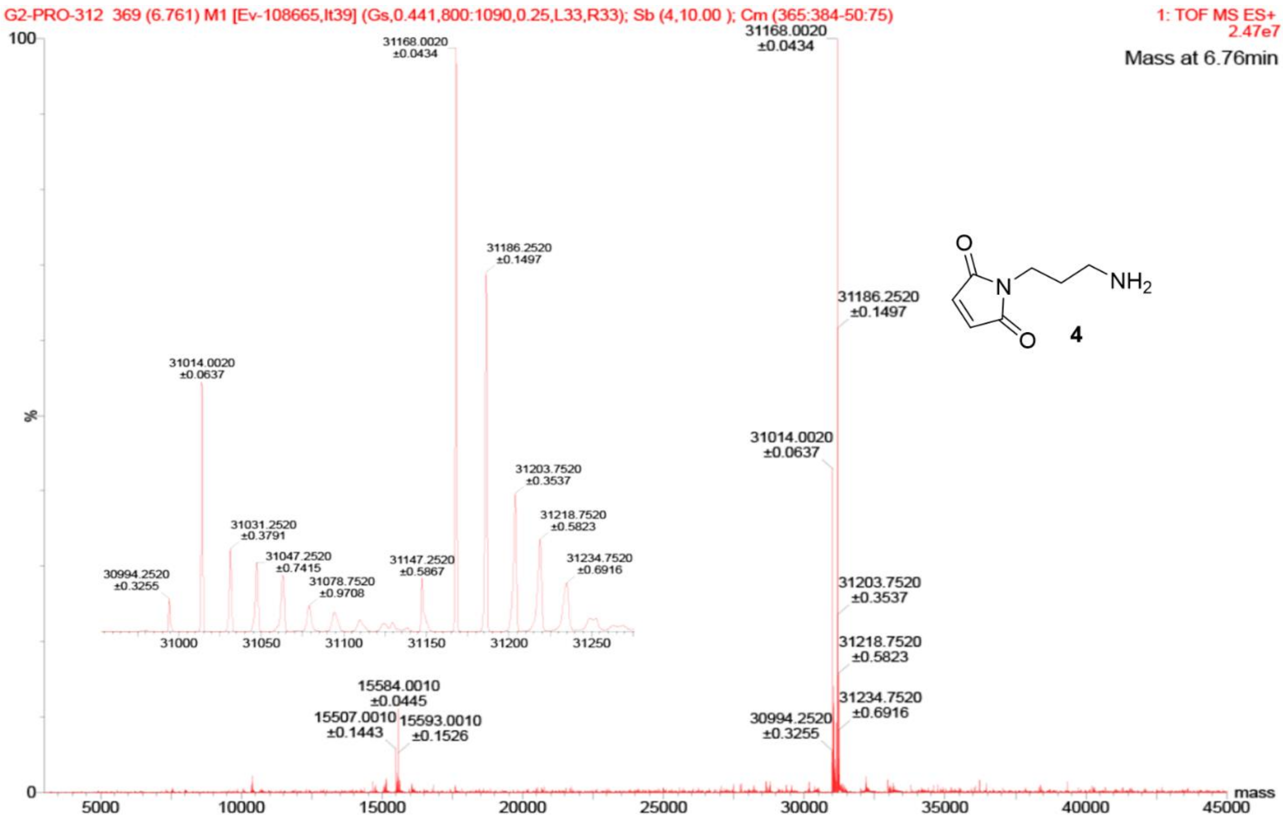

Figure S9. Deconvoluted spectrum of 14-3-3σ after incubation with **4** (1 molar equivalent, 24 h).

14-3-3σ + 5
Obs.: **5**, MW = 150.1 Da
Calculated Molecular Weight:
Protein: 31014.5
Adduct, protein + **5**: 31164.6

Observed Masses:
31015.0
31164.8.0

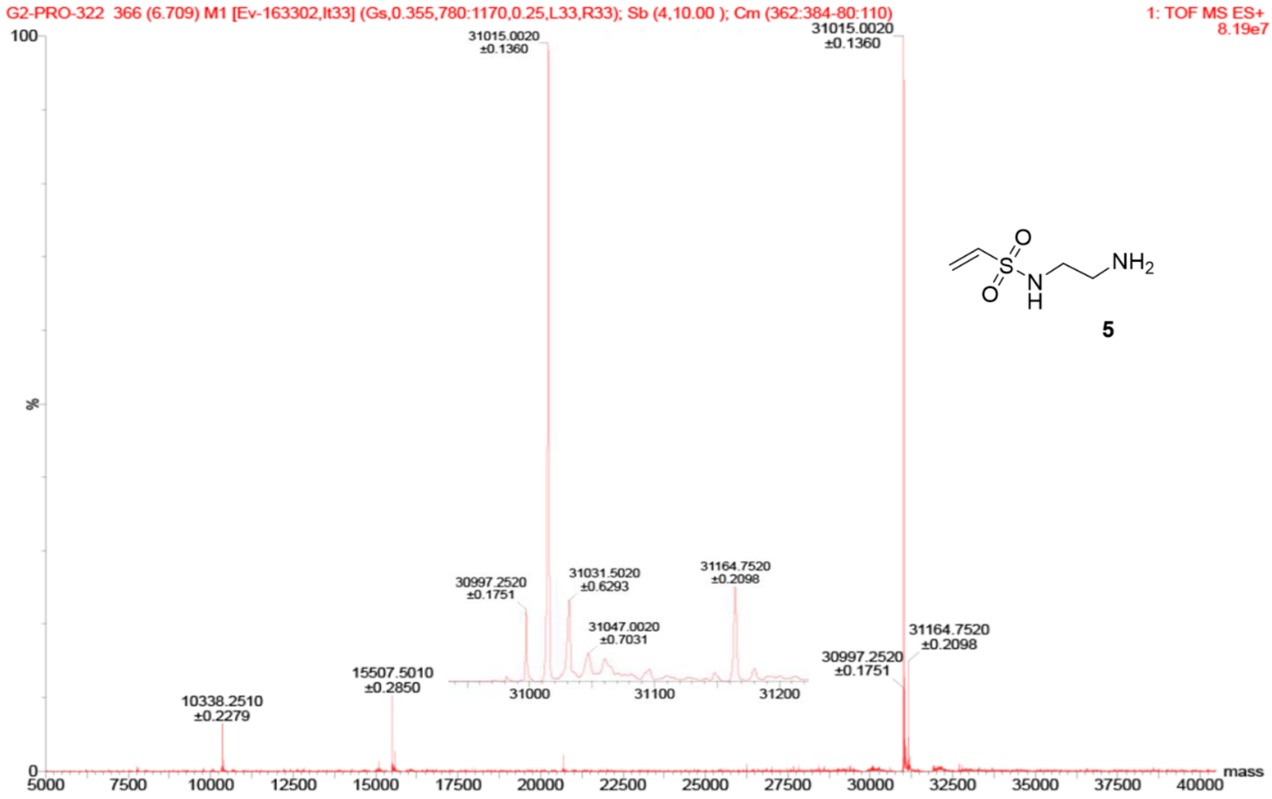

Figure S10. Deconvoluted spectrum of 14-3-3σ after incubation with **5** (1 molar equivalent, 24 h).

14-3-3σ + 6

Obs.: **6**, MW = 164.1 Da

Calculated Molecular Weight:

Protein: 31014.5

Adduct, protein + **6**: 31178.6

Observed Masses:

31014.8

31178.0

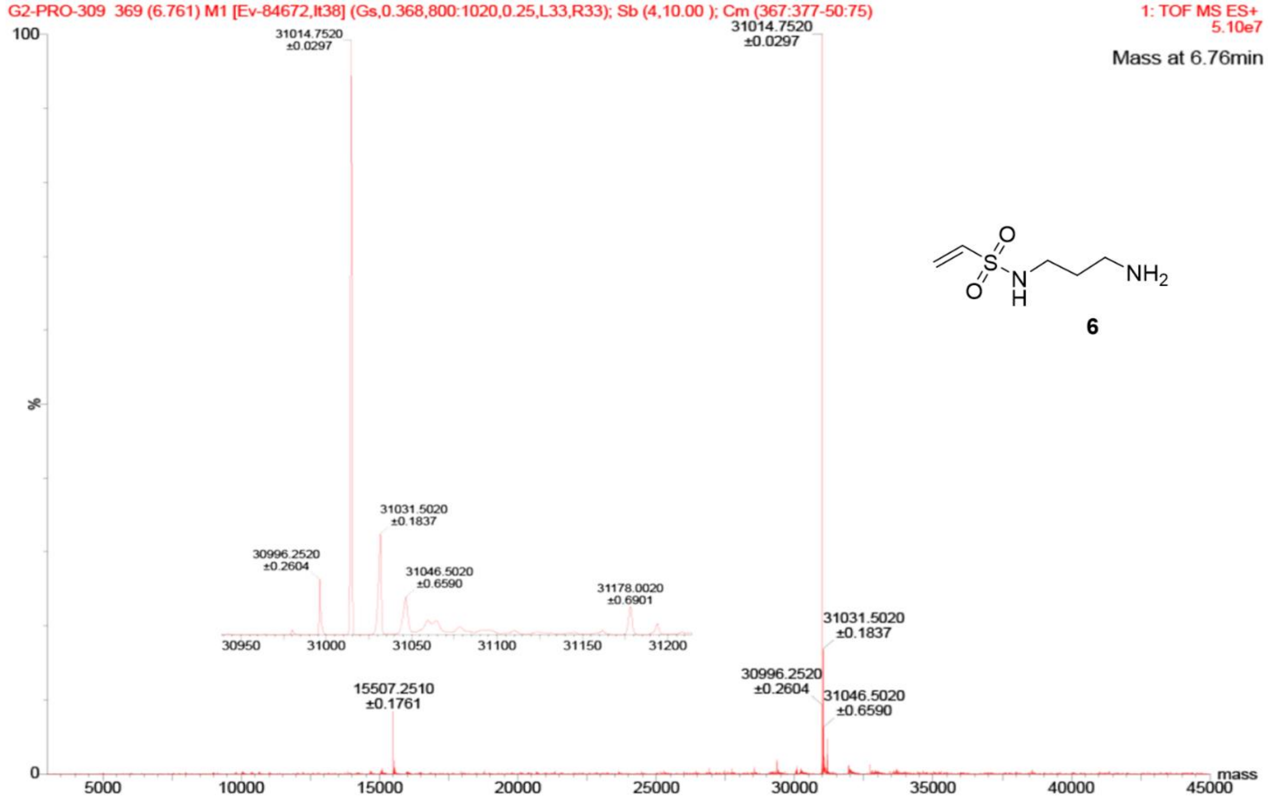

Figure S11. Deconvoluted spectrum of 14-3-3σ after incubation with **6** (1 molar equivalent, 24 h).

14-3-3σ + 7
Obs.: **7**, MW = 114.1 Da
Calculated Molecular Weight:
Protein: 31014.5
Adduct, protein + **7**: 31128.6

Observed Mass:
31014.8

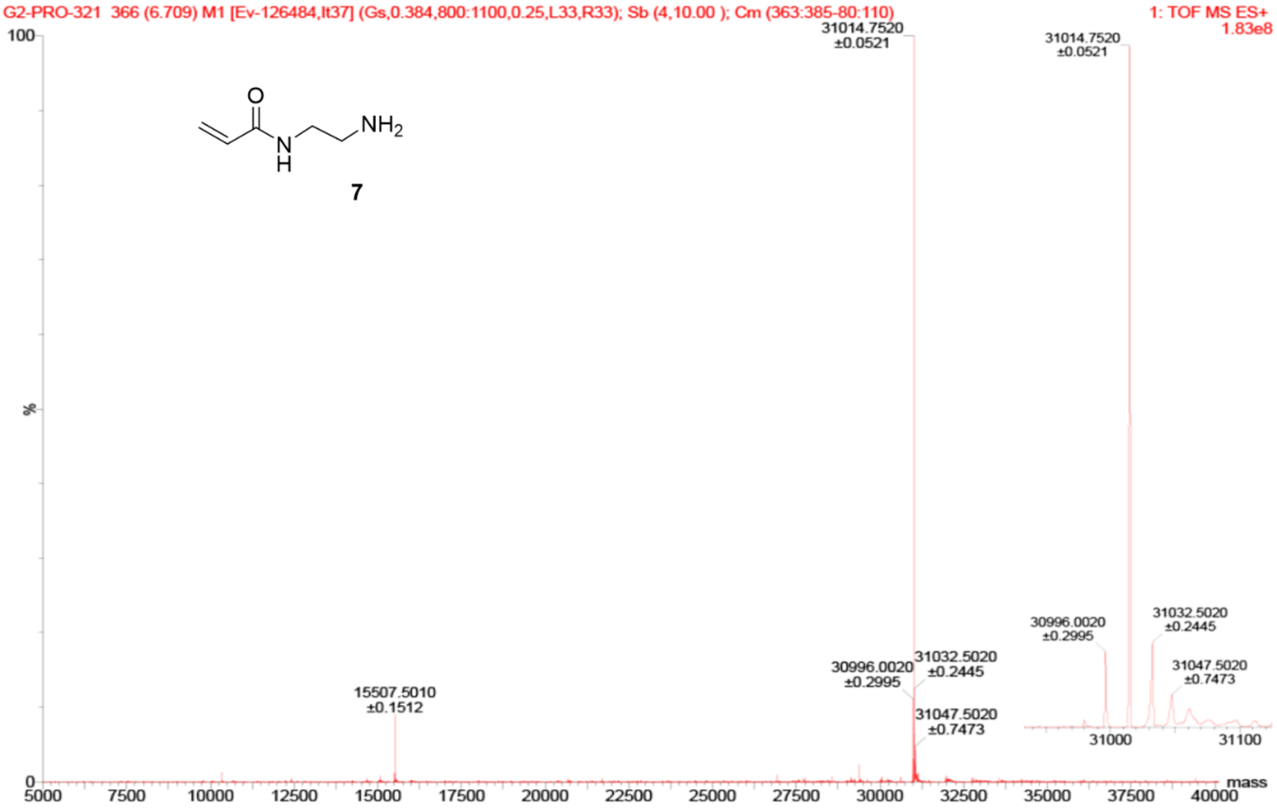

Figure S12. Deconvoluted spectrum of 14-3-3σ after incubation with **7** (1 molar equivalent, 24 h).

14-3-3σ + ERα + 7 (1:1:1)
Obs.: **7**, MW = 114.1 Da
Calculated Molecular Weight:
Protein: 31014.5
Adduct, protein + **7**: 31128.6

Observed Mass:
31014.8

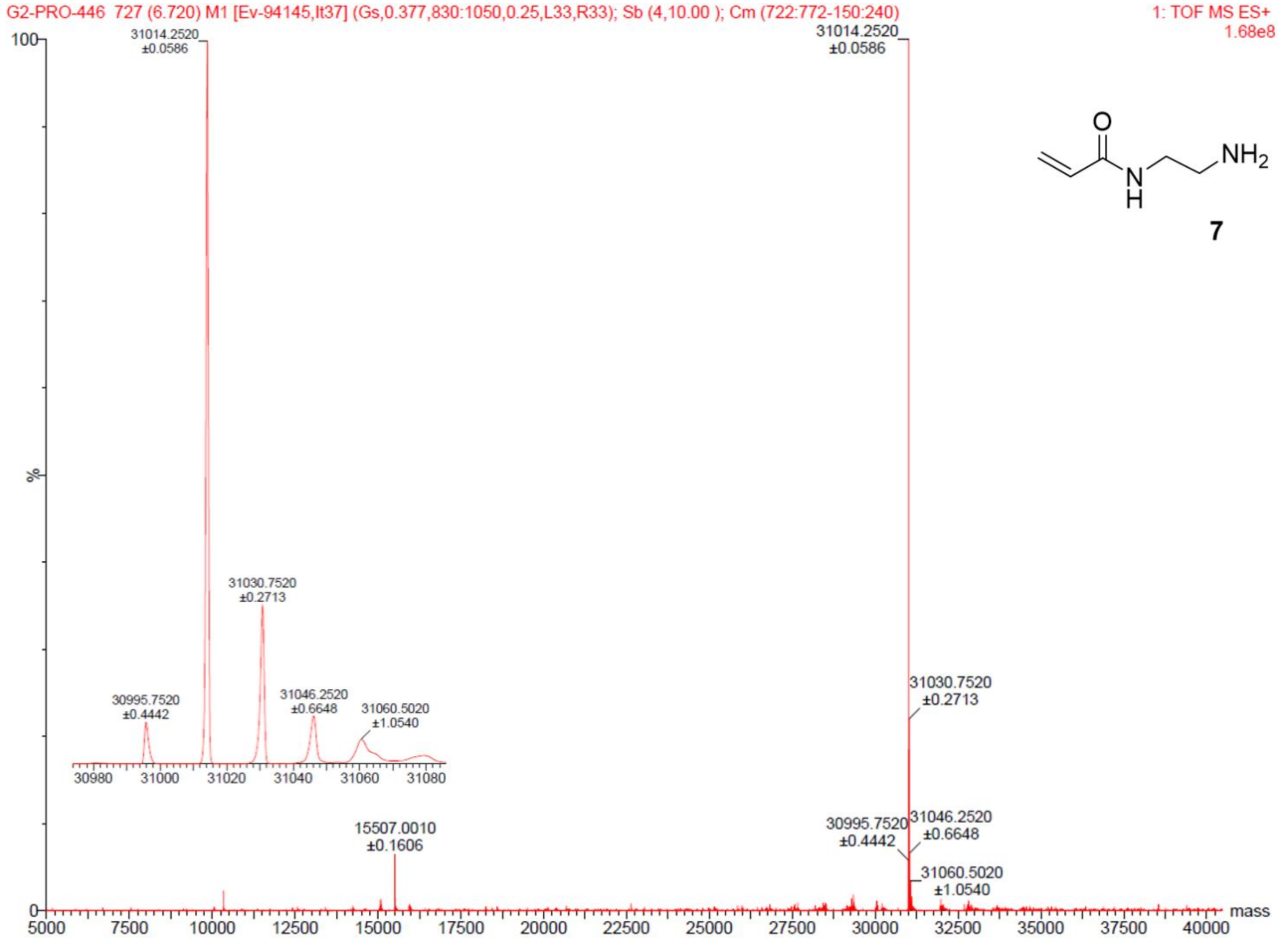

Figure S13. Deconvoluted spectrum of 14-3-3σ/ERα complex (1:1 molar ratio), after incubation with **7** (1 molar equivalent).

14-3-3σ + ERα + 7 (1:1:10)
Obs.: **7**, MW = 114.1 Da
Calculated Molecular Weight:
Protein: 31014.5
Adduct, protein + **7**: 31128.6

Observed Mass:
31014.8

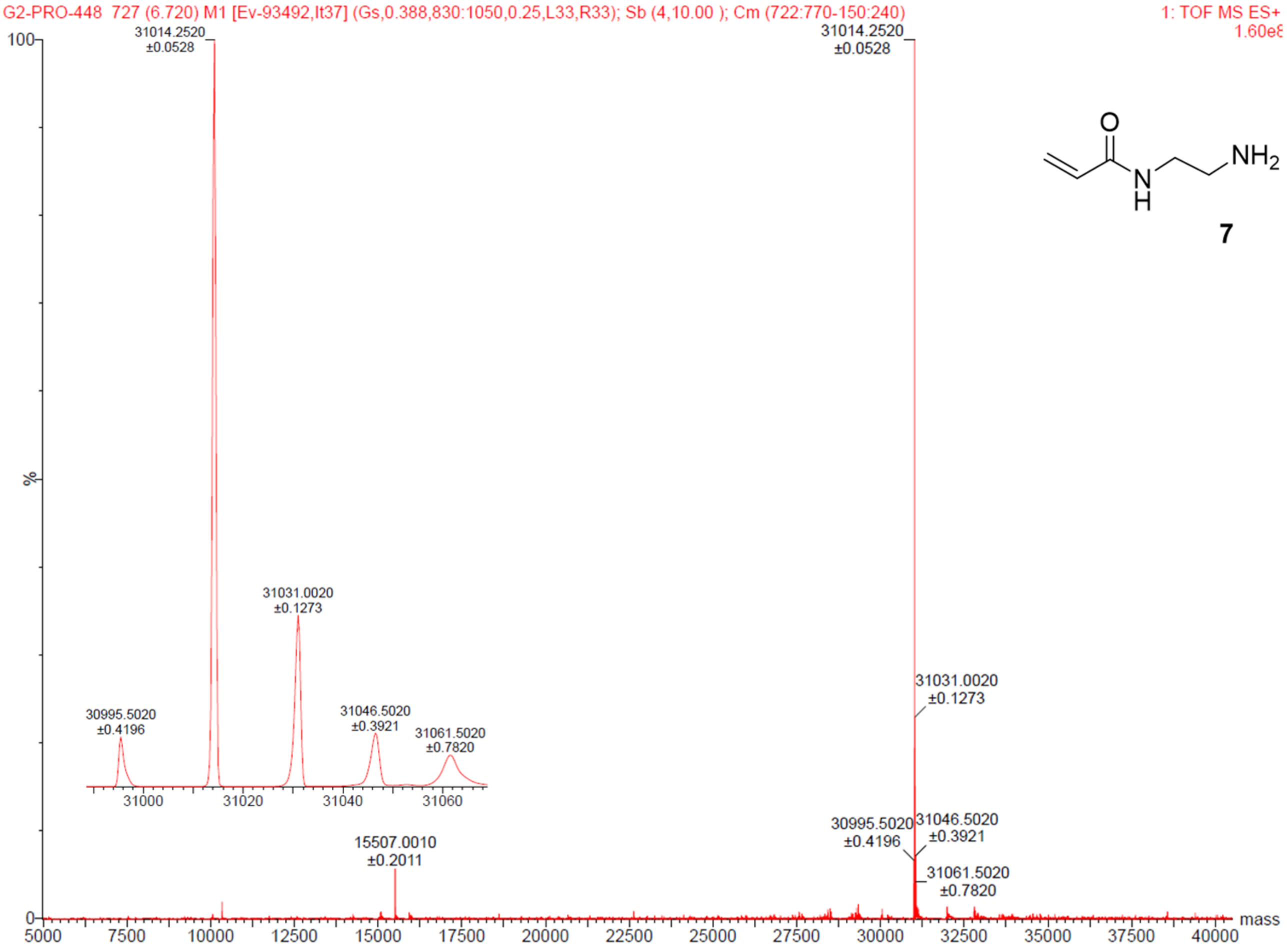

Figure S14. Deconvoluted spectrum of 14-3-3σ/ERα complex (1:1 molar ratio), after incubation with **7** (10 molar equivalents).

14-3-3σ + 8
Obs.: **8**, MW = 128.1 Da
Calculated Molecular Weight:
Protein: 31014.5
Adduct, protein + **8**: 31142.6

Observed Mass:
31016.3

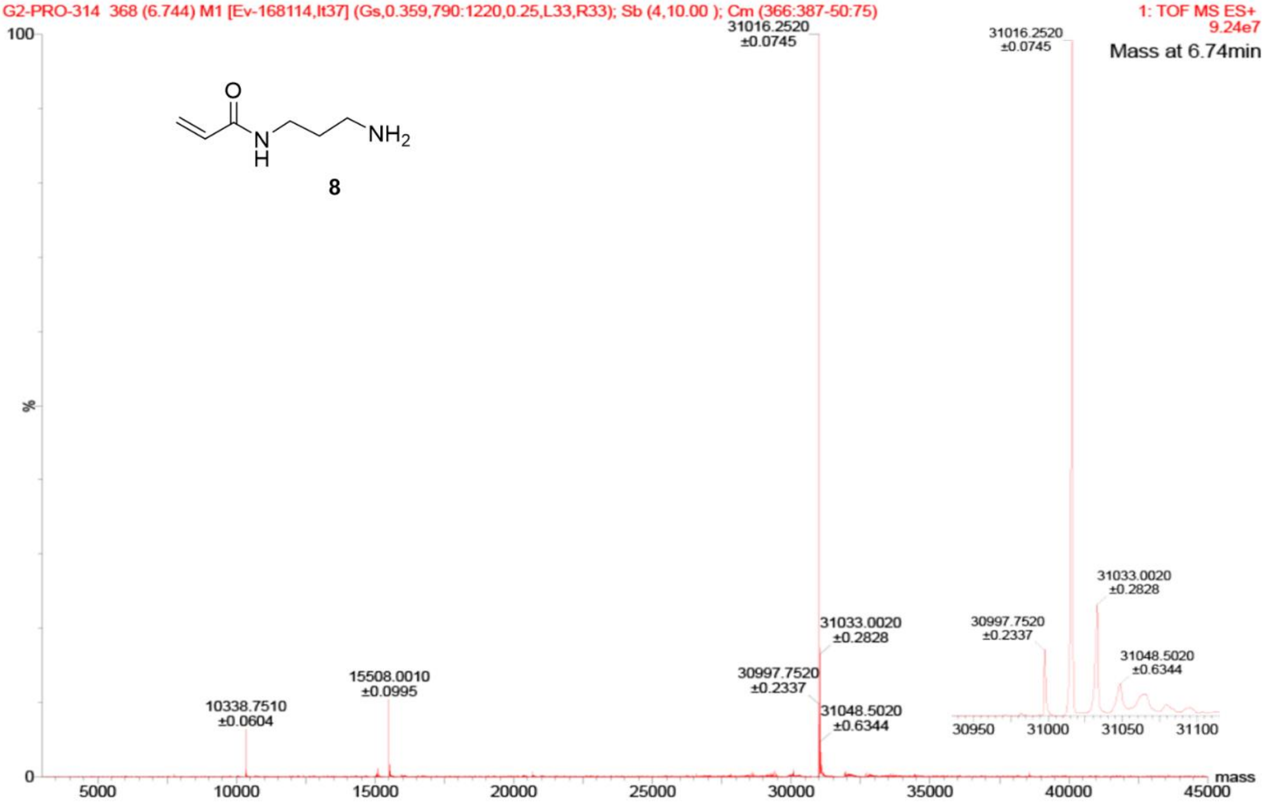

Figure S15. Deconvoluted spectrum of 14-3-3σ after incubation with **8** (1 molar equivalent, 24 h).

1.2. FP: control experiments and replicates of key experiments

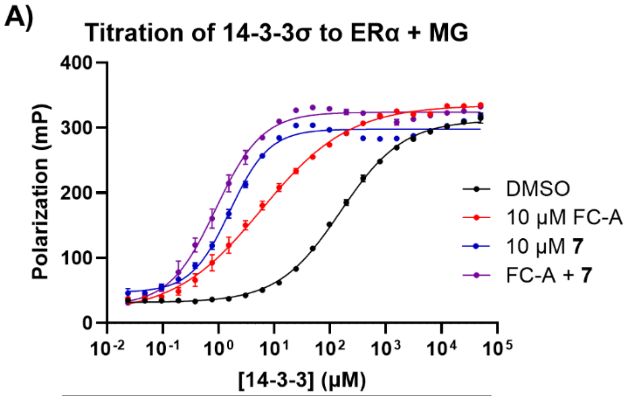

	DMSO	10 μM FC-A	10 μM **7**	FC-A + **7**
$K_{d\text{-}App}$ (nM)	154.8	5.946	1.606	0.8912

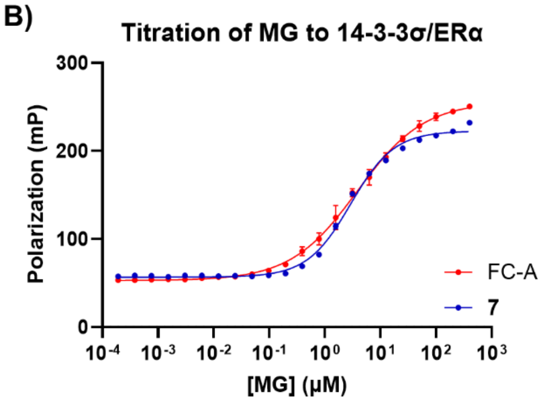

	**7**	FC-A
EC_{50} (μM)	2.896	3.818

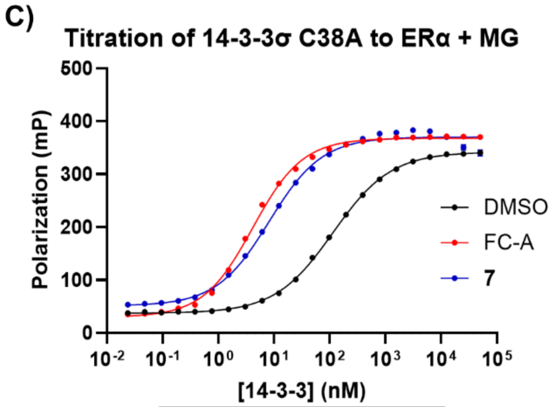

	DMSO	FC-A	**7**
$K_{d\text{-}App}$ (nM)	114.2	4.144	8.159

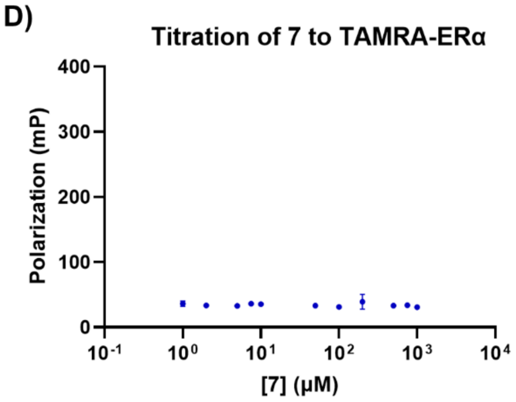

Fig S16. (A) Duplicate experiment, titration of 14-3-3σ to TAMRA-ERα and co-treatment of **7** and FC-A at 10 μM each. **(B)** Duplicate experiment, MG titration (**7** and FC-A) to 50 nM 14-3-3σ and 10 nM TAMRA-ERα. (**C**) Duplicate experiment: titration of 14-3-3σ C38A mutant to TAMRA-ERα in the presence of MGs. **(D)** Control experiment: titration of **7** to TAMRA-ERα.

A)

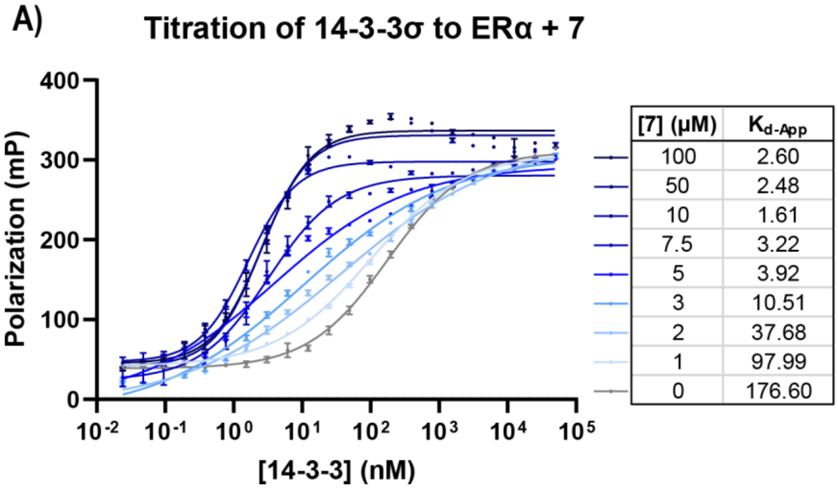

[7] (μM)	$K_{d\text{-}App}$
100	2.60
50	2.48
10	1.61
7.5	3.22
5	3.92
3	10.51
2	37.68
1	97.99
0	176.60

B)

[7] (μM)	Goodness of Fit (R squared)	
	Monophasic	Biphasic
100	0.9897	0.9972
50	0.9894	0.9994
10	0.9911	0.9921
7.5	0.9827	0.9946
5	0.9813	0.9995
3	0.9941	0.9992
2	0.9967	0.9994
1	0.9993	0.9996
0	0.9985	0.9991

Fig S17. (A) 2D protein 14-3-3σ titration to ERα in the presence of **7**: monophasic curve fitting and **(B)** comparison between goodness of fitness scores from monophasic and biphasic models.

1.3. Thermal stability data: melting curves and control experiments

Apart from control (apo) 14-3-3σ, all other experiments were conducted in the presence of 1% DMSO

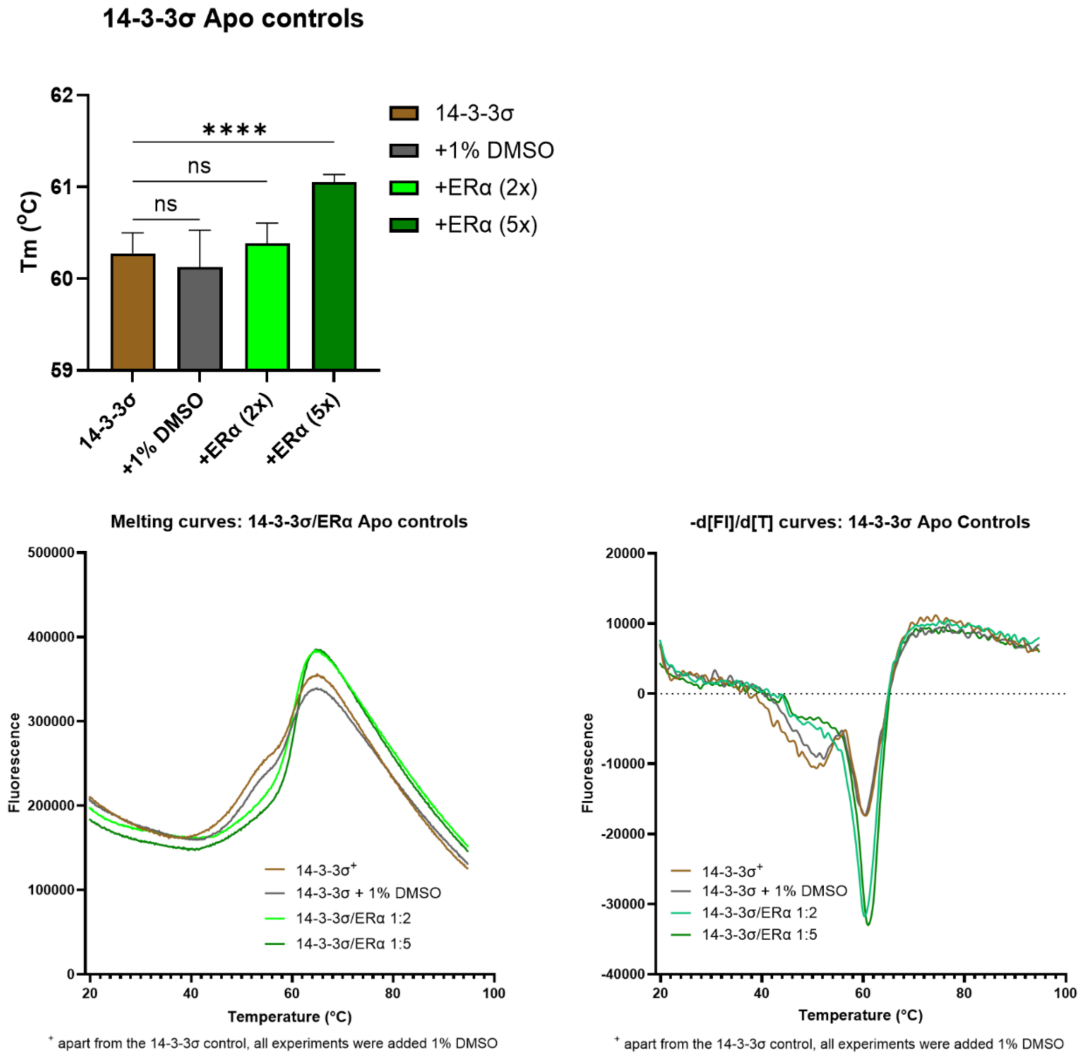

Figure S18. TSA experiments for apo protein and peptide controls, melting temperatures and curves (raw and derivative plots). Dunnett's test: ns = P>0.1; * = P<0.1, ** = P<0.01, *** = P<0.001, **** = P<0.0001.

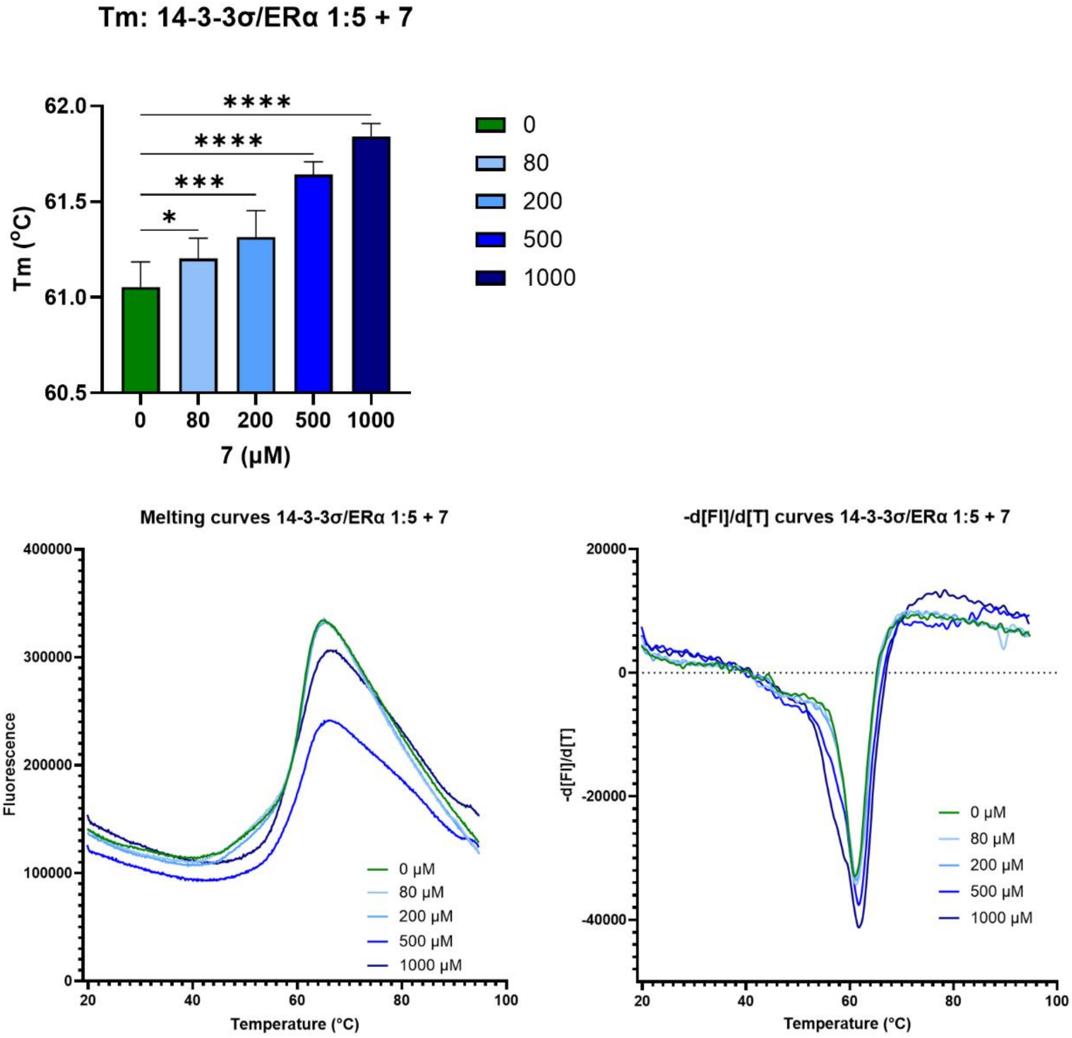

Figure S19. TSA experiments for 14-3-3σ/ERα 1:5 treated with variable concentration of **7**, melting temperatures and curves (raw and derivative plots). Dunnett's test: ns = P>0.1; * = P<0.1, ** = P<0.01, *** = P<0.001, **** = P<0.0001.

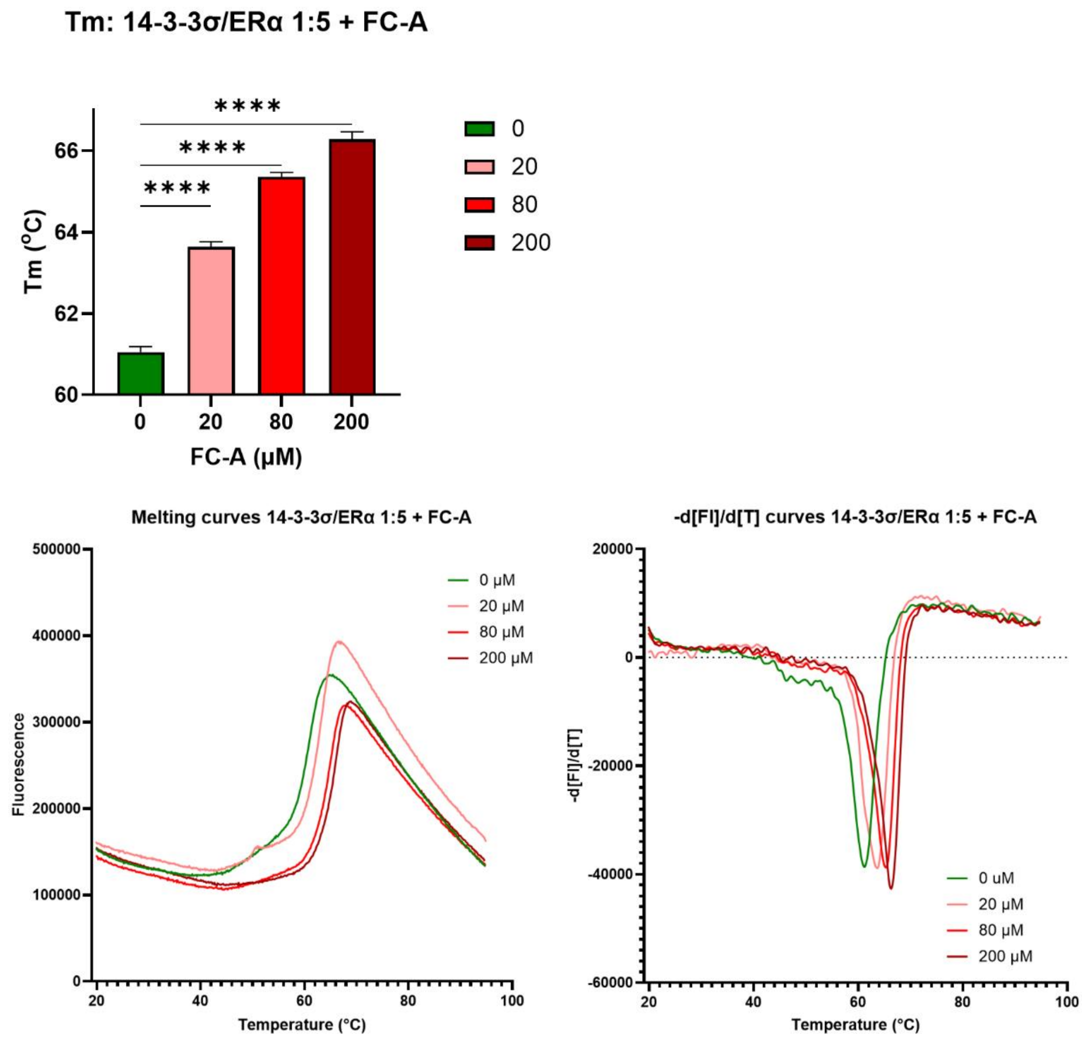

Figure S20. TSA experiments for 14-3-3σ/ERα 1:5 treated with variable concentration of **FC-A**, melting temperatures and curves (raw and derivative plots). Dunnett's test: ns = P>0.1; * = P<0.1, ** = P<0.01, *** = P<0.001, **** = P<0.0001.

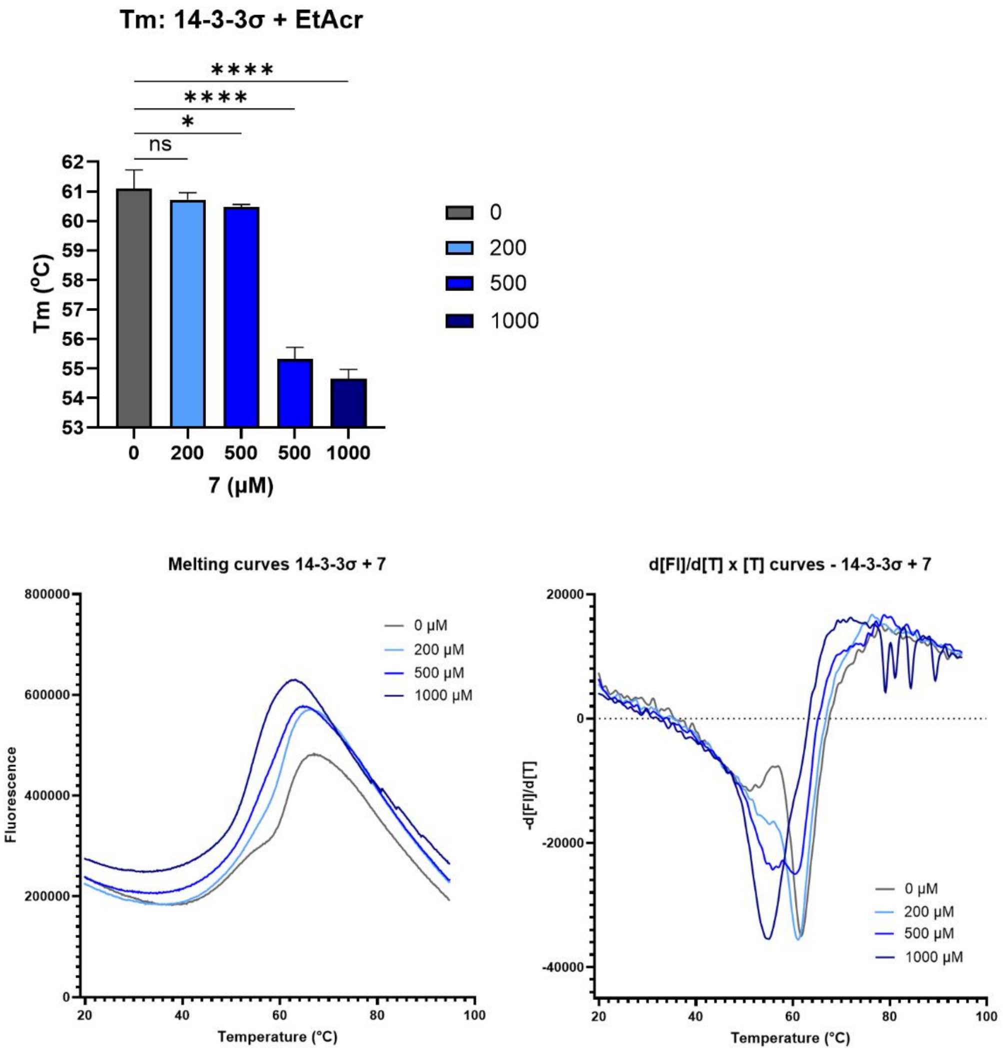

Figure S21. TSA experiments for 14-3-3σ treated with variable concentration of **7**, melting temperatures and curves (raw and derivative plots). Dunnett's test: ns = P>0.1; * = P<0.1, ** = P<0.01, *** = P<0.001, **** = P<0.0001.

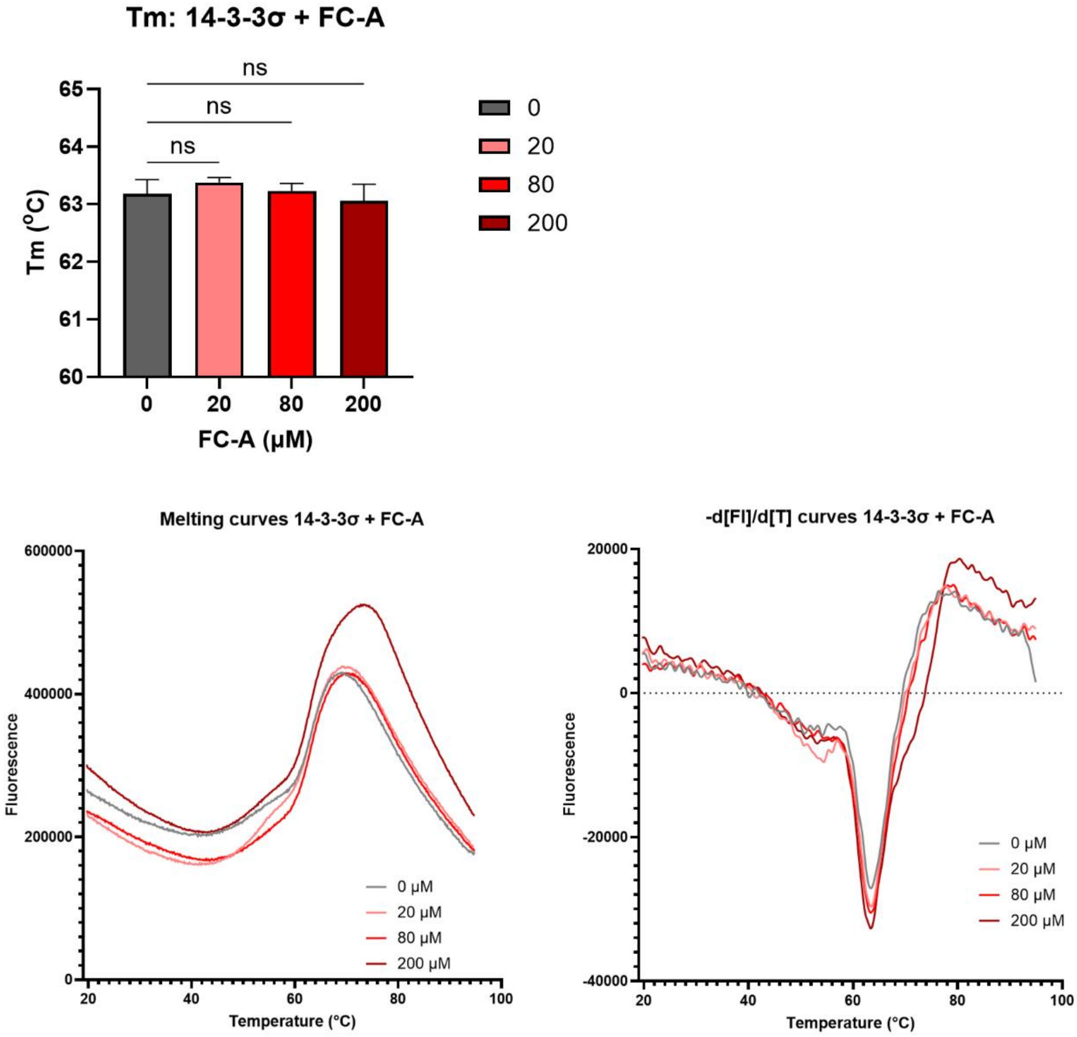

Figure S22. TSA experiments for 14-3-3σ treated with variable concentration of **FC-A**, melting temperatures and curves (raw and derivative plots). Dunnett's test: ns = P>0.1; * = P<0.1, ** = P<0.01, *** = P<0.001, **** = P<0.0001.

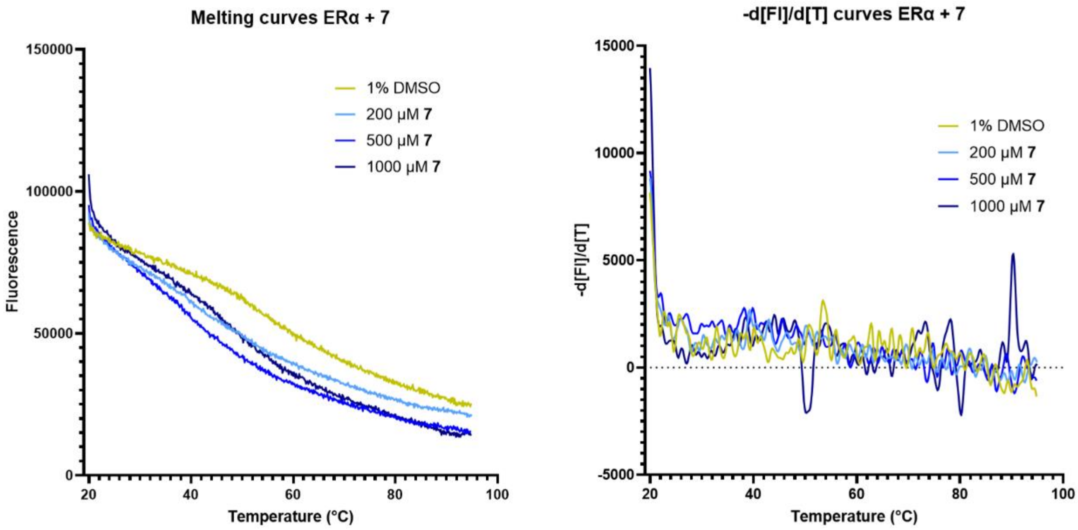

Figure S23. TSA control experiment: ERα 15-mer treated with variable concentration of **7,** fluorescence and derivative (-d[Fl]/d[T]) plots.

2. Chemistry Experimental

All the starting materials and reagents were obtained commercially unless otherwise stated. Glassware was dried in the oven at 150°C for 24 h before the reactions. Thin layer chromatography (TLC) was performed on silica gel G60 F254 (Merck) plates, and chromatograms were visualized by using UV light (λ = 254 and 365 nm) and ninhydrin staining. Column chromatography was performed using silica gel 60 (40−63 mm, 230−400 mesh). Bruker 400 MHz or Bruker 500MHz were used to collect the nuclear magnetic resonance spectra of hydrogen (^{1}H NMR) or carbon (^{13}C NMR). Chemical shifts are reported in parts per million (δ in ppm), coupling constants in hertz (J in Hz) and signal multiplicity as singlet (s), doublet (d), triplet (t), quartet (q) or multiplet (m). Broad signals are identified with (br). The samples were dissolved in deuterated chloroform (CDCl3), deuterated methanol (CD3OD) or deuterated dimethylsulfoxide (DMSO-d6) and the residual solvent signals were used as internal standard for correction: $CDCl_3$, δ 7.27 ppm (^{1}H NMR) and δ 77.16 ppm (^{13}C NMR); CD_3OD, δ 3.31 ppm (^{1}H NMR) and δ 49.00 ppm (^{13}C NMR); DMSO-d6 δ 2.50 ppm (^{1}H NMR) and δ 39.51 ppm (^{13}C NMR). Unless otherwise stated, the stock solutions for all compounds were prepared by dissolving 0.1 mmol of product in 1 mL dimethylsulfoxide (DMSO), to the final concentration of 100 mM. Further dilutions were made with the proper solvents for each experiment. Fusicoccin-A was kindly donated by Yuske Higuchi.[1]

2.1. Chemical synthesis

2.1.1. General method for amide, sulfonamide, or ester bond formation using acid chlorides or acid anhydrides

To a solution of nucleophile (1 mmol) in DCM (3 mL) was added triethylamine (3 mmol). With stirring, the solution was cooled to 0°C before a solution of the acylating agent (acid chloride or acid anhydride, 1.2 mmol) in 3 mL DCM was added dropwise over 1 minute. After 30 minutes, the reaction was allowed to reach room temperature, and was monitored using TLC. Unless otherwise stated, the reaction was transferred to a separation funnel along with 50 mL ethyl acetate (EtOAc) and washed with 10% Na_2CO_3 (aq) (3 x 50 mL), and brine (3 x 50 mL). The solution was dried over $MgSO_4$, filtered, and the solvent removed *in vacuo* to furnish the product which was purified using column chromatography according to the conditions outlined below.

2.1.1.1. tert-butyl (3-(2-chloro-5-nitrobenzamido)propyl)carbamate, ***1b***

Nucleophile: tert-butyl (2-aminopropyl)carbamate. Acylating reagent: 2-chloro-5-nitrobenzoyl chloride. Mobile phase for chromatography EtOAc : MeOH 9 : 1. White solid, m = 293 mg (94% yield). ^{1}H NMR (400 MHz, $CDCl_3$) δ ppm 8.46 (d, *J* = 2.7 Hz, 1H), 8.20 (dd, *J* = 8.8, 2.7 Hz, 1H), 7.59 (d, *J* = 8.8 Hz, 1H), 4.85 (s, 1H), 3.54 – 3.51 (m, 2H), 3.24-3.29 (m, 2H), 1.77 – 1.74 (m, 2H), 1.42 (s, 9H). ^{13}C NMR (100 MHz, $CDCl_3$) δ ppm 164.5, 157.0, 146.5, 137.7, 137.0, 131.3, 125.4, 124.9, 79.7, 37.0, 36.4, 30.2, 28.3.

2.1.1.2. tert-butyl (3-(2-iodoacetamido)propyl)carbamate, ***2b***

Nucleophile: tert-butyl (2-aminopropyl)carbamate. Acylating reagent: iodoacetyl chloride. Mobile phase for chromatography: DCM : EtOAc 3:2. White solid, m = 328 mg (96% yield).

^{1}H NMR (500 MHz, $CDCl_3$) δ ppm 4.93 (s, 1H), 4.03 (s, 1H), 3.68 (s, 1H), 3.37 - 3.27 (m, 2h), 3.18 - 3.16 (m, 2H), 1.68 - 1.60 (m, 2H), 1.43 (s, 9H). ^{13}C NMR (125 MHz, CDCl3) δ ppm 167.8, 166.5, 79.6, 42.7, 36.8, 30.0, 28.5, -0.4.

*2.1.1.3. tert-butyl (2-(vinylsulfonamido)ethyl)carbamate, **5b***

Nucleophile: tert-butyl (2-aminoethyl)carbamate. Acylating reagent: 2-chloroethanesulfonyl chloride. Mobile phase for chromatography: hexanes : EtOAc 1:1. White solid, m = 25 mg (10% yield). ^{1}H NMR (500 MHz, $CDCl_3$) δ ppm 6.50 (dd, J = 16.6, 9.9 Hz, 1H), 6.20 (d, J = 16.6 Hz, 1H), 5.91 (d, J = 9.9 Hz, 1H), 3.25 - 3.24 (m, 2H), 3.10 - 3.07 (m, 2H), 1.40 (s, 9H). ^{13}C NMR (125 MHz, $CDCl_3$) δ ppm 156.5, 135.7, 126.6, 79.7, 43.3, 40.4, 28.3.

*2.1.1.4. tert-butyl (3-(vinylsulfonamido)propyl)carbamate, **6b***

Nucleophile: tert-butyl (2-aminopropyl)carbamate. Acylating reagent: 2-chloroethanesulfonyl chloride. Mobile phase for chromatography: hexanes : EtOAc 1:1. White solid, m = 27 mg (10% yield). ^{1}H NMR (500 MHz, $CDCl_3$) δ ppm: 6.51 (dd, J = 16.6, 9.9 Hz, 1H), 6.20 (d, J =16.6 Hz, 1H), 5.89 (d, J = 9.9 Hz, 1H), 5.48 (s, 1H), 4.80 (s, 1H), 3.21 - 3.22 (m, 2H), 3.05-3.02 (m, 2H), 1.70 - 1.65 (m, 2H), 1.41 (s, 9H). ^{13}C NMR (125 MHz, $CDCl_3$) δ ppm 156.8, 136.2, 126.1, 79.6, 39.8, 36.9, 30.5, 28.3.

*2.1.1.5. tert-butyl (2-acrylamidoethyl)carbamate, **7b***

Nucleophile: tert-butyl (2-aminoethyl)carbamate. Acylating reagent: acryloyl chloride. Mobile phase for chromatography: hexanes : EtOAc 1:1. White solid, m = 202 mg (94% yield).
^{1}H NMR (400 MHz, $CDCl_3$) δ ppm: 6.72 (br s, 1H), 6.27 (dd, J = 17.0, 1.5 Hz, 1H), 6.27 (dd, J = 17.0, 1.4 Hz, 1H), 6.12 (dd, J = 17.0, 10.4 Hz, 1H), 5.63 (dd, J = 10.4, 1.4 Hz, 1H), 5.17 (br s, 1H), 3.44 (m, 2H), 3.31 (m, 2H), 1.49 (s, 9H). ^{13}C NMR (100 MHz, $CDCl_3$) δ ppm: 166.2, 157.1, 130.9, 126.2, 79.7, 40.9, 40.1, 28.3.

*2.1.1.6. tert-butyl (2-acrylamidopropyl)carbamate, **8b***

Nucleophile: tert-butyl (2-aminopropyl)carbamate. Acylating reagent: acryloyl chloride. Mobile phase for chromatography: hexanes : EtOAc 1:1. White solid, m = 157 mg (69% yield).
^{1}H NMR (400 MHz, $CDCl_3$) δ ppm: 6.55 (br s, 1H), 6.28 (d, J = 17.0 Hz, 1H), 6.14 (dd, J = 17.0, 10.0 Hz, 2H), 5.63 (d, J = 10 Hz, 1H), 4.97 (br s, 1H), 3.41 (m, 2H), 3.20 (m, 2H), 1.67 (m, 2H), 1.44 (s, 9H). ^{13}C NMR (100 MHz, $CDCl_3$) δ ppm: 165.9, 156.8, 131.1, 126.0, 79.4, 37.0, 35.8, 30.1, 28.3.

*2.1.1.7. tert-butyl (2-acrylamidobutyl)carbamate, **9b***

Nucleophile: tert-butyl (2-aminobutyl)carbamate. Acylating reagent: acryloyl chloride. Mobile phase for chromatography: hexanes : EtOAc 1:1. White solid, m = 152 mg (63% yield).
^{1}H NMR (400 MHz, $CDCl_3$) δ ppm: 6.28 (d, J = 17.0 Hz, 1H), 6.11 (m, 2H), 6.12 (d, J = 10.0 Hz, 1H), 5.63 (d, J = 10 Hz, 1H), 4.68 (br s, 1H), 3.36 (m, 2H), 3.13 (m, 2H), 1.56 (m, 2H), 1.44 (s, 9H). ^{13}C NMR (100 MHz, $CDCl_3$) δ ppm: 165.6, 156.2, 130.9, 126.1, 79.3, 40.0, 39.2, 28.3, 27.7, 26.4.

*2.1.1.8. tert-butyl 4-acryloylpiperazine-1-carboxylate, **10b***

Nucleophile: tert-butyl piperazine-1-carboxylate. Acylating reagent: acryloyl chloride. Mobile phase for chromatography: hexanes : EtOAc 1:1. White solid, m = 197 mg (82% yield).

^{1}H NMR (500 MHz, $CDCl_3$) δ ppm: 6.56 (dd, J = 16.7, 10.6 Hz, 1H), 6.31 (d, J = 16.6, 1H), 5.73 (d, J = 10.5 Hz, 1H), 3.66 (m, 2H), 3.54 (m, 2H), 3.45 (m, 4H), 1.48 (s, 9H). ^{13}C NMR (100 MHz, $CDCl_3$) δ ppm: 165.5, 154.5, 128.3, 127.2, 80.3, 45.5, 41.7, 28.3.

2.1.1.9. tert-butyl 4-acryloyl-1,4-diazepane-1-carboxylate, ***11b***

Nucleophile: tert-butyl 1,4-diazepane-1-carboxylate. Acylating reagent: acryloyl chloride. Mobile phase for chromatography: hexanes : EtOAc 1:1. White solid, m = 216 mg (85% yield).
^{1}H NMR (500 MHz, $CDCl_3$) δ ppm: 6.57 (m, 1H), 6.33 (m, 1H), 5.70 (m, 1H), 3.63 (m, 3H), 3.52 (m, 3H), 3.41 (m, 1H), 3.35 (m, 1H), 1.88 (m, 2H), 1.45 (m, 9H). ^{13}C NMR (125 MHz, $CDCl_3$) δ ppm: 166.0, 155.0, 127.9, 127.5, 79.9, 48.5, 47.6, 47.1, 45.6, 28.3, 27.4. Multiple sets of signals were observed for several carbons due to the presence of slowly interconverting conformers at room temperature, the most abundant signals were reported for each carbon.

2.1.1.10. tert-butyl ((1R,2R)-2-acrylamidocyclohexyl)carbamate, ***12b***

Nucleophile: tert-butyl ((1R,2R)-2-aminocyclohexyl)carbamate. Acylating reagent: acryloyl chloride. Mobile phase for chromatography: hexanes : EtOAc 1:1. White solid, m = 247 mg (92% yield).
^{1}H NMR (400 MHz, $CDCl_3$) δ ppm: : 6.46 (br s, 1H), 6.22 (d, J = 17.0 Hz, 1H), 6.04 (dd, J = 17.0, 10.3 Hz, 1H), 5.58 (d, J = 10.3 Hz, 1H), 4.72 (d, J = 8.1 Hz, 1H), 3.71 – 3.55 (m, 1H), 3.48 – 3.33 (m, 1H), 2.19 – 2.06 (m, 2H), 2.05 – 1.91 (m, 1H), 1.82 – 1.65 (m, 2H), 1.38 (s, 9H), 1.30 – 1.15 (m, 4H). ^{13}C NMR (100 MHz, $CDCl_3$) δ ppm: 165.6, 157.0, 131.3, 125.7, 79.6, 55.5, 53.6, 32.4, 28.3, 25.0, 24.4.

2.1.1.11. tert-butyl ((1S,2S)-2-acrylamidocyclohexyl)carbamate, ***13b***

Nucleophile: tert-butyl ((1S,2S)-2-aminocyclohexyl)carbamate. Acylating reagent: acryloyl chloride. Mobile phase for chromatography: hexanes : EtOAc 1:1. White solid, m = 228 mg (85% yield).
^{1}H NMR (400 MHz, $CDCl_3$) δ ppm: ^{1}H NMR (400 MHz, $CDCl_3$) δ ppm: 6.46 (br d, J = 6.6 Hz, 1H), 6.22 (dd, J = 17.0, 1.1 1H), 6.04 (dd, J = 17.0, 10.4 Hz, 1H), 5.58 (dd, J = 10.4, 1.1 Hz, 1H), 4.71 (d, J = 8.1 Hz, 1H), 3.76 – 3.53 (m, 1H), 3.51 – 3.31 (m, 1H), 2.22 – 1.90 (m, 2H), 1.85 – 1.66 (m, 1H), 1.38 (s, 9H), 1.32 – 1.16 (m, 4H). ^{13}C NMR (100 MHz, $CDCl_3$) δ ppm: 165.6, 157.0, 131.3, 125.8, 79.7, 55.6, 53.6, 32.4, 28.3, 25.1, 24.5.

2.1.1.12. tert-butyl (2-propionamidoethyl)carbamate*,* ***14b***

Nucleophile: tert-butyl (2-aminoethyl)carbamate. Acylating reagent: propionic anhydride. Mobile phase for chromatography: hexanes : EtOAc 1:1. White solid, m = 182 mg (84% yield).
^{1}H NMR (400 MHz, $CDCl_3$) δ ppm: 6.60 (br s, 1H), 5.32 (br s, 1H), 3.32 (m, 2H), 3.21 (m, 2H), 2.17 (q, J = 7.63 Hz, 2H), 1.39 (s, 9H), 1.11 (t, J = 7.63 Hz, 3H). ^{13}C NMR (100 MHz, $CDCl_3$) δ ppm: 174.6, 156.9, 79.4, 40.5, 40.1, 29.5, 28.2, 9.7.

2.1.1.13. tert-butyl (2-cinnamamidoethyl)carbamate*,* ***15b***

Nucleophile: tert-butyl (2-aminoethyl)carbamate. Acylating reagent: cinnamoyl chloride. Mobile phase for chromatography: hexanes : EtOAc 1:1. White solid, m = 228 mg (85% yield).
^{1}H NMR (400 MHz, $CDCl_3$) δ ppm: 7.60 (d, J = 15.6 Hz, 1H), 7.38 (m, 5H), 6.75 (br s, 1H), 6.44 (d, J = 15.6 Hz, 1H), 5.22 (br s, 1H), 3.53 (m, 2H), 3.32 (m, 2H), 1.44 (s, 9H). ^{13}C NMR (100 MHz, $CDCl_3$) δ ppm: 166.6, 157.1, 140.8, 134.8, 129.6, 128.7, 127.7, 120.7, 79.7, 40.9, 40.2, 28.3.

2.1.1.14. 2-((tert-butoxycarbonyl)amino)ethyl acrylate, ***16b***

Nucleophile: tert-butyl (2-hydroxyethyl)carbamate. Acylating reagent: acryloyl chloride. Mobile phase for chromatography: hexanes : EtOAc 1:1. White solid, m = 151 mg (70% yield).

^{1}H NMR (400 MHz, $CDCl_3$) δ ppm: 6.43 (dd, *J* = 17.3, 1.1 Hz, 1H), 6.33 (dd, *J* = 17.3, 10.4 Hz, 1H), 5.86 (dd, *J* = 10.4, 1.1 Hz, 1H), 4.83 (br s, 1H), 4.22 (t, *J* = 5.2 Hz, 2H), 3.42 (m, 2H), 1.45 (s, 9H). ^{13}C NMR (100 MHz, $CDCl_3$) δ ppm: 166.1, 155.8, 131.3, 128.1, 79.6, 63.8, 39.7, 28.4.

2.1.1.15. N-(2-(piperidin-1-yl)ethyl)acrylamide, ***15***

Nucleophile: 2-(piperidin-1-yl)ethan-1-amine. Acylating reagent: acryloyl chloride. Mobile phase for chromatography: EtOAc : methanol : 9 : 1. Pink solid, m = 181 mg (99% yield).

^{1}H NMR (500 MHz, $CDCl_3$) δ ppm: 6.32 (br s, 1H), 6.27 (dd, *J* = 17.0, 1.5 Hz, 1H), 6.13 (dd, *J* = 17.0, 10.2 Hz, 1H), 6.27 (dd, *J* = 10.2, 1.5 Hz, 1H), 3.41 (m, 2H), 2.47 (t, *J* = 6.15 Hz, 2H), 2.37 (m, 4H), 1.57 (m, 4H), 1.45 (m, 2H). ^{13}C NMR (125 MHz, $CDCl_3$) δ ppm: 165.5, 131.1, 125.9, 57.0, 54.2, 36.0, 25.9, 24.3. *m/z* (ESI): calcd. for $C_{10}H_{19}N_2O$: 183.1492; found: 183.1498 $[M+H]^+$.

2.1.1.16. N-(2-hydroxyethyl)acrylamide, ***16***

Nucleophile: ethanolamine (2.5 mmol). Acylating reagent: acryloyl chloride (1 mmol). This reaction did not go through liquid extraction before the column chromatography. Mobile phase for chromatography: EtOAc : methanol 4:1. Colorless oil, m = 69 mg (60% yield).

^{1}H NMR (400 MHz, CD_3OD) δ ppm: 6.23 (m, 2H), 5.65 (m, 1H), 3.63 (t, J = 5.7 Hz, 2H), 3.38 (t, J = 5.6 Hz, 2H). ^{13}C NMR (100 MHz, CD_3OD) δ ppm: 168.5, 132.2, 126.8, 61.6, 43.1. *m/z* (ESI): calcd. for $C_5H_{10}NO_2$: 116.0706; found: 116.0714 $[M+H]^+$.

2.1.1.17. acryloylglycine, ***17***

Nucleophile: glycine (1.2 mmol). Acylating reagent: acryloyl chloride (1 mmol). This reaction did not go through liquid extraction before the column chromatography. Mobile phase for chromatography: EtOAc : methanol : acetic acid 88 : 10 : 2. , m = 16 mg (12% yield).

^{1}H NMR (400 MHz, CD_3OD) δ ppm: 6.32 (dd, J = 17.2, 9.9 Hz, 1H), 6.24 (dd, J = 17.2, 1.8 Hz, 1H), 5.79 (dd, J = 9.9, 1.83 Hz, 1H), 4.02 (s, 2H). ^{13}C NMR (100 MHz, CD_3OD) δ ppm: 172.8, 168.2, 129.7, 127.5, 41.0.): *m/z* (ESI): calcd. for $C_5H_6NO_3$: 128.0353; found: 128.0348 $[M-H]^-$.

2.1.1.18. N-propylacrylamide, ***18***

Nucleophile: propylamine. Acylating reagent: acryloyl chloride. Mobile phase for chromatography: hexanes : EtOAc 1:1. Colorless oil, m = 104 mg (92% yield).

^{1}H NMR (400 MHz, $CDCl_3$) δ ppm: 6.25 (dd, *J* = 17.0, 1.6 Hz, 1H), 6.12 (m, 2H), 5.60 (dd, *J* = 10.1, 1.6 Hz, 1H), 3.27 (m, 2H), 1.55 (m, 2H), 0.92 (t, *J* = 7.43 Hz, 3H). ^{13}C NMR (100 MHz, $CDCl_3$) δ ppm: 165.6, 131.0, 125.9, 41.2, 22.7, 11.3. *m/z* (ESI): calcd. for $C_6H_{12}NO$: 114.0913; found: 114.0919 $[M+H]^+$.

2.1.2. General method for maleimide synthesis

To a solution of maleic anhydride (2 mmol) in anhydrous toluene (15 mL) was added the amine (3 mmol). The reaction mixture was stirred under N_2 at room temperature for 4 h. The solvent was removed *in vacuo*, before sodium acetate (2.5 mmol) and acetic anhydride (2.5 mL) were added.

The reaction was stirred at 65°C for 5 h under N_2. The acetic anhydride was removed *in vacuo* and the product was purified using column chromatography (EtOAc : hexanes, 7 : 13).

2.1.2.1. tert-butyl (2-(2,5-dioxo-2,5-dihydro-1H-pyrrol-1-yl)ethyl)carbamate, ***3b***

White solid, m = 192 mg (43% yield).
^{1}H NMR (500 MHz, CD_3OD) δ ppm: δ ppm 6.70 (s, 2H), 4.75 (s, 1H), 3.66 - 3.64 (m, 2), 3.30 - 3.34 (m, 2H), 1.39 (s, 9H). ^{13}C NMR (125 MHz, CD_3OD) δ ppm: 170.9, 156.0, 134.3 (C-6,7), 79.6 (C-12), 39.5 (C-2), 38.1 (C-3), 28.4 (C-13-15).

2.1.2.2. tert-butyl (3-(2,5-dioxo-2,5-dihydro-1H-pyrrol-1-yl)propyl)carbamate, ***4b***

White solid, m = 285 mg (50% yield).
^{1}H NMR (500 MHz, CD_3OD) δ ppm: ppm 6.69 (s, 2H), 3.57 (t, *J* = 5.0 Hz, 2H), 3.09-3.05 (m, 2H), 1.77 -1.72 (m, 2H), 1.43 (s, 9H). ^{13}C NMR (125 MHz, CD_3OD) δ ppm: 170.9, 155.8, 134.1, 79.2, 37.3, 34.9, 28.8, 28.4.

2.1.3. Synthesis of N-(2-(2,3-ditertbutoxycarbonylguanidino)ethyl)acrylamide, ***14b***

To a cooled solution (0°C) of acrylamide **7** (0.5 mmol) and triethylamine (1.5 mmol) in DCM (1 mL), a solution of *N,N'*-Di-Boc-1H-pyrazole-1-carboxamidine (0.55 mmol) in DCM (1 mL) was added dropwise over 1 minute under stirring. The reaction was allowed to reach room temperature, and stirred overnight. The solvent was removed *in vacuo* and the product was purified using column chromatography (mobile phase Hexanes : EtOAc 1 : 1) to yield a white solid (m = 189 mg, 99% yield).
^{1}H NMR (500 MHz, $CDCl_3$) δ ppm: 11.41 (br s, 1H), 8.68 (m, 1H), 8.11 (br s, 1H), 7.62 (d, *J* = 2.0 Hz, 1H), 6.26 (dd, *J* = 17.2, 1.6 Hz, 1H), 6.16 (dd, *J* = 17.2, 10.2 Hz, 1H), 5.58 (dd, *J* = 10.2, 1.6 Hz, 1H), 3.59 (m, 2H), 3.49 (m, 2H), 1.51 (s, 9H), 1.51 (s, 9H). ^{13}C NMR (125 MHz, CD_3OD) δ ppm: 165.8, 162.7, 157.6, 153.0, 131.4, 125.4, 83.7, 79.6, 41.8, 40.2, 28.2, 28.0.

2.1.4. General method for removal of terbulyloxycarbonyl (Boc) protecting group

TFA (1 mL) was added to a solution of the relevant Boc-protected intermediate (0.1 mmol) in DCM (1 mL). The reaction mixture was stirred for 1 h at room temperature. The solvents were removed *in vacuo* to yield the pure products in quantitative yields.

2.1.4.1. N-(3-aminopropyl)-2-chloro-5-nitrobenzamide, ***1***

^{1}H NMR (400 MHz, CD_3OD) δ ppm 8.30-8.26 (m, 2H), 7.73 (d, *J* = 8.7 Hz, 1H), 3.50 - 3.47 (m, 2H), 3.31 (s, 1H), 3.06 - 3.02 (m, 2H), 2.00 - 1.93 (m, 2H). ^{13}C NMR (100 MHz, CD_3OD) δ 166.9, 146.5, 137.3, 137.0, 131.1, 125.4, 123.4, 37.1, 36.2, 27.3. *m/z* (ESI): calcd. for $C_{10}H_{13}ClN_3O_3$: 258.0640; found: 258.0636 [M+H]+.

2.1.4.2. N-(3-aminopropyl)-2-iodoacetamide, ***2***

^{1}H NMR (400 MHz, CD_3OD) δ ppm 3.8 (s, 1H), 3.53 (s, 2H), 3.13 - 3.10 (m, 2H), 2.81 - 2.77 (m, 2H), 1.73 - 1.65 (m, 2H). ^{13}C NMR (100 MHz, CD_3OD) δ ppm 170.9, 36.7, 36.0, 27.1, -3.7. *m/z* (ESI): calcd. for $C_5H_{12}IN_2O$: 242.9989; found: 242.9997 $[M+H]^+$.

*2.1.4.3. 1-(2-aminoethyl)-1H-pyrrole-2,5-dione, **3***

^{1}H NMR (500 MHz, CD_3OD) δ ppm 6.78 (s, 2H), 3.74 - 3.71 (m, 2H), 3.08 (t, *J* = 5.7 Hz, 2H). ^{13}C NMR (125 MHz, CD_3OD) δ ppm 170.9 (C-6/8), 134.3 (C-4/5), 38.4 (C-1), 34.8 (C-2). *m/z* (ESI): calcd. for $C_6H_9N_2O_2$: 141.0664; found: 141.0664 $[M+H]^+$.

*2.1.4.4. 1-(3-aminopropyl)-1H-pyrrole-2,5-dione, **4***

^{1}H NMR (500 MHz, CD_3OD) δ ppm 6.84 (s, 2H), 3.60 (t, *J* = 6.6 Hz, 2H), 2.97 - 2.91 (m, 2H), 1.94 - 1.89 (m, 2H). ^{13}C NMR (125 MHz, CD_3OD) δ ppm 172.5, 135.5, 38.4, 35.4, 27.9. *m/z* (ESI): calcd. for $C_7H_{11}N_2O_2$: 155.0820; found: 155.0821 $[M+H]^+$.

*2.1.4.5. N-(2-aminoethyl)ethenesulfonamide, **5***

^{1}H NMR (500 MHz, CD_3OD) δ ppm 6.69 (dd, *J* = 16.6, 10.0 Hz, 1H), 6.22 (d, *J* = 16.6 Hz, 1H), 6.07 (d, *J* = 10.0 Hz, 1H), 3.24 (t, *J* = 5.9 Hz, 2H), 3.10 (t, *J* = 5.9 Hz, 2H). ^{13}C NMR (125 MHz, CD_3OD) δ ppm 135.3, 126.5, 39.8, 39.4. *m/z* (ESI): calcd. for $C_4H_{11}N_2O_2S$: 151.0536; found: 151.0541 $[M+H]^+$.

*2.1.4.6. N-(3-aminopropyl)ethenesulfonamide, **6***

^{1}H NMR (500 MHz, CD_3OD) δ ppm 6.63 (dd, *J* = 16.6, 10.0 Hz, 1H), 6.13 (d, *J* = 16.6 Hz, 1H), 5.96 (d, *J*= 10.0 Hz, 1H), 3.29 - 3.27 (m, 2H), 3.03 - 2.98 (m, 2H), 1.88 - 1.82 (m, 2H). ^{13}C NMR (125 MHz, CD_3OD) δ ppm 137.3, 126.9, 40.7, 38.2, 29.1. *m/z* (ESI): calcd. for $C_5H_{13}N_2O_2S$: 165.0692; found: 165.0690 $[M+H]^+$.

*2.1.4.7. N-(2-aminoethyl)acrylamide, **7***

^{1}H NMR (400 MHz, CD_3OD) δ ppm 6.27 – 6.25 (m, 2H), 5.73 – 5.70 (m, 1H), 3.53 (t, *J* = 6.0 Hz, 2H), 3.09 (t, *J* = 6.0 Hz, 2H). ^{13}C NMR (100 MHz, CD_3OD) δ ppm 167.9, 130.2, 126.1, 39.4, 36.9. *m/z* (ESI): calcd. for $C_5H_{11}N_2O$: 115.0871; found: 115.0873 $[M+H]^+$.

*2.1.4.8. N-(3-aminopropyl)acrylamide, **8***

^{1}H NMR (400 MHz, CD_3OD) δ ppm 6.25 – 6.24 (m, 2H), 5.71 – 5.68 (m, 1H), 3.38 – 3.35 (m, 2H), 2.96 – 2.93 (m, 2H), 1.91 – 1.84 (m, 2H). ^{13}C NMR (100 MHz, CD_3OD) δ ppm 167.5, 130.2, 125.9, 36.8, 35.5, 27.4. MS (ESI+) m/z: calcd. for $C_6H_{13}N_2O$: 129.1028; found 129.1027 [M+H.

*2.1.4.9. N-(4-aminobutyl)acrylamide, **9***

^{1}H NMR (400 MHz, CD_3OD) δ ppm 6.27 – 6.18 (m, 2H), 5.66 – 5.64 (m, 1H), 3.31 – 3.28 (m, 2H), 2.97 – 2.94 (m, 2H), 1.72 – 1.59 (m, 4H). ^{13}C NMR (100 MHz, CD_3OD) δ ppm 168.5, 132.1, 126.8, 40.5, 39.6, 27.5, 26.0. *m/z* (ESI): calcd. for $C_7H_{15}N_2O$: 143.1184; found: 143.1186 $[M+H]^+$.

2.1.4.10. 1-(piperazin-1-yl)prop-2-en-1-one, ***10***

^{1}H NMR (400 MHz, CD_3OD) δ ppm 6.77 (dd, *J* = 16.8, 10.6 Hz, 1H), 6.27 (dd, *J* = 16.8, 1.8 Hz, 1H), 5.82 (dd, *J* = 10.6, 1.8 Hz, 1H), 3.90 – 3.88 (m, 4H), 3.28 – 3.25 (m, 4H). ^{13}C NMR (100 MHz, CD_3OD) δ ppm 167.44, 130.07, 127.68, 44.30, 39.68. *m/z* (ESI): calcd. for $C_7H_{13}N_2O$: 141.1029; found: 141.1028 $[M+H]^+$.

2.1.4.11. 1-(1,4-diazepan-1-yl)prop-2-en-1-one*, **11***

^{1}H NMR (400 MHz, CD_3OD) δ ppm 6.76 (dd, *J* = 16.7, 10.6 Hz, 1H), 6.32 – 6.25 (m, 1H), 5.85 – 5.80 (m, 1H), 3.98 – 3.88 (m, 2H), 3.81 – 3.75 (m, 2H), 3.42 – 3.30 (m, 4H), 2.19 – 2.08 (m, 2H). ^{13}C NMR (100 MHz, CD_3OD) δ ppm 167.5, 128.1, 127.3, 46.2, 45.4, 41.7, 25.8 (obs.: Multiple sets of signals were observed for several carbons due to the presence of slowly interconverting conformers at room temperature, the most abundant signals were reported for each carbon). *m/z* (ESI): calcd. for $C_8H_{15}N_2O$: 155.1184; found: 155.1186 $[M+H]^+$.

2.1.4.12. N-((1*R*,2*R*)-2-aminocyclohexyl)acrylamide*, **12***

^{1}H NMR (500 MHz, CD_3OD) δ ppm 6.31 – 6.26 (m, *2*H), 5.70 (dd, J = 8.0, 4.0 Hz, 1H), 3.86 (td, *J* = 11.0 (x2), 4.2, 1H), 3.01 (td, *J* = 11.2 (x2), 4.1, 1H), 2.14 – 2.07 (m, 1H), 1.98 – 1.90 (m, 1H), 1.89 – 1.77 (m, 2H), 1.54 – 1.33 (m, 4H). ^{13}C NMR (125 MHz, CD_3OD) δ ppm 168.8, 132.1, 127.5, 56.3, 52.4, 32.6, 31.2, 25.6, 25.0. *m/z* (ESI): calcd. for $C_9H_{17}N_2O$: 169.1341; found: 169.1344 $[M+H]^+$.

2.1.4.13. N-((1*S*,2*S*)-2-aminocyclohexyl)acrylamide*, **13***

^{1}H NMR (500 MHz, CD_3OD) δ ppm 6.31 – 6.21 (m, *2*H), 5.71 (dd, J = 7.6, 4.3 Hz, 1H), 3.92 – 3.79 (m, 1H), 3.00 (td, *J* = 11.2 (x2), 3.8, 1H), 2.15 – 2.05 (m, 1H), 1.98 – 1.91 (m, 1H), 1.91 – 1.76 (m, 2H), 1.54 – 1.35 (m, 4H). ^{13}C NMR (125 MHz, CD_3OD) δ ppm 168.8, 132.1, 127.5, 56.3, 52.4, 32.6, 31.2, 25.6, 25.0. *m/z* (ESI): calcd. for $C_9H_{17}N_2O$: 169.1341; found: 169.1344 $[M+H]^+$.

2.1.4.14. N-(2-guanidinoethyl)acrylamide*, **14***

^{1}H NMR (400 MHz, CD_3OD) δ ppm 6.30 – 6.20 (m, 2H), 5.75 – 5.65 (m, 1H), 3.47 – 3.38 (m, 2H), 3.37 – 3.32 (m, 2H). ^{13}C NMR (100 MHz, CD_3OD) δ ppm 169.1, 159.1, 131.8, 127.4, 42.0, 39.6. *m/z* (ESI): calcd. for $C_6H_{13}N_4O$: 157.1089; found: 157.1094$[M+H]^+$.

2.1.4.15. 2-aminoethyl acrylate*, **19***

^{1}H NMR (400 MHz, CD_3OD) δ ppm 6.48 (dd, *J* = 17.3, 1.5 Hz, 1H), 6.23 (dd, *J* = 17.3, 10.4 Hz, 1H), 5.95 (dd, *J* = 10.4, 1.5 Hz, 1H), 4.41 – 4.36 (m, 2H), 3.30 – 3.26 (m, 2H). ^{13}C NMR (100 MHz, CD_3OD) δ ppm 165.7, 131.2, 127.4, 60.5, 38.4. *m/z* (ESI): calcd. for $C_5H_{10}NO_2$: 116.0712; found: 116.0722 $[M+H]^+$.

2.1.4.16. N-(2-aminoethyl)cinnamamide*, **20***

^{1}H NMR (400 MHz, CD_3OD) δ ppm 7.61 – 7.53 (m, 3H), 7.44 – 7.34 (m, 3H), 6.64 (d, J = 15.9 Hz, 2H), 3.59 (t, *J* = 5.9 Hz, 2H), 3.13 (t, *J* = 5.9 Hz, 2H). ^{13}C NMR (100 MHz, CD_3OD) δ ppm 170.0, 142.68, 136.2, 131.2, 130.1, 129.1, 121.3, 41.1, 38.6. *m/z* (ESI): calcd. for $C_{11}H_{15}N_2O$: 191.1184; found: 191.1181 $[M+H]^+$.

2.1.4.17. N-(2-aminoethyl)propionamide*, **21***

^{1}H NMR (400 MHz, CD_3OD) δ ppm 3.45 (t, *J* = 6.0 Hz, *2*H), 3.05 (t, J = 6.0 Hz, 2H), 2.25 (q, *J* = 7.6 Hz, 2H), 1.13 (t, *J* = 7.6 Hz, 3H). ^{13}C NMR (100 MHz, CD_3OD) δ ppm 176.9, 39.5, 36.8, 28.5, 8.6. *m/z* (ESI): calcd. for $C_5H_{13}N_2O$: 117.1022; found: 117.1005 $[M+H]^+$.

3. **Protein and peptide information**

3.1. Protein Sequence Information

Plasmids were obtained from commercial sources unless otherwise stated.

<u>14-3-3σ (UniProt: P31947, pPROEX Htb plasmid)</u>

*MSYYHHHHHHDYDIPTTENLYFQGAMGS*MERASLIQKAKLAEQAERYEDMAAFMKGAVEKGEELSCEERNLLS
VAYKNVVGGQRAAWRVLSSIEQKSNEEGSEEKGPEVREYREKVETELQGVCDTVLGLLDSHLIKEAGDAESRVFY
LKMKGDYYRYLAEVATGDDKKRIIDSARSAYQEAMDISKKEMPPTNPIRLGLALNFSVFHYEIANSPEEAISLAKTT
FDEAMADLHTLSEDSYKDSTLIMQLLRDNLTLWTADNAGEEGGEAPQEPQS

<u>14-3-3σ C38A (pPROEX Htb plasmid)</u>

*MSYYHHHHHHDYDIPTTENLYFQGA*MGSMERASLIQKAKLAEQAERYEDMAAFMKGAVEKGEELS**A**EERNLL
SVAYKNVVGGQRAAWRVLSSIEQKSNEEGSEEKGPEVREYREKVETELQGVCDTVLGLLDSHLIKEAGDAESRVF
YLKMKGDYYRYLAEVATGDDKKRIIDSARSAYQEAMDISKKEMPPTNPIRLGLALNFSVFHYEIANSPEEAISLAKT
TFDEAMADLHTLSEDSYKDSTLIMQLLRDNLTLWTADNAGEEGGEAPQEPQS

A plasmid containing a C38A mutation was generated by quick change mutagenesis using a QuikChange II Site-Directed Mutagenesis Kit (Agilent) using synthetic primers (Eurofins) and according to the manufacturer instructions. The purified plasmid containing the desired mutation was transformed into XL1-Blue supercompetent cells and the DNA obtained was purified using a QIAGEN mini prep kit. The sequence was confirmed by Sanger sequencing performed by SourceBioscience. The 14-3-3σ C38A protein was expressed and purified as described below.

<u>14-3-3ζ (WT, UniProt: P63104, (pPROEX Htb plasmid)</u>

*MSYYHHHHHHDYDIPTTENLYFQGAMGS*MDKNELVQKAKLAEQAERYDDMAACMKSVTEQGAELSNEERNL
LSVAYKNVVGARRSSWRVVSSIEQKTEGAEKKQQMAREYREKIETELRDICNDVLSLLEKFLIPNASQAESKVFYLK
MKGDYYRYLAEVAAGDDKKGIVDQSQQAYQEAFEISKKEMQPTHPIRLGLALNFSVFYYEILNSPEKACSLAKTA
FDEAIAELDTLSEESYKDSTLIMQLLRDNLTLWTSDTQGDEAEAGEGGEN

<u>Monomeric 14-3-3ζ (^{12}LAE^{14} mutated to ^{12}QQR^{14}) (pET-28a(+) plasmid)</u>

*MGSSHHHHHHDYDIPTTENLYFQGA*MGSMDKNELVQKAK**QQR**QAERYDDMAACMKSVTEQGAELSNEER
NLLSVAYKNVVGARRSSWRVVSSIEQKTEGAEKKQQMAREYREKIETELRDICNDVLSLLEKFLIPNASQAESKVF
YLKMKGDYYRYLAEVAAGDDKKGIVDQSQQAYQEAFEISKKEMQPTHPIRLGLALNFSVFYYEILNSPEKACSLAK
TAFDEAIAELDTLSEESYKDSTLIMQLLRDNLTLWTSDTQGDEAEAGEGGEN

3.2. Protein Expression and Purification

Recombinant His6-tagged 14-3-3 proteins were expressed in BL21 (DE3) competent cells with a pET-28a(+) or pPROEX Htb plasmid. A single transformed colony was used to inoculate 20 mL terrific broth (containing 50 µg/mL kanamycin) which was grown overnight at 37 °C, 180 rpm. The starter culture was used to inoculate 1 L of terrific broth media (containing 50 µg/mL kanamycin) and supplemented with 5 mM $MgCl_2$. The cells were grown at 37 °C, 180 rpm until the OD_{600} reached 0.6 – 0.8. Expression was induced by the addition of 0.4 mM IPTG. Incubation was continued overnight at 25 °C, 180 rpm.

Cells were harvested by centrifugation (5,000 rpm, 4 °C, 20 mins) and resuspended in buffer consisting of 50 mM HEPES pH 8.0, 300 mM NaCl, 12.5 mM Imidazole supplemented with DNAase

and a PierceTM protease inhibitor tablet. The cells were lysed by sonication and the addition of lysozyme. The lysate was cleared by centrifugation (13,000 rpm, 4 °C, 50 mins). The clear lysate was loaded onto a Ni^{2+}-affinity chromatography column equilibrated with 50 mM HEPES pH 8.0, 300 mM NaCl and 12.5 mM imidazole. The Ni^{2+}-affinity chromatography column was washed with 50 mM HEPES pH 8.0, 300 mM NaCl and 25 mM imidazole. The protein was eluted with 50 mM HEPES pH 8.0, 300 mM NaCl and 250 mM imidazole. The protein was dialysed against buffer containing 25 mM HEPES pH 7.5, 100 mM NaCl and 10 mM $MgCl_2$ and concentrated using a 10 kDa cut-off centrifugal filter unit (Merck Millipore).

3.3. Peptide Sequence Information

Peptides were obtained from ChinaPeptides at >95% purity and used without further purification. Peptides were solubilised in water and concentration confirmed by UV/Vis absorptivity using a Thermo Scientific™ NanoDrop™ One Microvolume UV-Vis Spectrophotometer.[2–4]

Sequences:

Peptide	Sequence	ε/$M^{-1}cm^{-1}$ (λ)
ERα-8mer (pT584)	Ac-AEGFPA(pT)V-COOH	28060 (205 nm)
ERα-15mer (pT584)	Ac-KYYITGEAEGFPA(pT)V-COOH	59680 (205 nm)
TAMRA-ERα (pT584)	TAMRA-βA-AEGFPA(pT)V-COOH	90000 (552 nm)
TAMRA-p53 (pT387)	TAMRA-Acp-SRAHSSHLKSKKGQSTSRHKKLMFK(pT)EGPDSD-COOH	90000 (552 nm)
Cy5-LRRK2 (pS395)	Cy5-NLQRHSNpSLGPIFDH-COOH	250000 (650 nm)
Cy5-AHA2 (T947)	Cy5-VKLKGLDIETSHYpTV-COOH	250000 (650 nm)

4. Biophysical Assays

4.1. Mass Spectrometry Experiments

The 14-3-3 proteins and the ligands (**1** – **8**) were diluted into buffer (25 mM HEPES pH 7.5, 100 mM NaCl, 10 mM $MgCl_2$), to give a final protein concentration of 100 µM and a final ligand concentration of 100 µM (1% v/v DMSO). Samples were incubated at room temperature (r.t.) for 24 h. 8.0 µL of each sample was diluted with 192 µL H_2O containing 0.1% v/v formic acid to give a final protein concentration of 0.1 mg/mL. Further validation experiments were conducted in the presence of 100 µM 14-3-3σ/ERα-8mer complex (1:1) and 100 µM or 1000 µM of **7**. Mass spectra were recorded on a Waters Acquity XEVO Q ToF instrument, processed using MassLynx and deconvoluted using the MaxEnt function.

4.2. Fluorescence Polarisation Assay

Fluorescence Polarisation (FP) measurements were performed using Corning black, round- bottom, low-binding 384-well plates in FP buffer (25 mM HEPES, 100 mM NaCl, 10 mM $MgCl_2$, 0.1% (v/v) Tween20, 0.1% (m/v) BSA, pH 7.4). The concentration of fluorescently labelled phosphopeptides was kept constant at 10 nM for all experiments. Protein titration experiments were performed using a fixed concentrations of molecular glue as indicated. Molecular glue titration experiments were performed at a fixed of 14-3-3σ (50 nM). Experiments were performed in triplicate using 1% DMSO (v/v). Plates were incubated at room temperature for 30 min and shaken for 10 seconds before

measurement using a Clariostar Plus Microplate Reader. Optical parameters were set accordingly to each fluorescent tracer. TAMRA: λ_{Ex} = 540 /20 nm; λ_{Em} = 590 /20 nm; dichroic mirror 566. FITC: λ_{Ex} = 482/16 nm; λ_{Em} = 530/40 nm; dichroic mirror 504. Cy5: λ_{Ex} = 635/20 nm; λ_{Em} = 680/20 nm; dichroic mirror 659. For all experiments, Nº flashes = 30; PMT voltage = 750; Z-position = calculated from well. Data were corrected from FP buffer and analysed in GraphPad Prism 10 and sigmoidal curves were fitted using the Levenburg-Marquardt iteration algorithm.

4.3. Fluorescent Thermal Stability Assay

Thermal stability assay (TSA) was performed using 0.1mL transparent 96-well PCR plates (ThermoFisher). Each sample (25 µL) contained 14-3-3σ (25 µM), ERα 15mer phosphopeptide (0, 50, or 125 µM), varied concentrations of molecular glues, and SYPRO Orange (Invitrogen) to a final dilution of 20x in buffer (25 mM HEPES pH 7.5, 100 mM NaCl, 10 mM $MgCl_2$) with 1% DMSO (v/v).The experiments were set in at least four replicates and the plate was incubated for 30 minutes before data collection in a qPCR QuantStudioTM 3 system (λex = 472 nm; λex = 570 nm). Samples were heated from 20.0°C to 95 °C at 0.05°C/s heating increments for 26 minutes. The data was plotted using GraphPad Prism 10. Melting curves were plotted as arbitrary fluorescence intensity (Fl) vs temperature (T, [°C]), and the melting temperatures (Tm) were extracted as the minimum values observed for the negative derivative of fluorescence intensity over temperature (-d[Fl]/d[T]). The mean Tm values were calculated as the arithmetic average of at least 4 replicates and the statistic analysis was done using Graphpad Prism 10 built-in One-way ANOVA with the Dunett's test for the calculation of the P values (ns = P>0.1; * = P<0.1, ** = P<0.01, *** = P<0.001, **** = P<0.0001).

5. ^{1}H, ^{13}C, and HRMS spectra

5.1. Compound 1: *N*-(3-aminopropyl)-2-chloro-5-nitrobenzamide

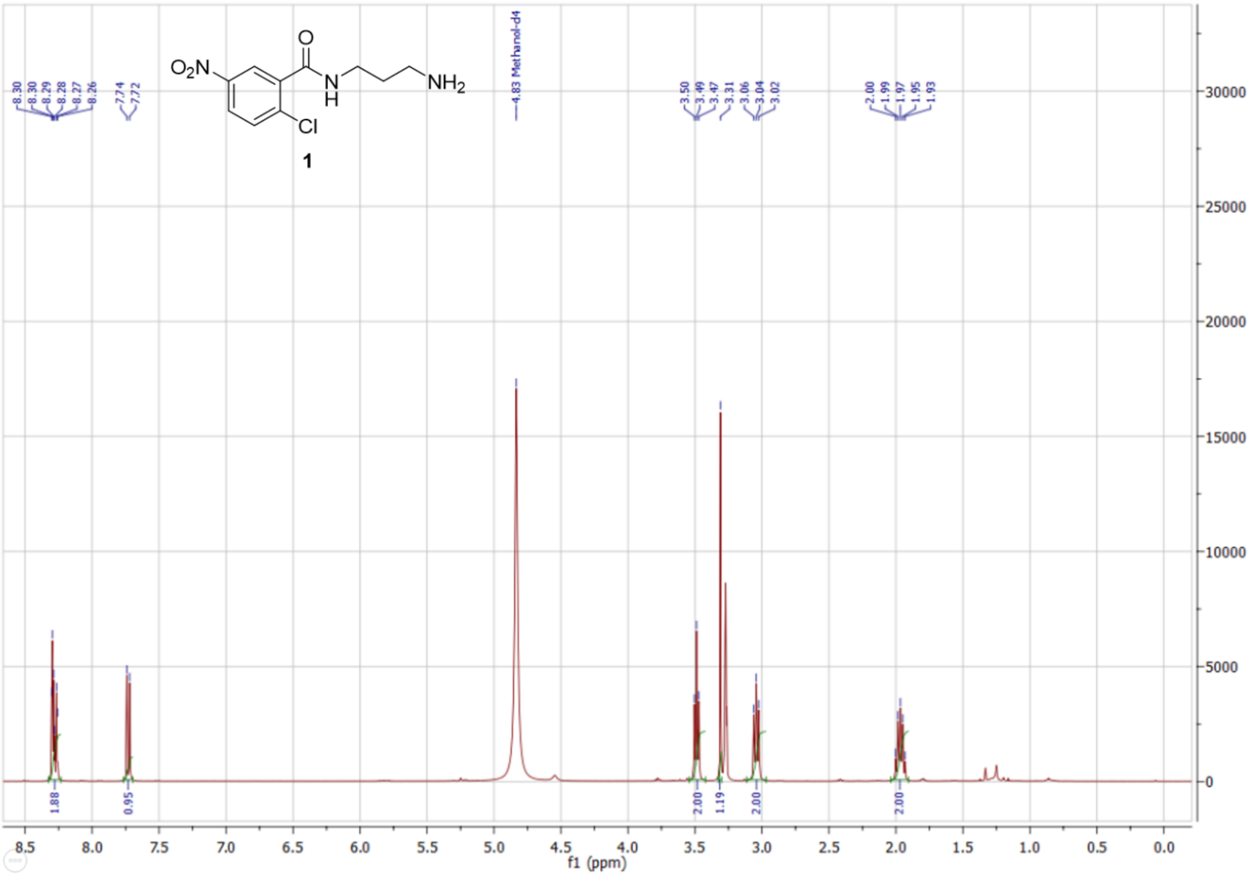

Figure S24. ^{1}H NMR (400 MHz, CD_3OD) of **1**.

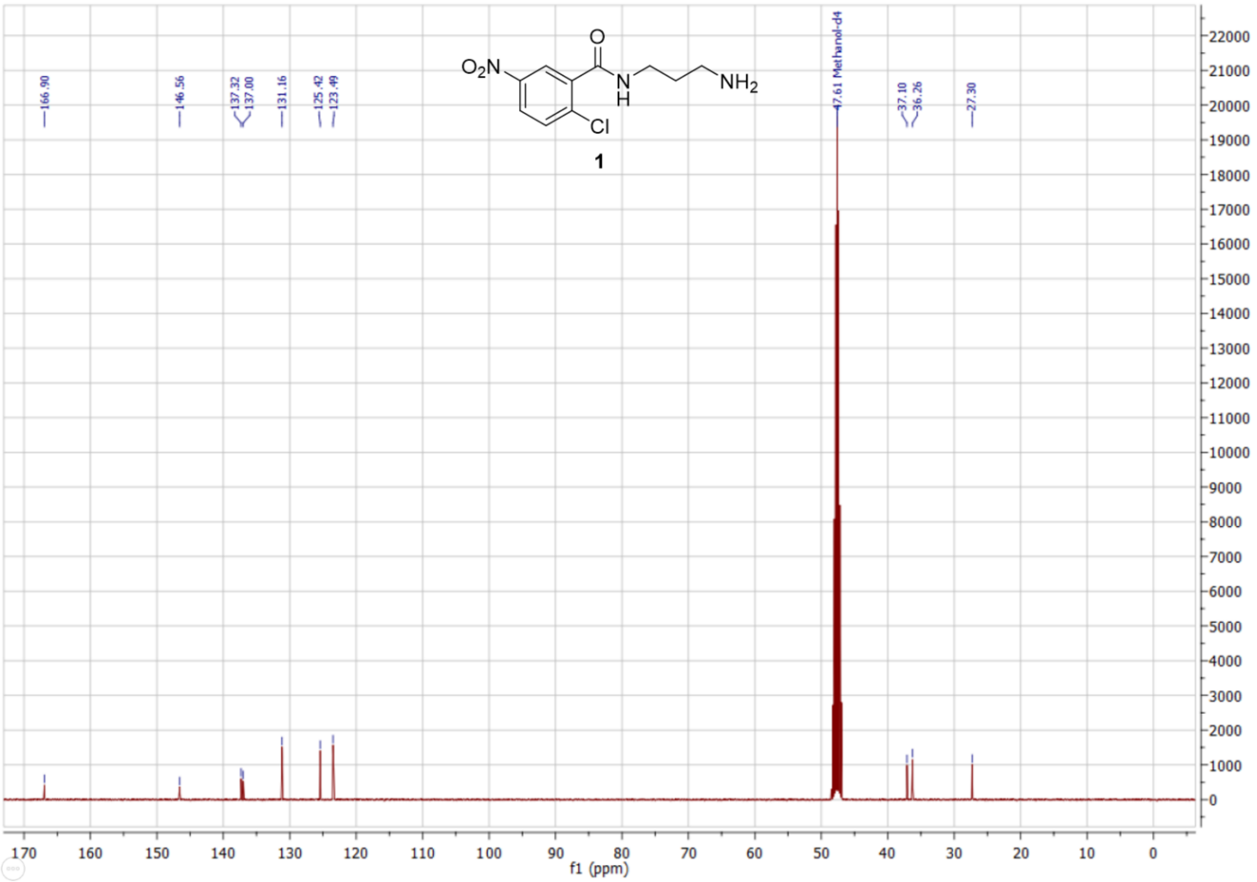

Figure S25. ^{13}C NMR (100 MHz, CD_3OD) of **1**.

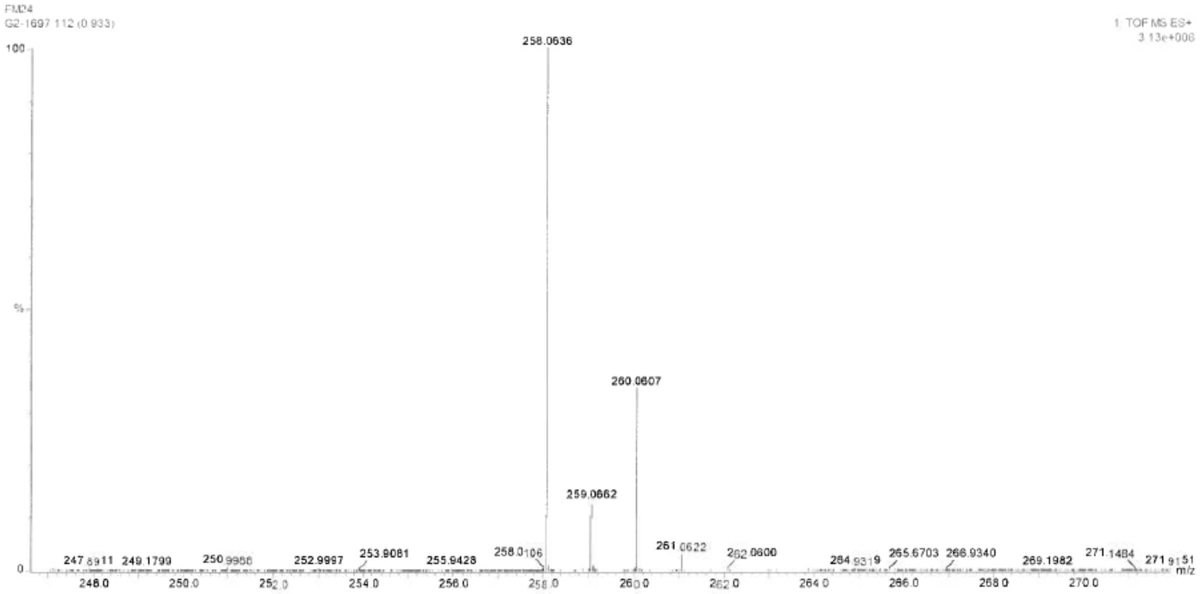

Figure S26. HMRS of **1**.

5.2. Compound 2: *N*-(3-aminopropyl)-2-iodoacetamide

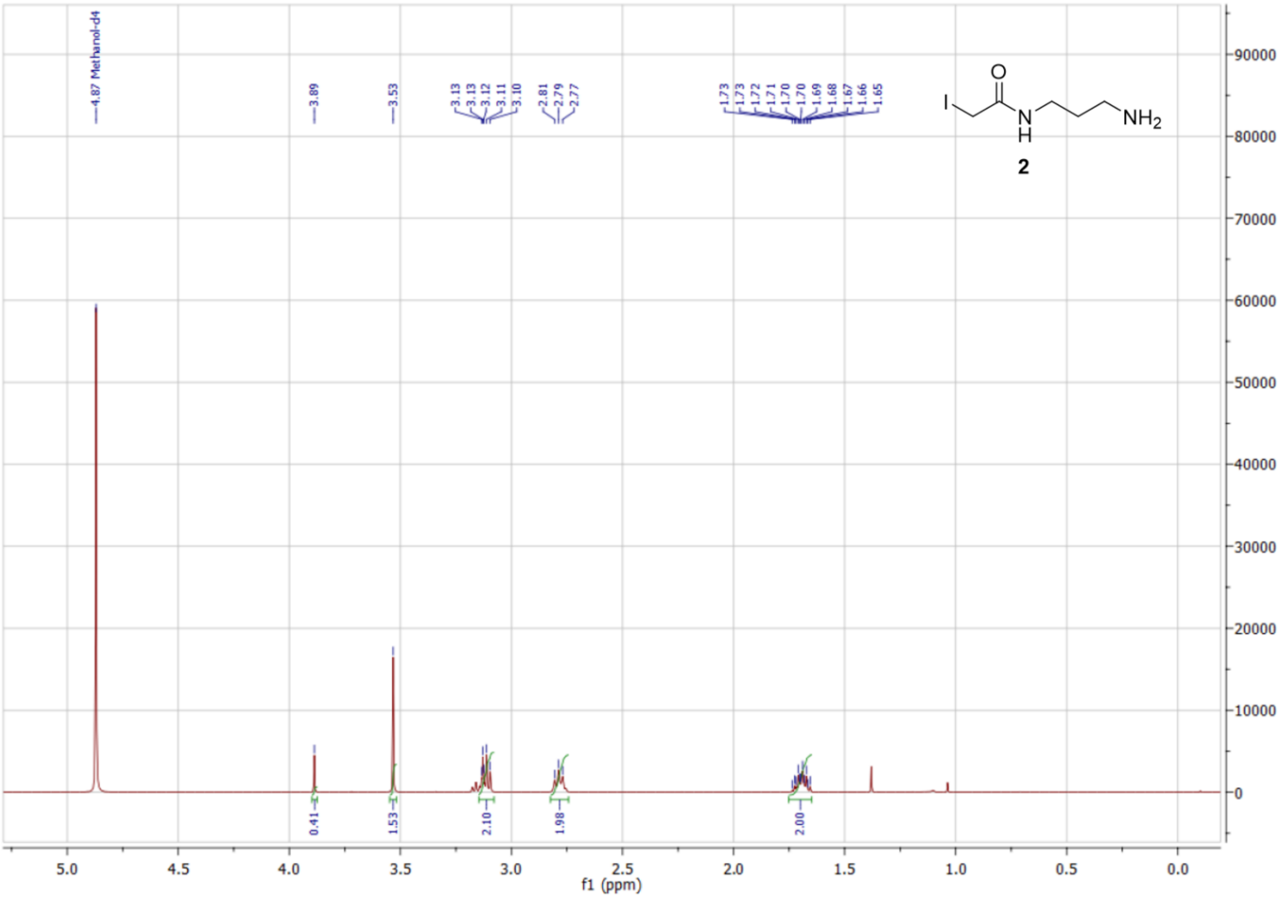

Figure S27. ^{1}H NMR (400 MHz, CD_3OD) of **2**.

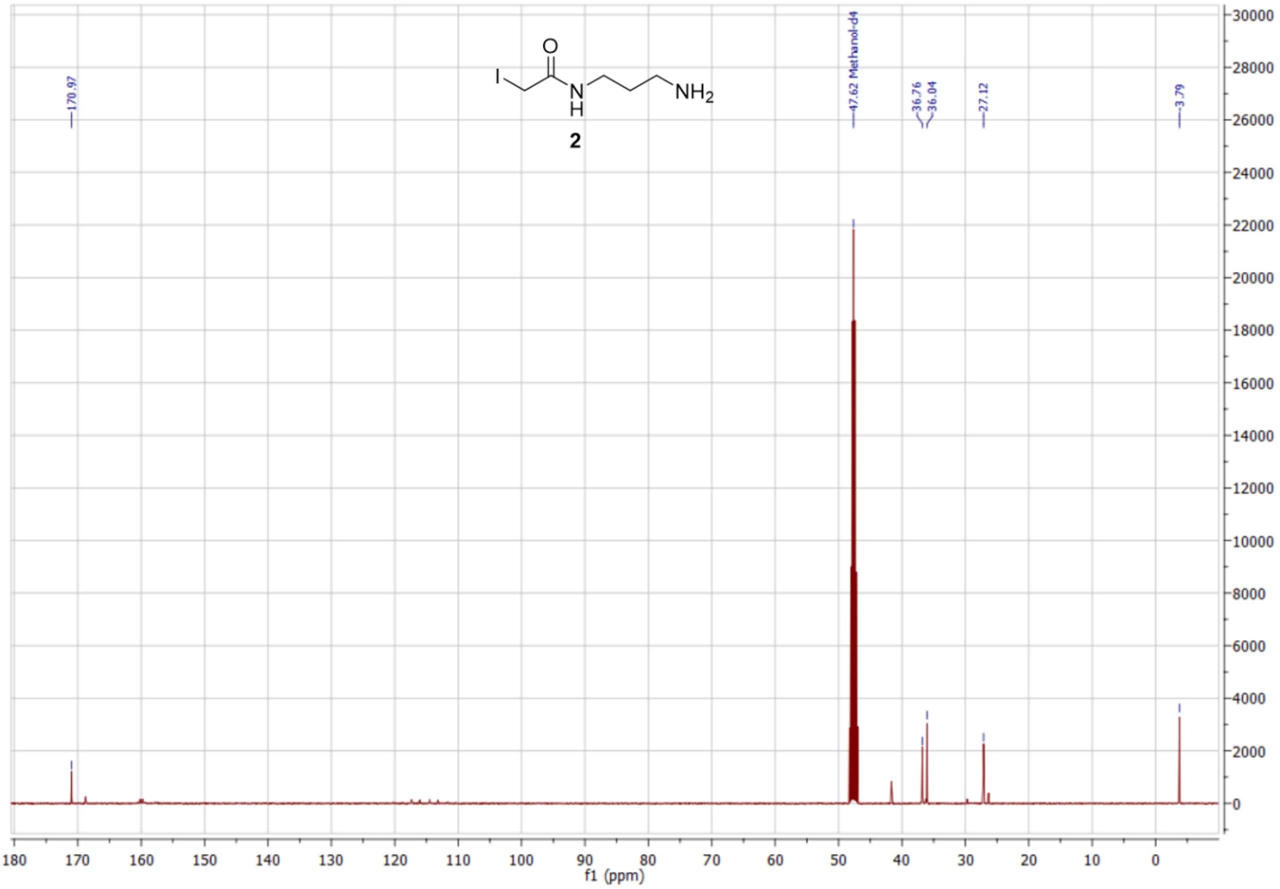

Figure S28. ^{13}C NMR (100 MHz, CD_3OD) of **2**.

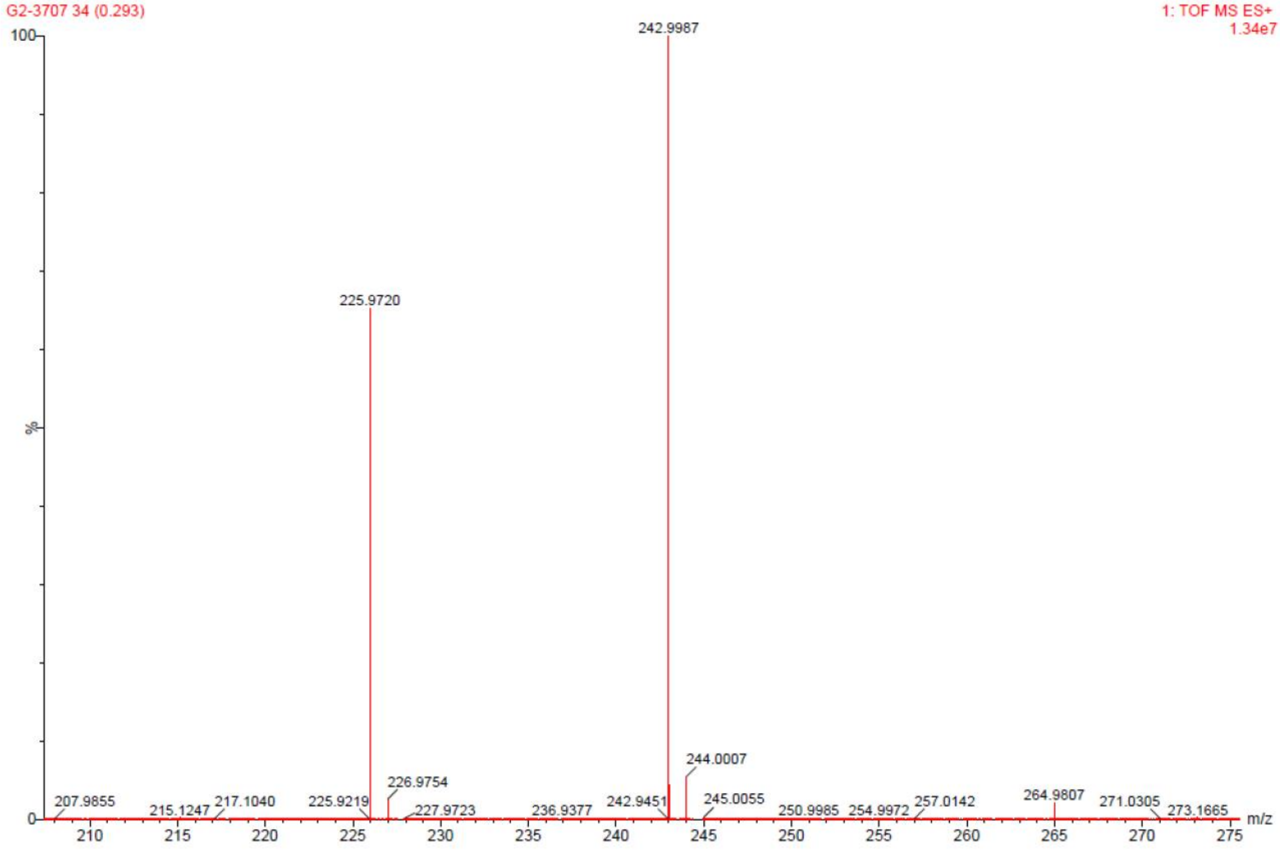

Figure S29. HMRS of **2**.

5.3. Compound 3: 1-(2-aminoethyl)-1H-pyrrole-2,5-dione

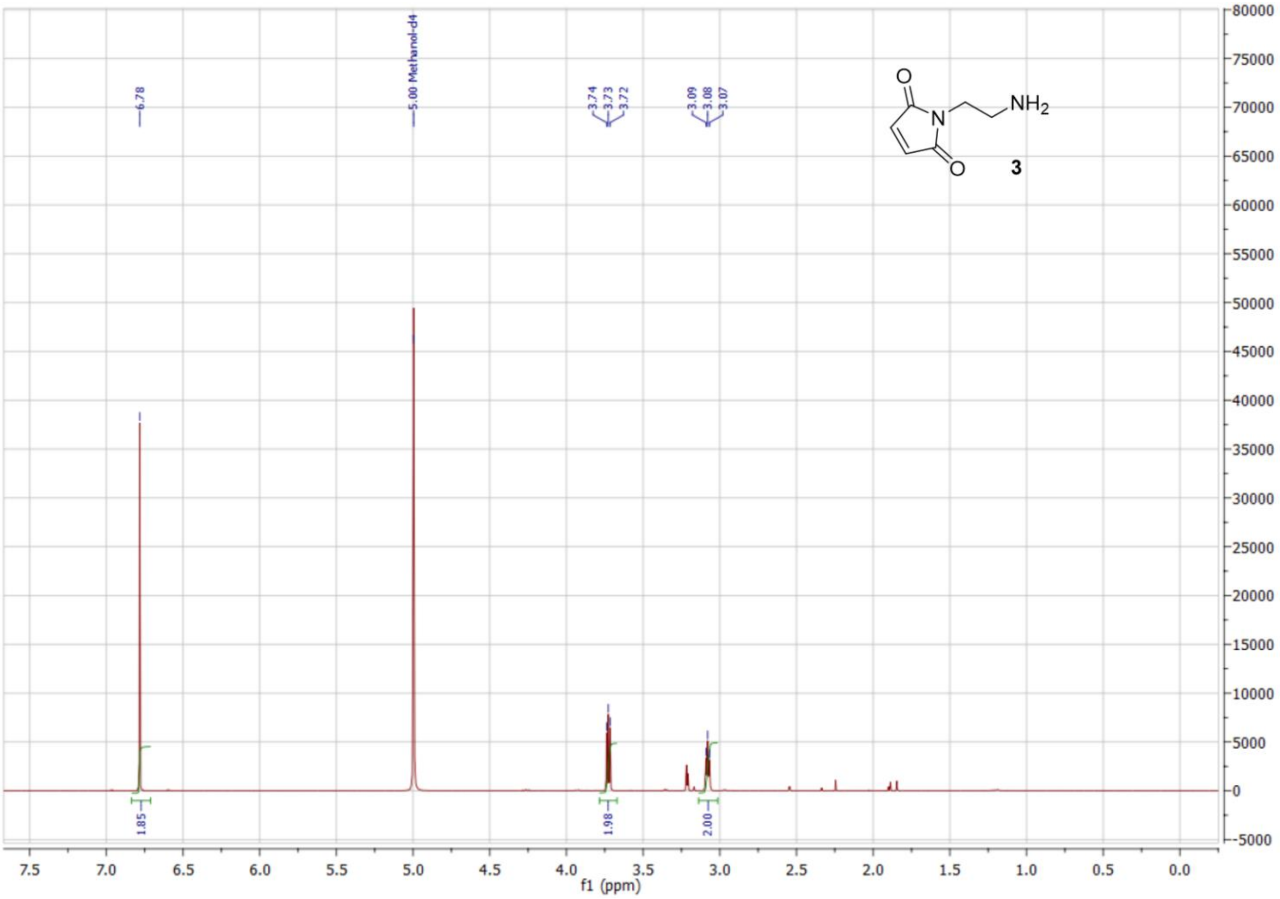

Figure S30. ^{1}H NMR (500 MHz, CD_3OD) of **3**.

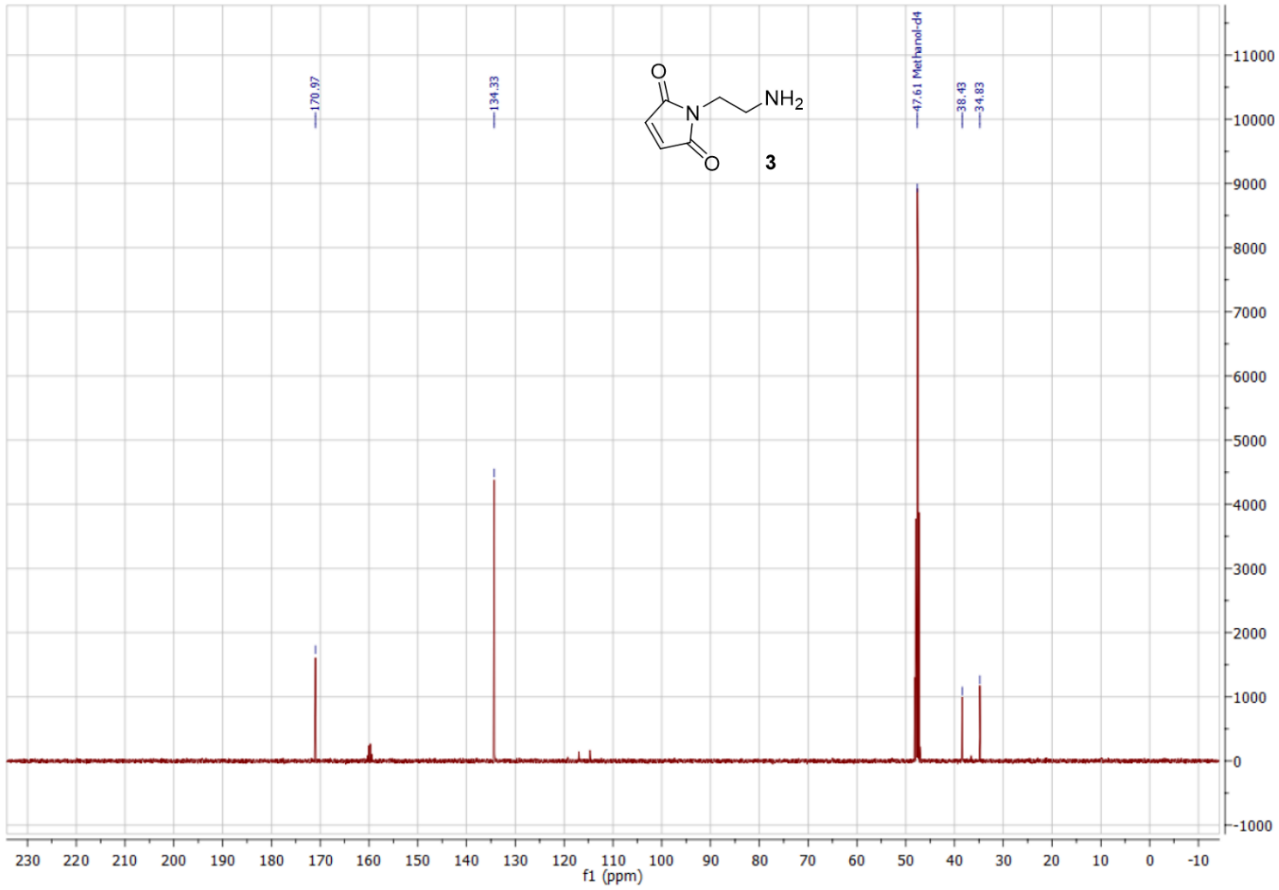

Figure S31. ^{13}C NMR (125 MHz, CD_3OD) of **3**.

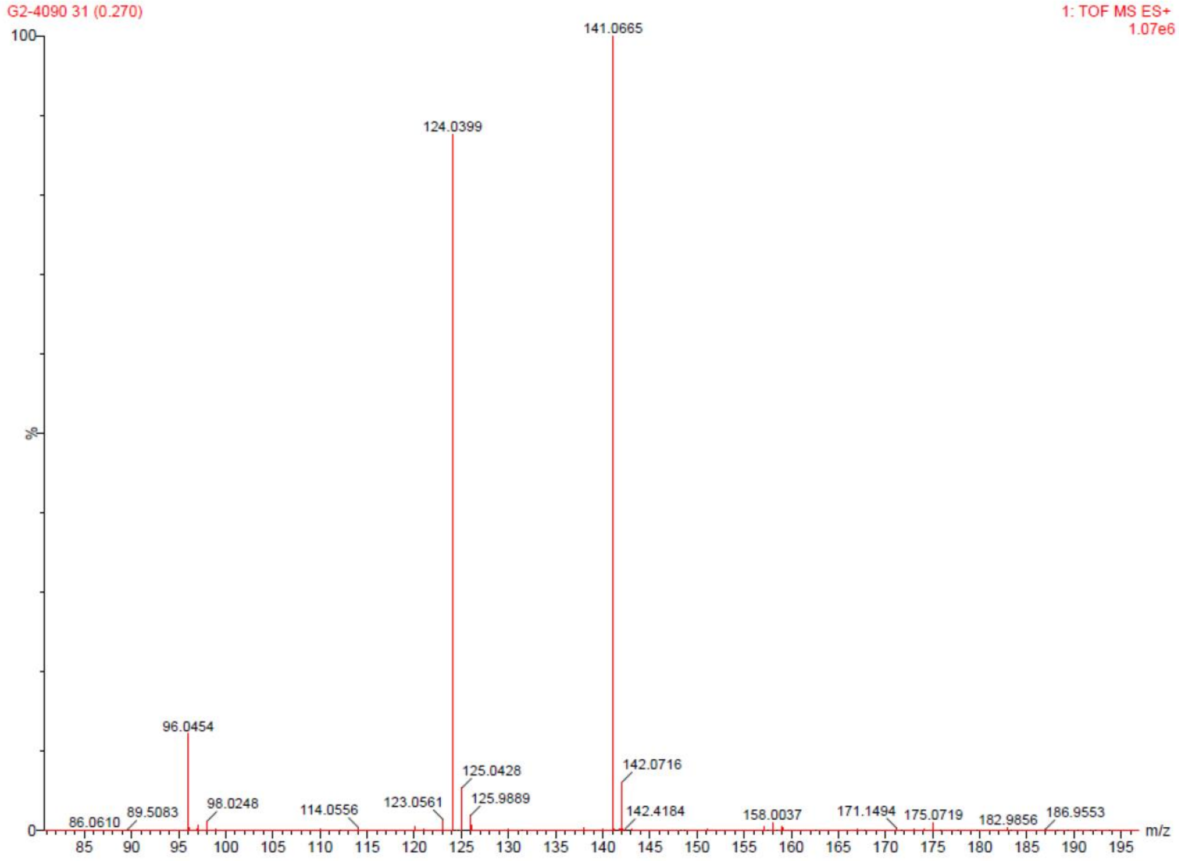

Figure S32. HMRS of **3**.

5.4. Compound 4: 1-(3-aminopropyl)-1H-pyrrole-2,5-dione

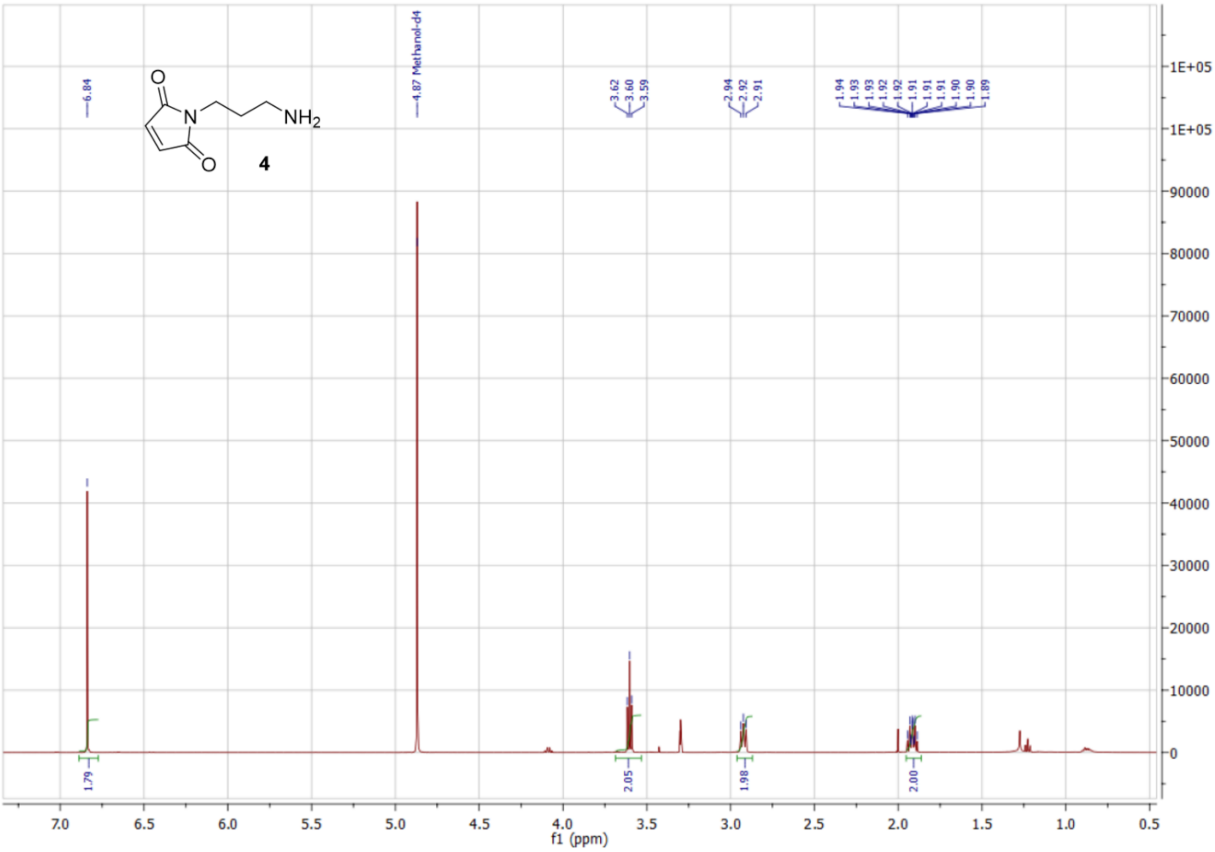

Figure S33. ^{1}H NMR (500 MHz, CD_3OD) of **4**.

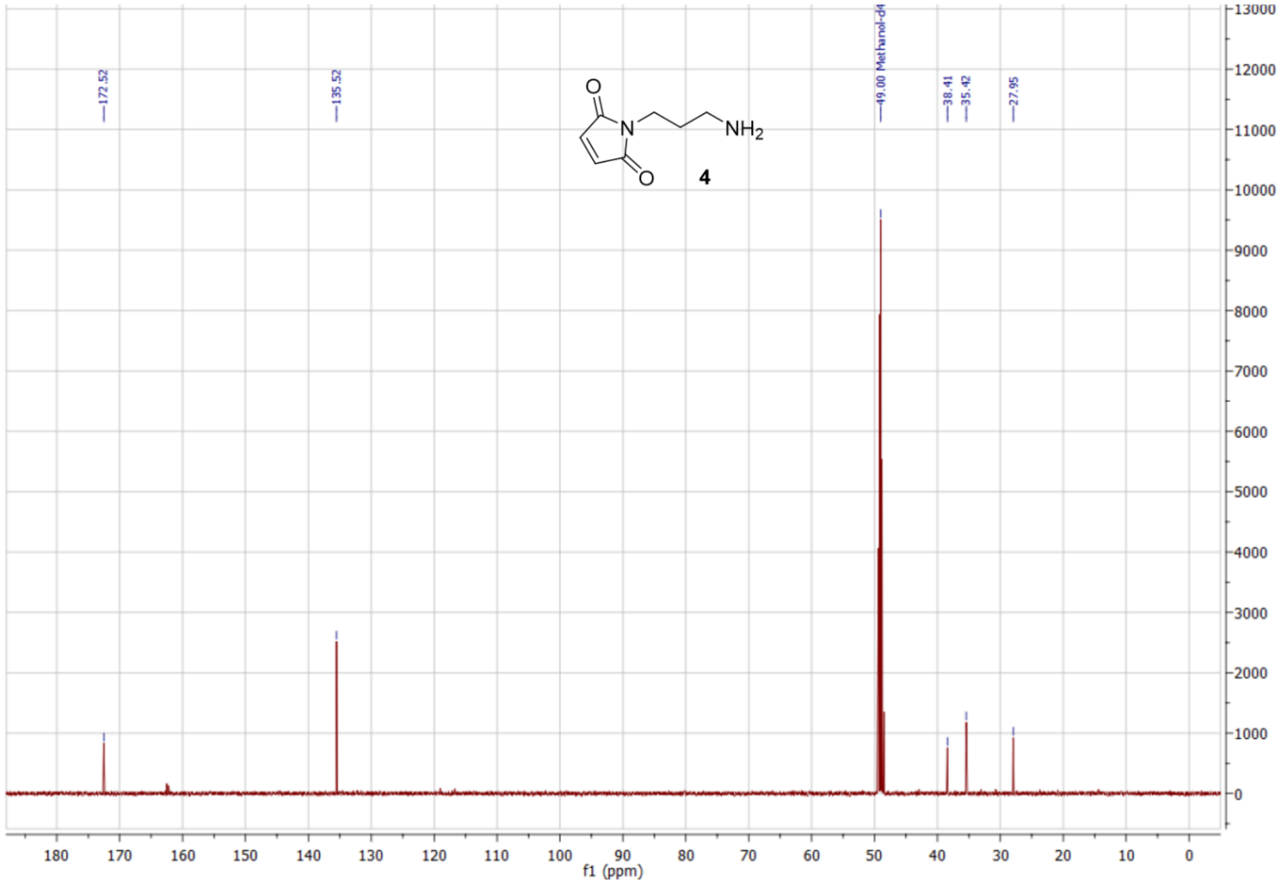

Figure S34. ^{13}C NMR (125 MHz, CD_3OD) of **4**.

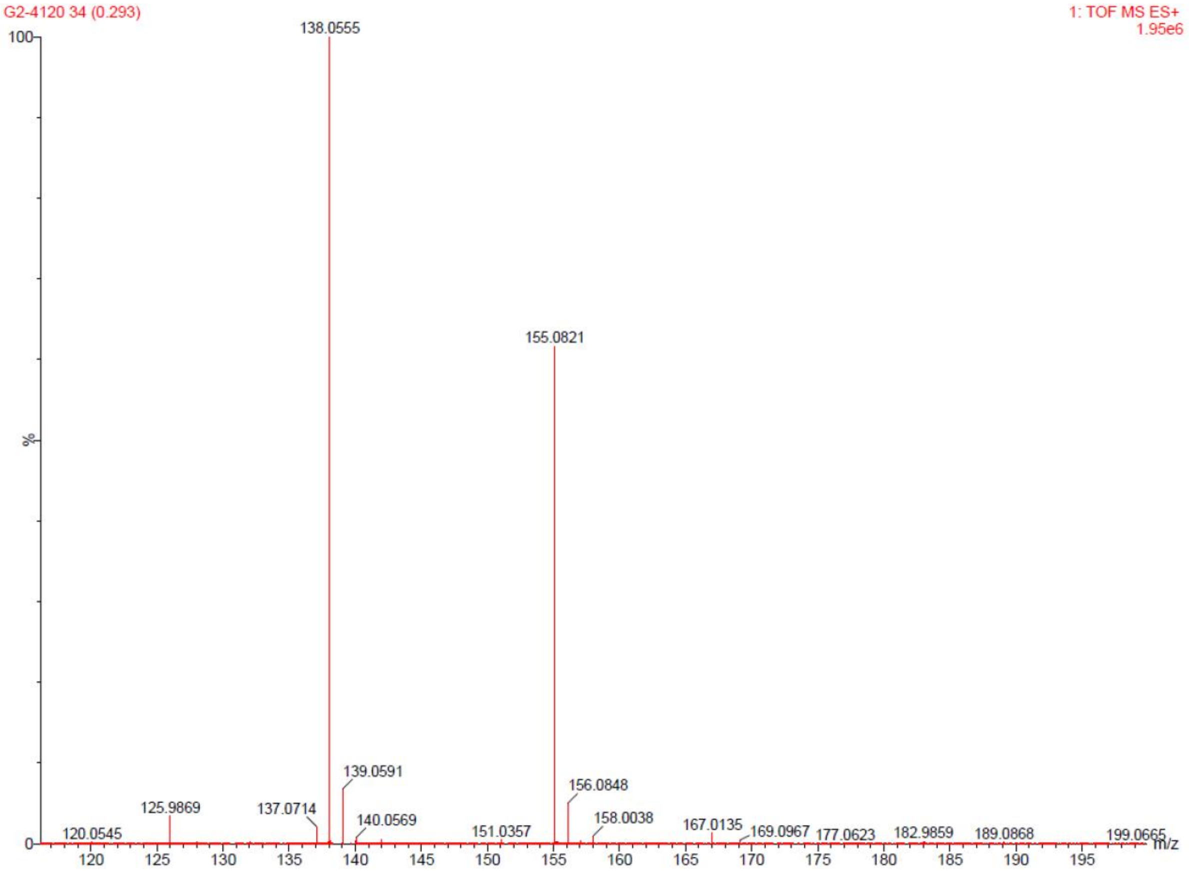

Figure S35. HMRS of **4**.

5.5. Compound 5: *N*-(2-aminoethyl)ethenesulfonamide

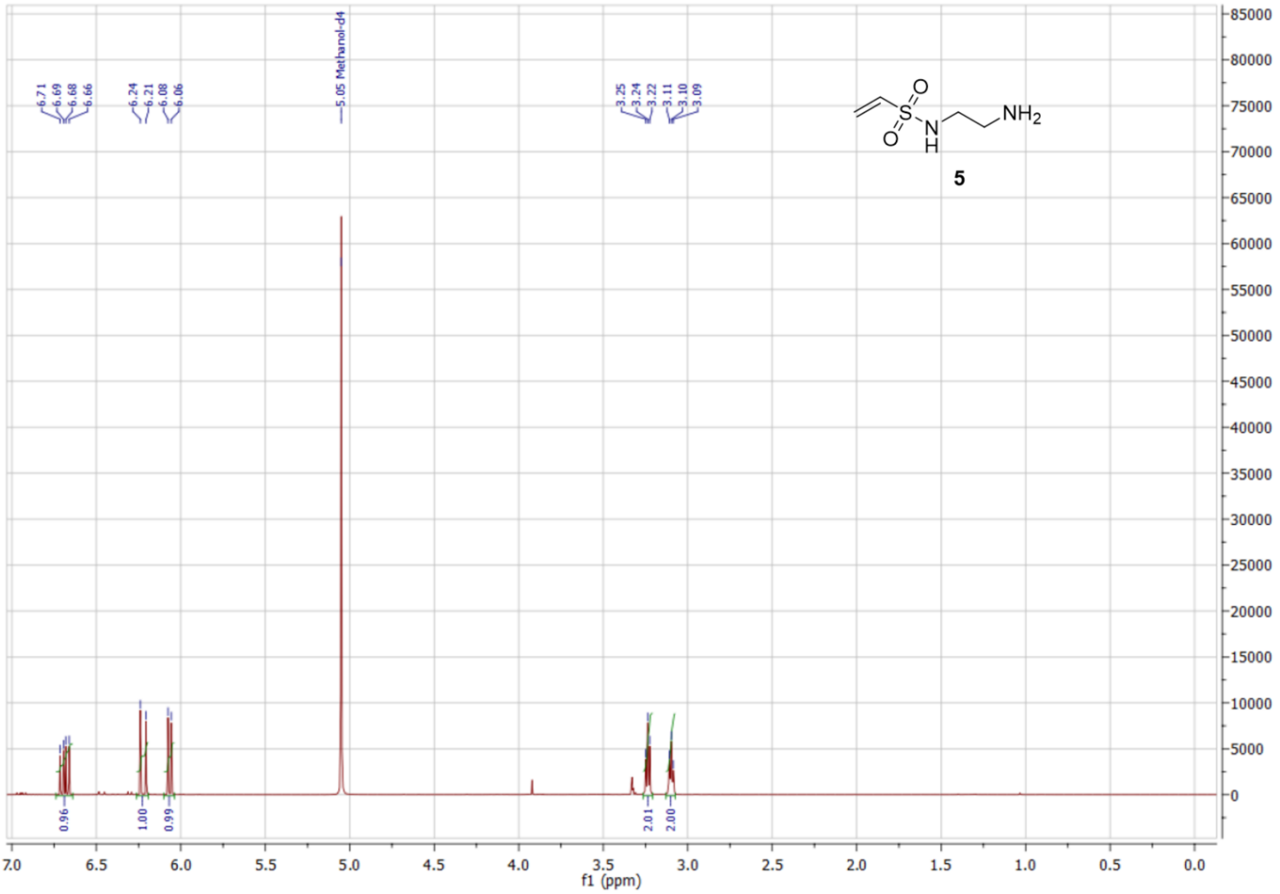

Figure S36. ^{1}H NMR (500 MHz, CD_3OD) of **5**.

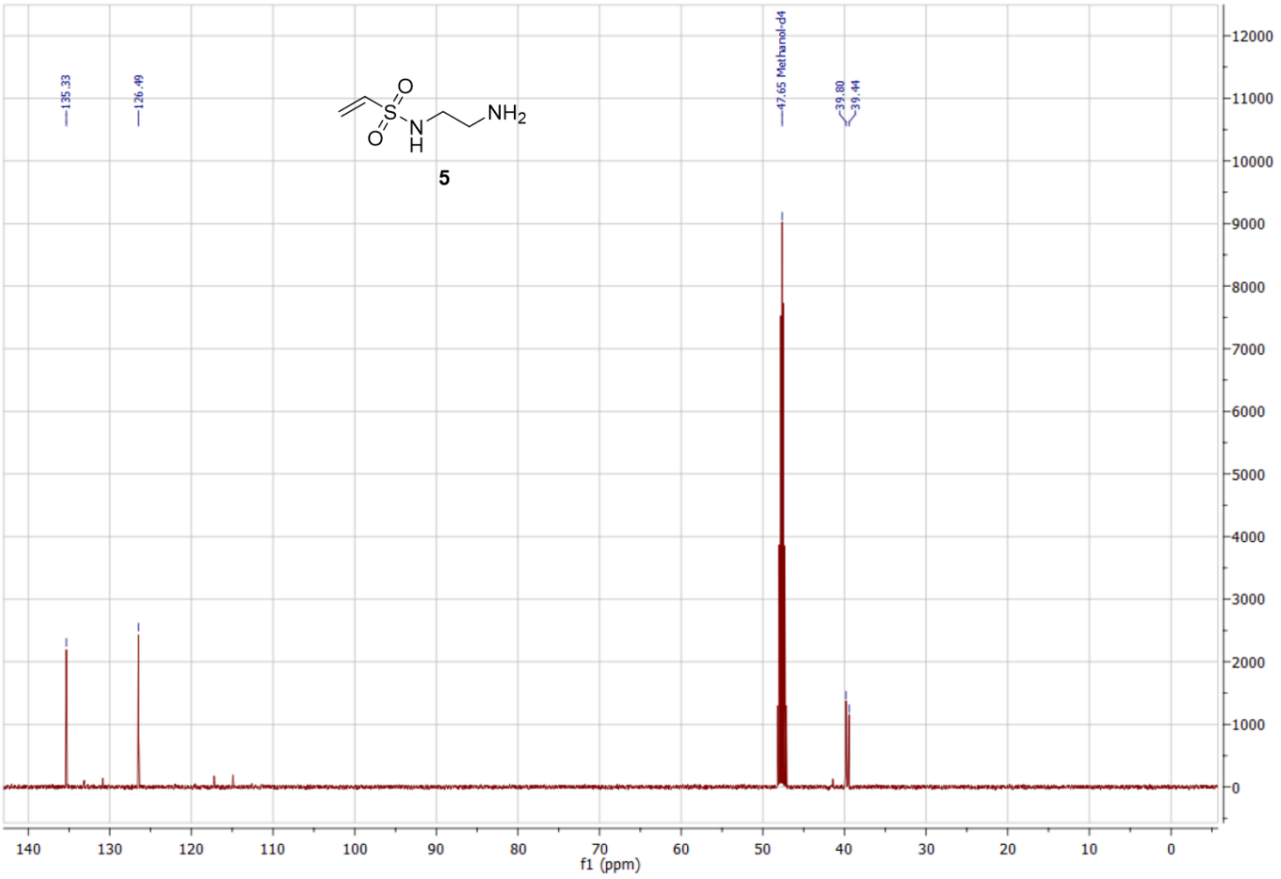

Figure S37. ^{13}C NMR (125 MHz, CD_3OD) of **5**.

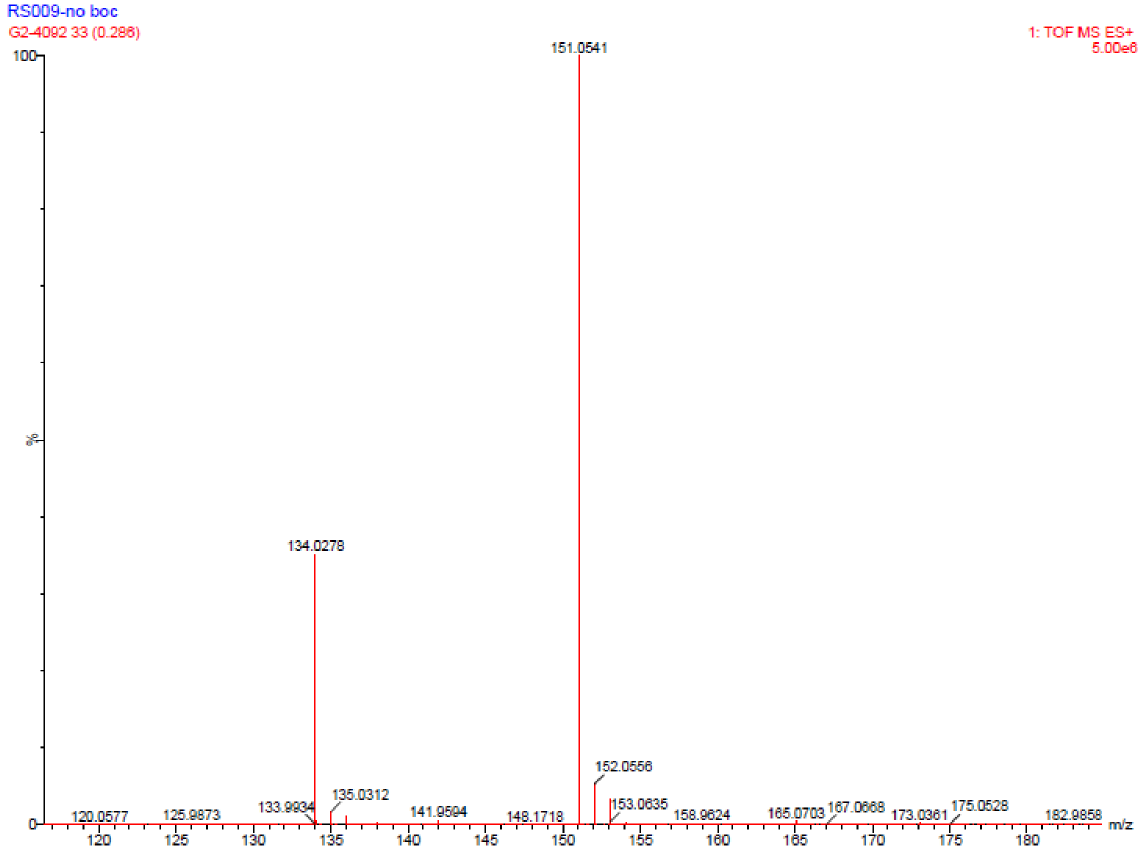

Figure S38. HRMS of **5**.

5.6. Compound 6: *N*-(3-aminopropyl)ethenesulfonamide

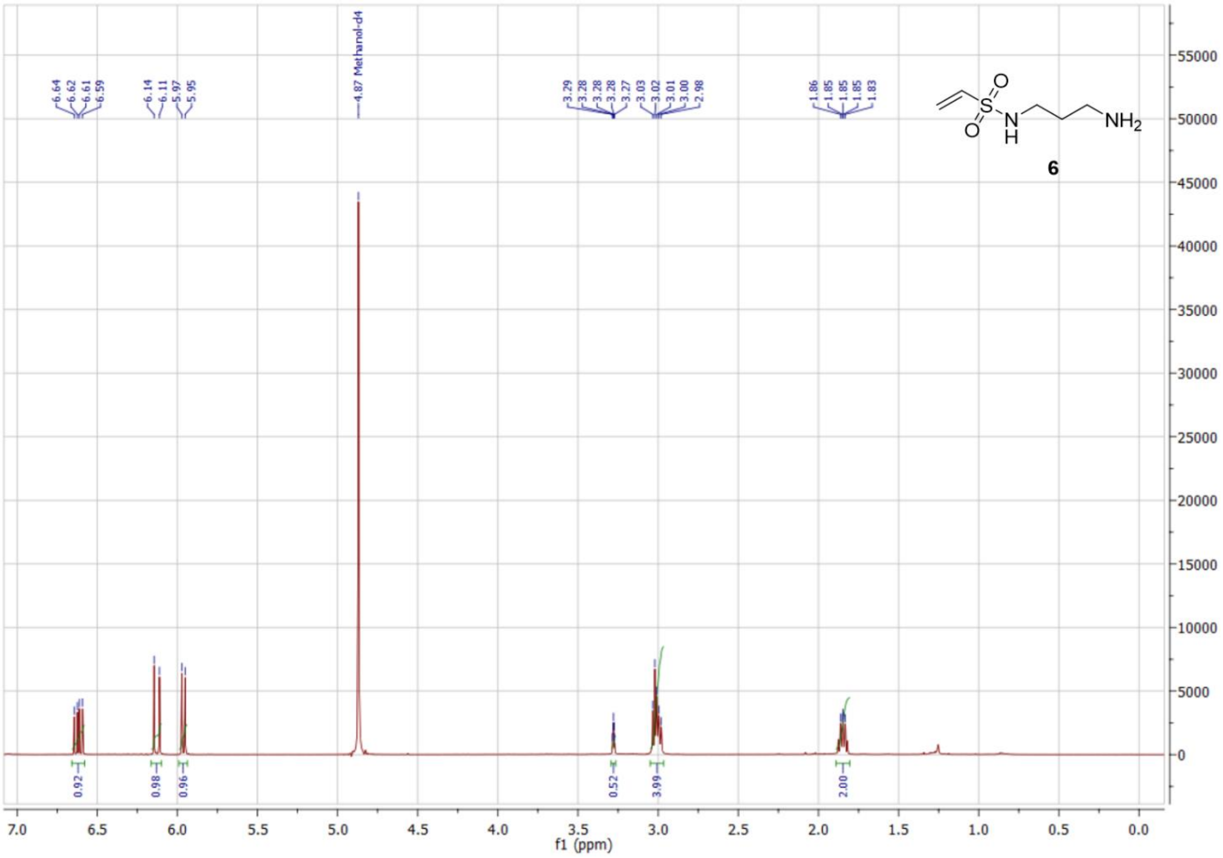

Figure S39. ^{1}H NMR (500 MHz, CD_3OD) of **6**.

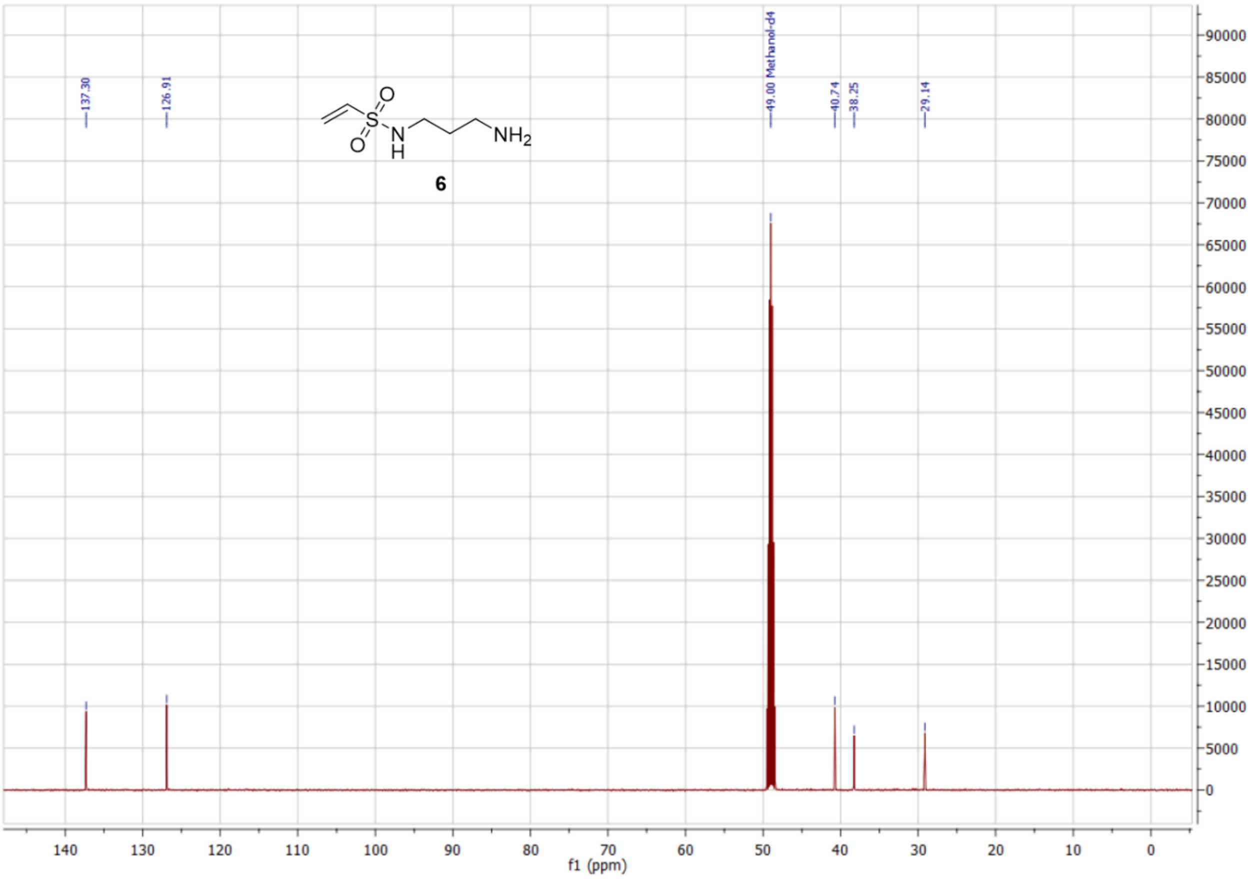

Figure S40. ^{13}C NMR (125 MHz, CD_3OD) of **6**.

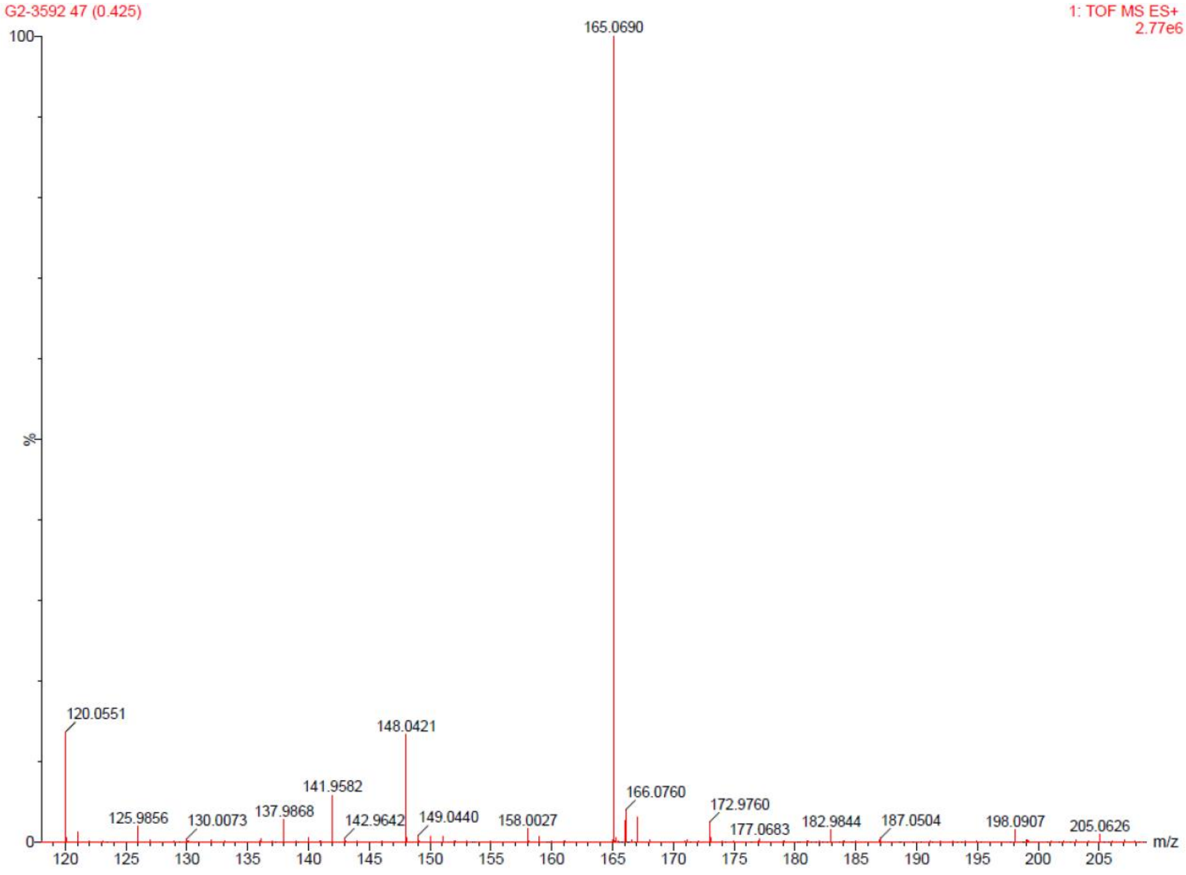

Figure S41. HMRS of 6.

5.7. Compound 7: *N*-(2-aminoethyl)acrylamide

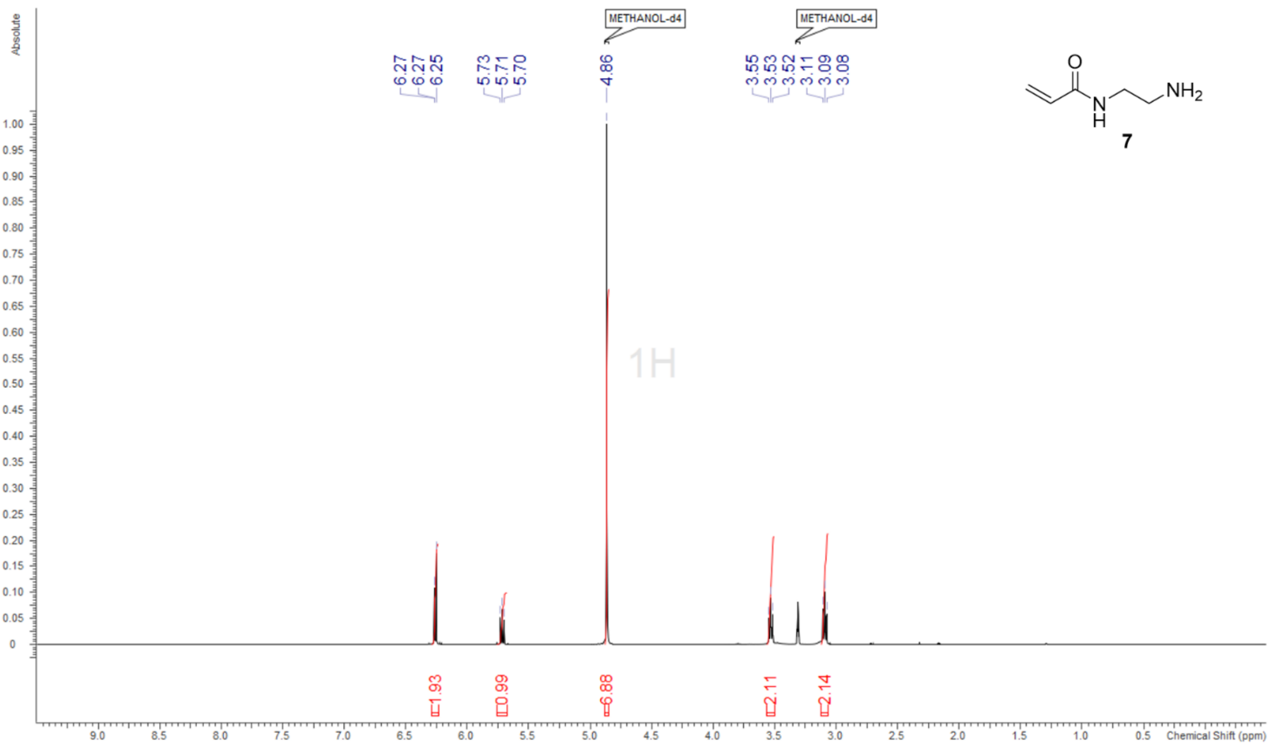

Figure S42. ^{1}H NMR (400 MHz, CD_3OD) of **7**.

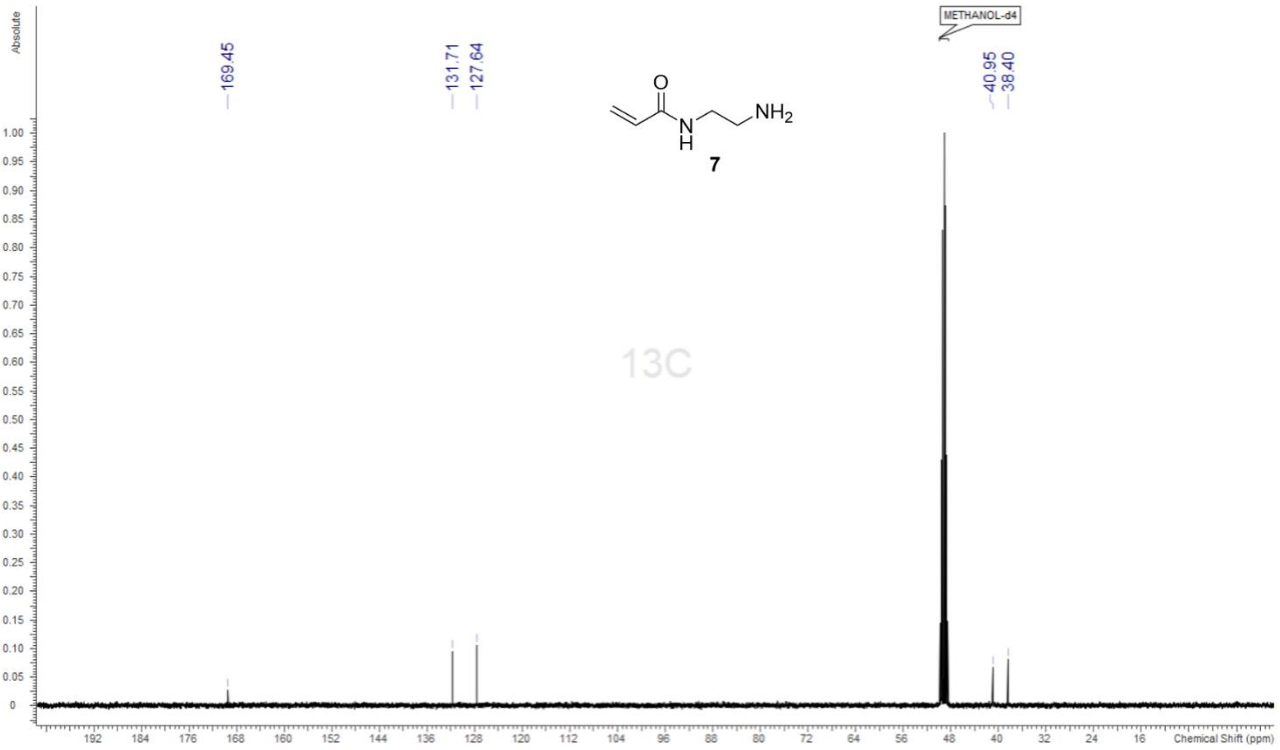

Figure S43. ^{13}C NMR (100 MHz, CD_3OD) of **7**.

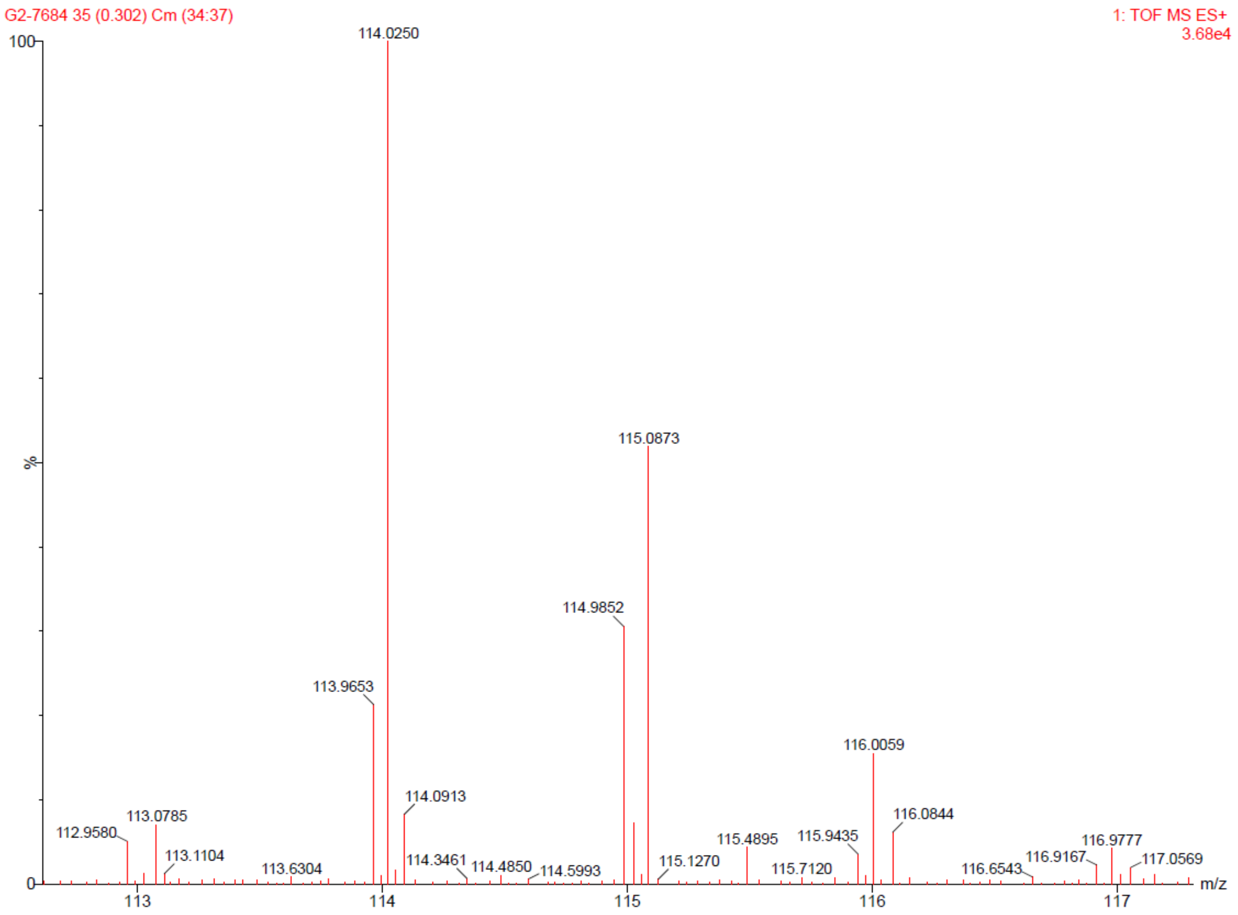

Figure S44. HMRS of **7**.

5.8. Compound 8: *N*-(2-aminopropyl)acrylamide

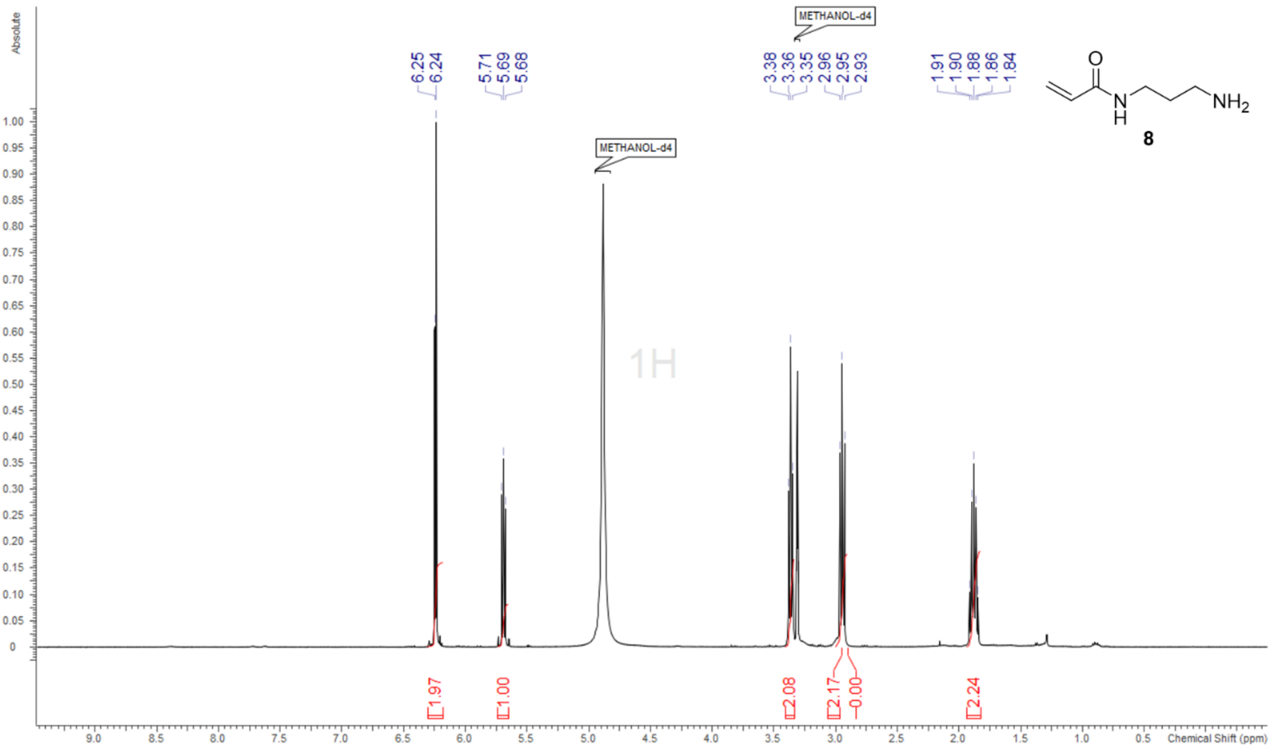

Figure S45. ^{1}H NMR (400 MHz, CD_3OD) of **8**.

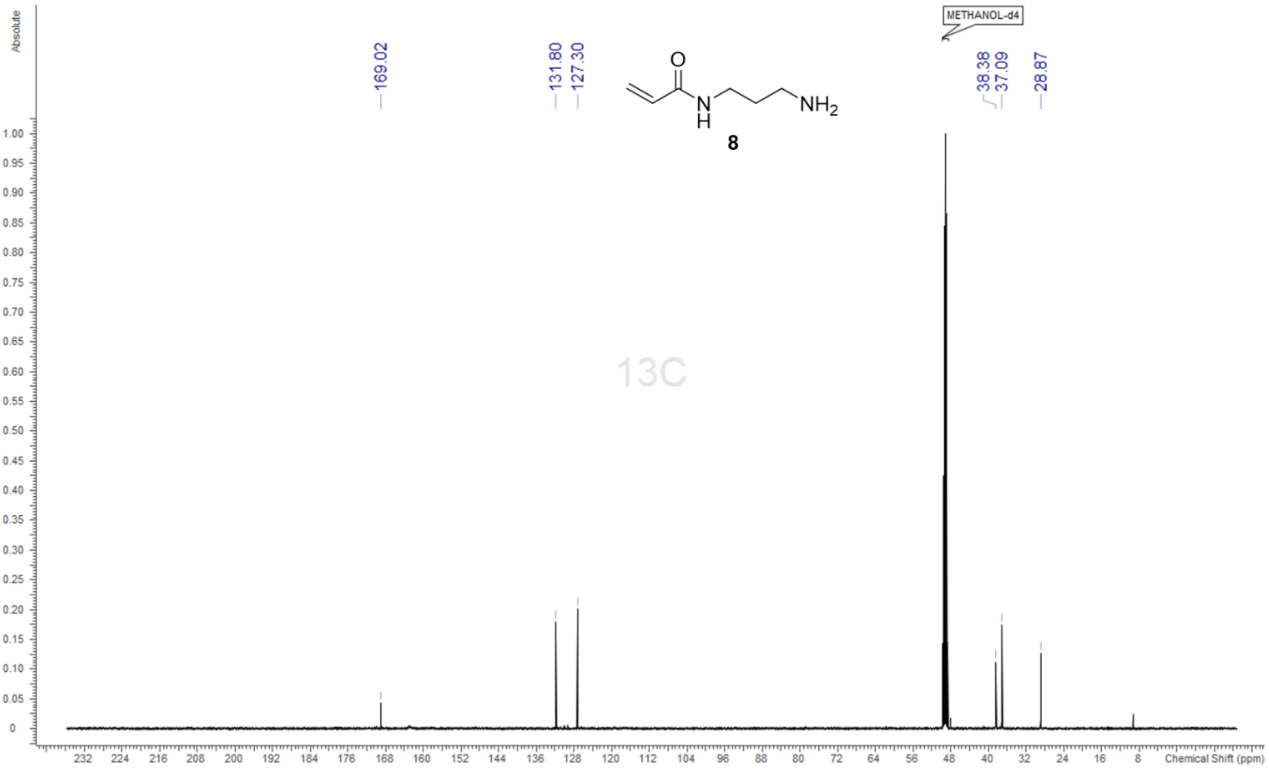

Figure S46 ^{13}C NMR (100 MHz, CD_3OD) of **8**.

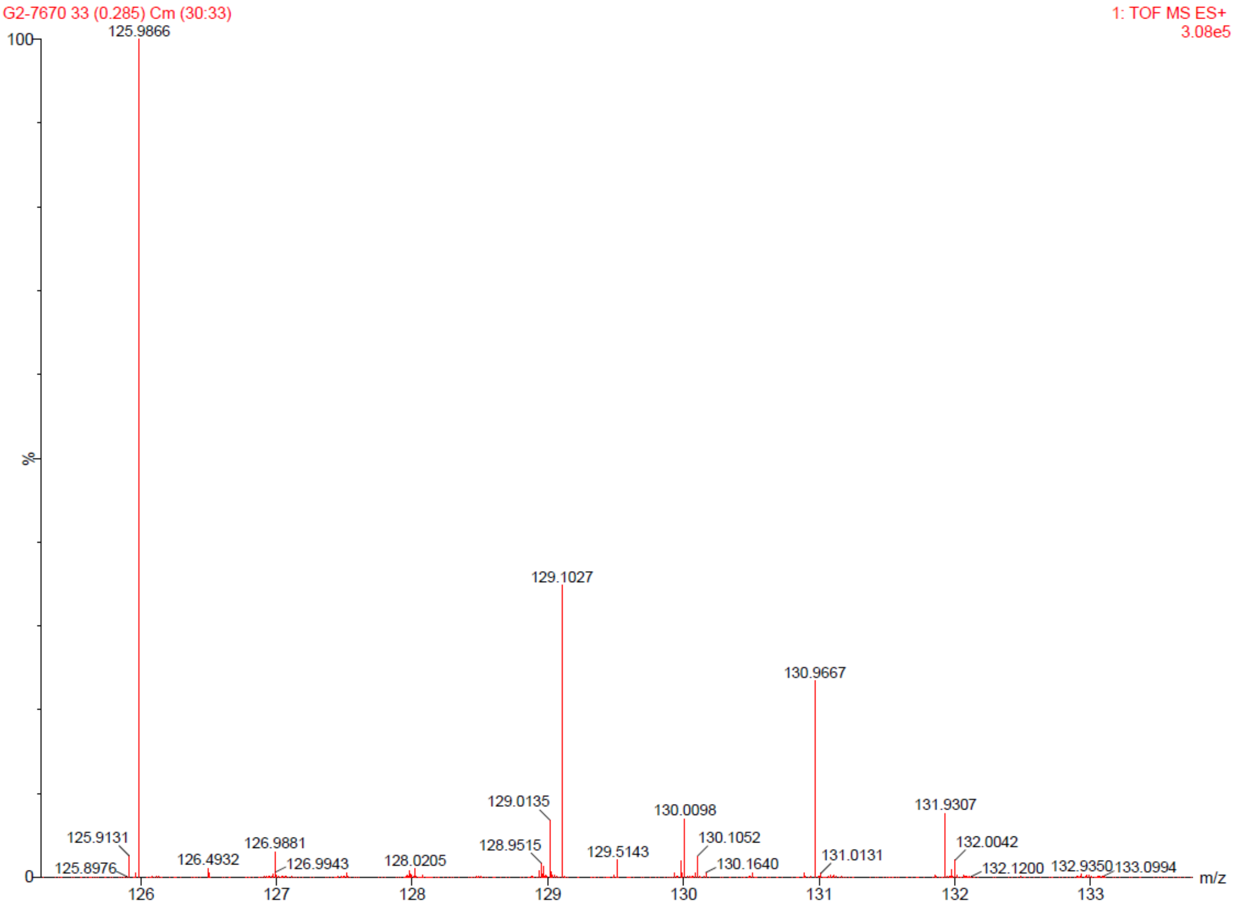

Figure S47. HMRS of **8**.

5.9. Compound 9: *N*-(2-aminobutyl)acrylamide

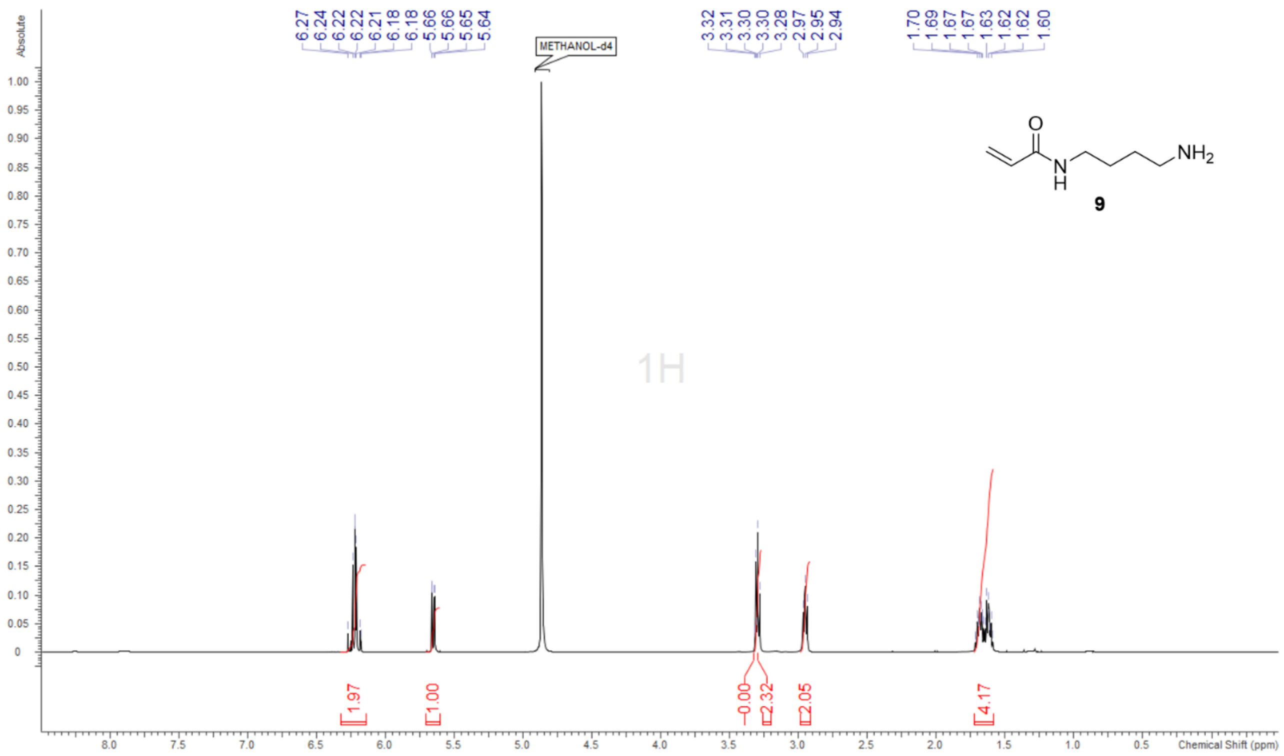

Figure S48. ^{1}H NMR (400 MHz, CD_3OD) of **9**.

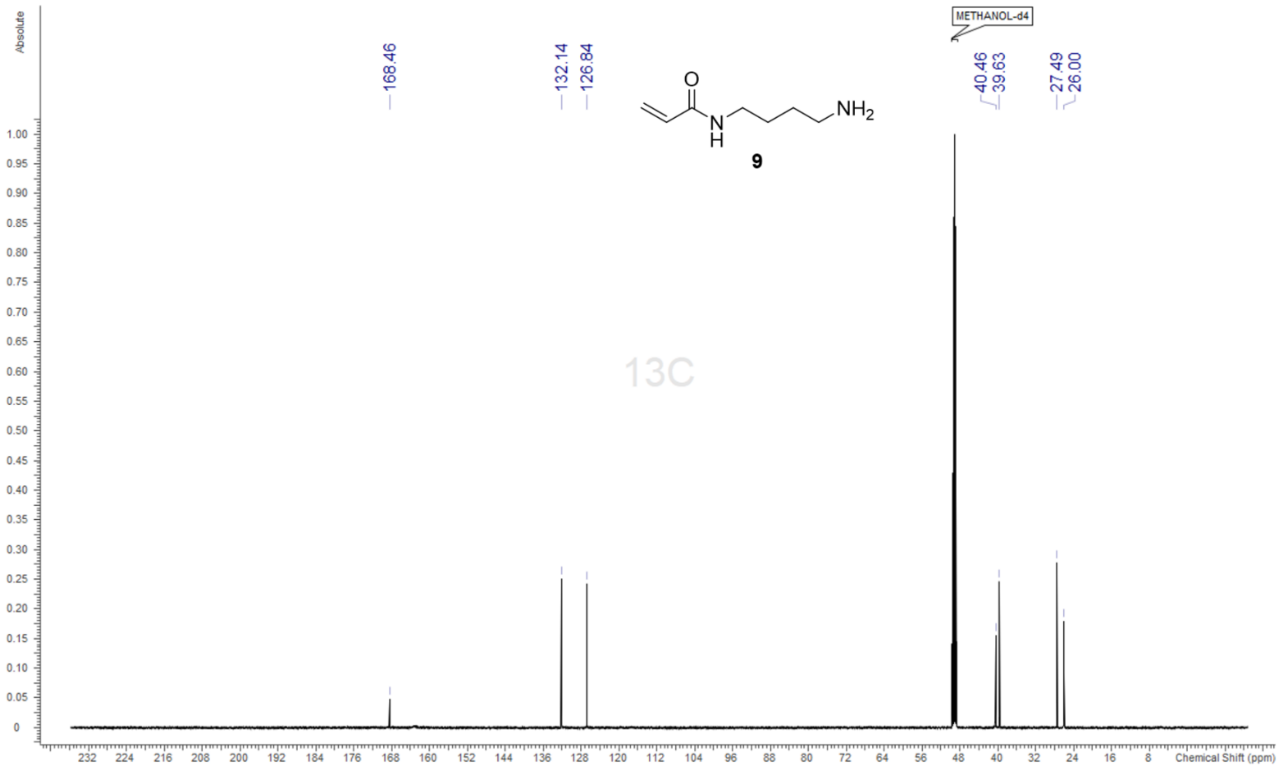

Figure S49 ^{13}C NMR (100 MHz, CD_3OD) of **9**.

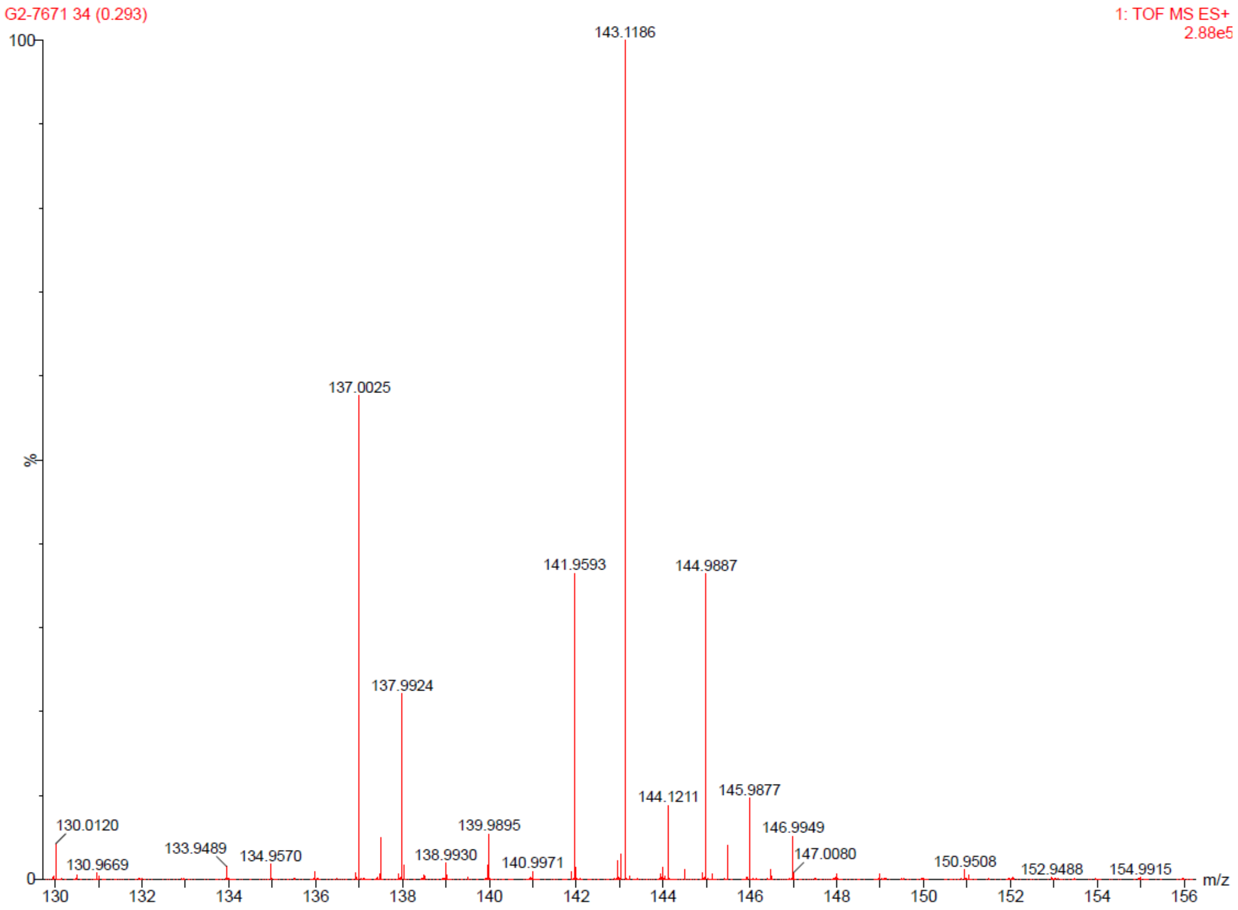

Figure S50. HMRS of **9**.

5.10. Compound 10: 1-(piperazin-1-yl)prop-2-en-1-one

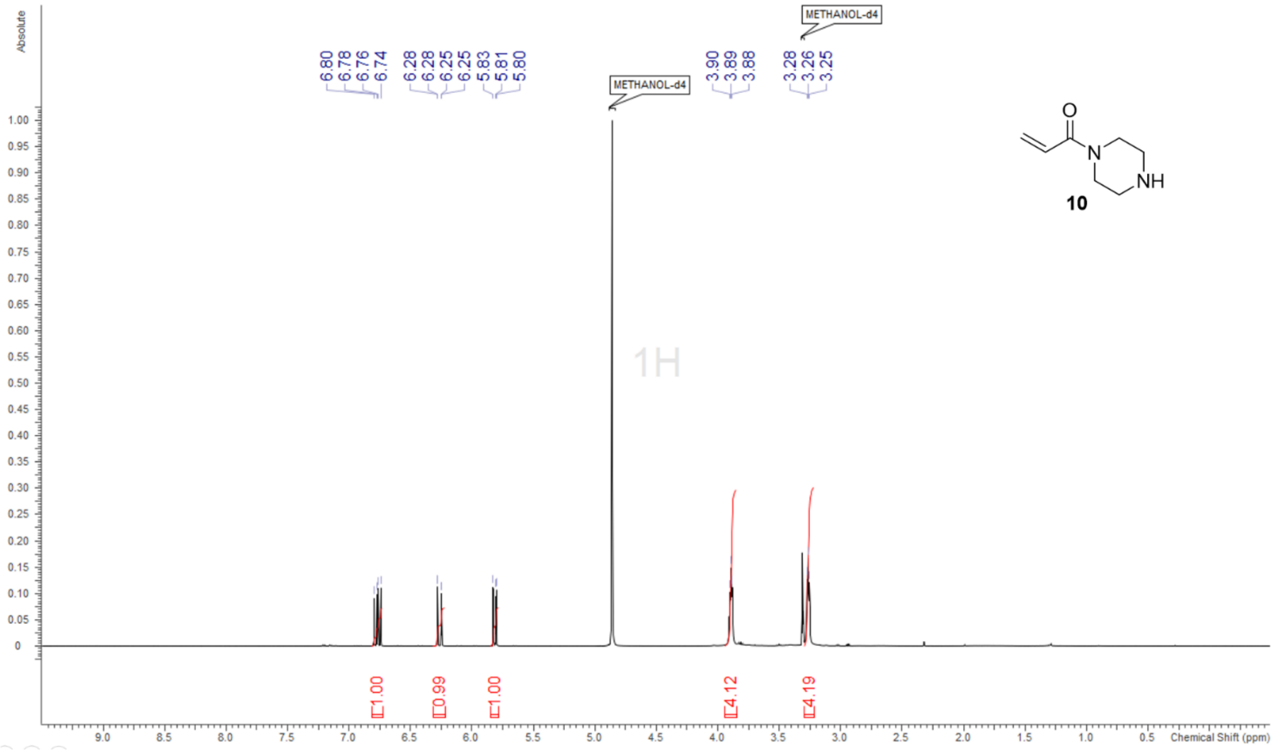

Figure S51. ^{1}H NMR (400 MHz, CD_3OD) of **10**.

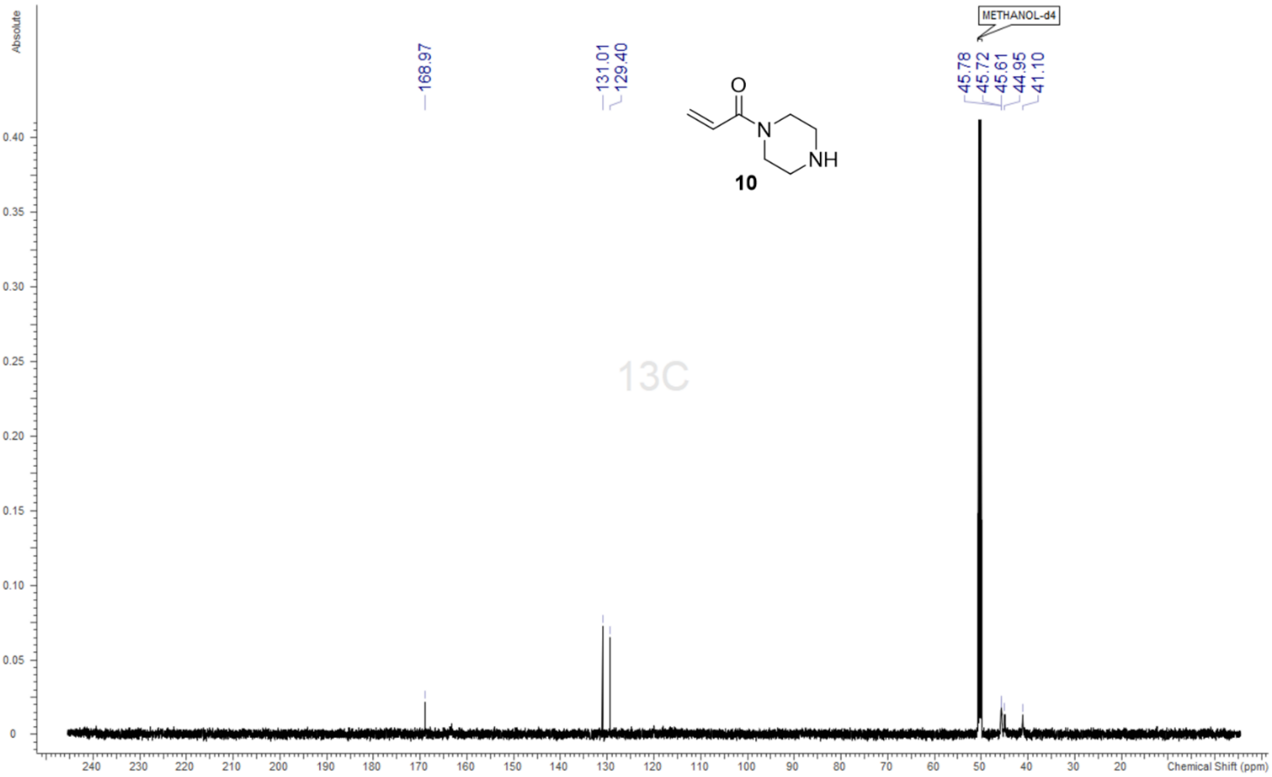

Figure S52 ^{13}C NMR (100 MHz, CD_3OD) of **10**.

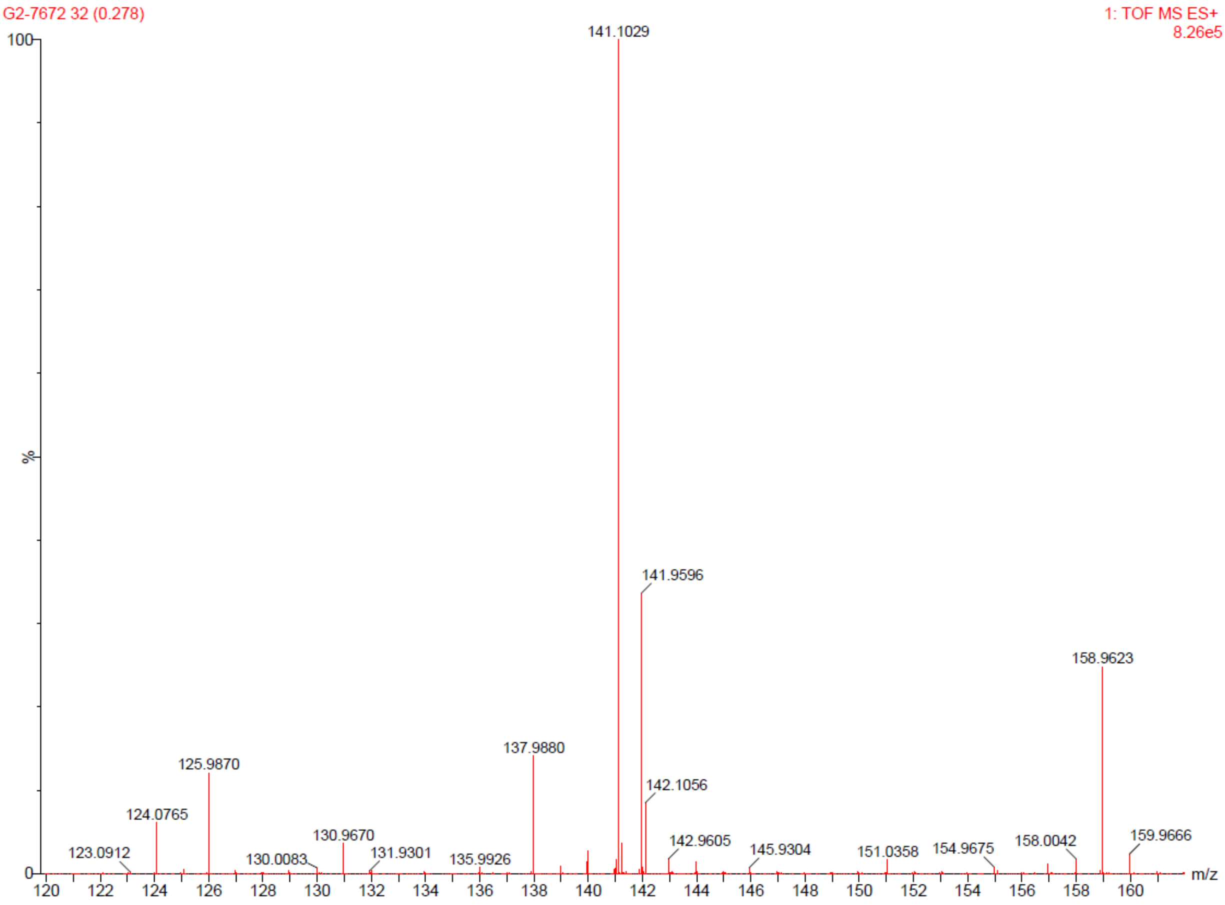

Figure S53. HMRS of **10**.

5.11. Compound 11: 1-(1,4-diazepan-1-yl)prop-2-en-1-one

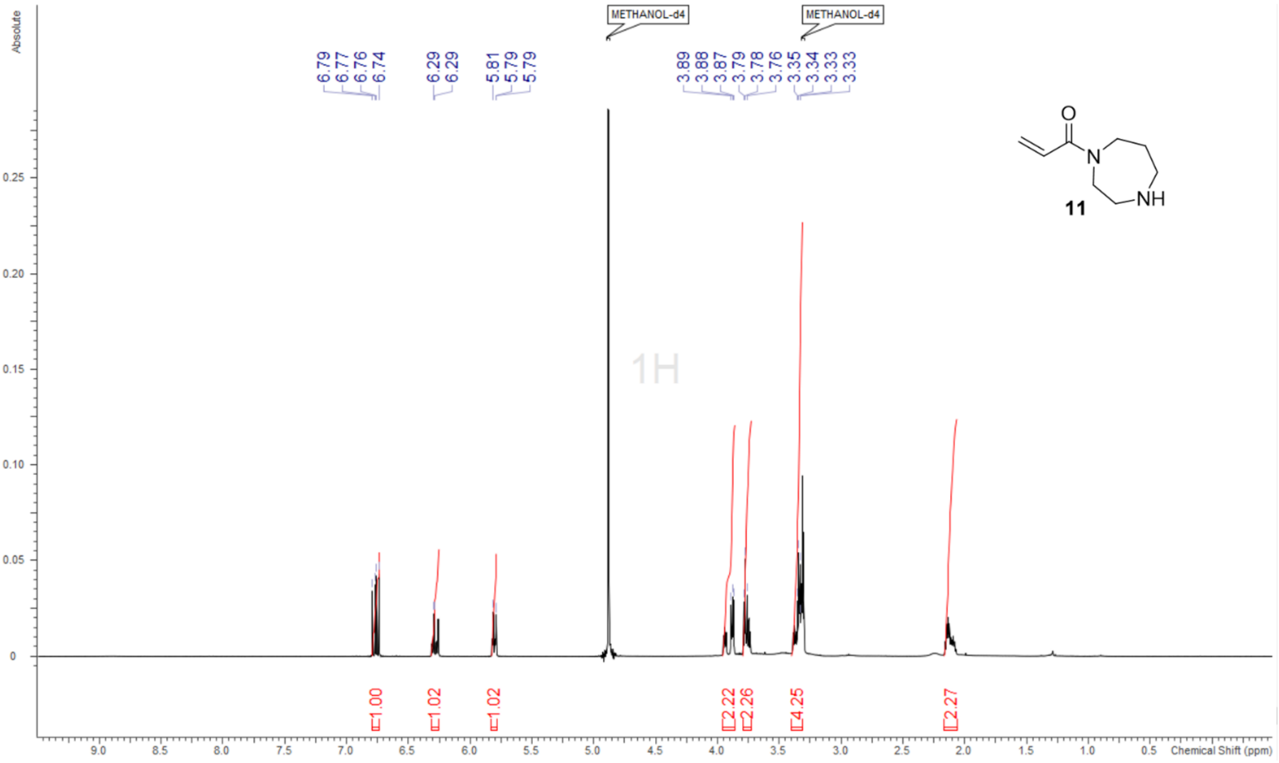

Figure S54. ^{1}H NMR (400 MHz, CD_3OD) of **11**.

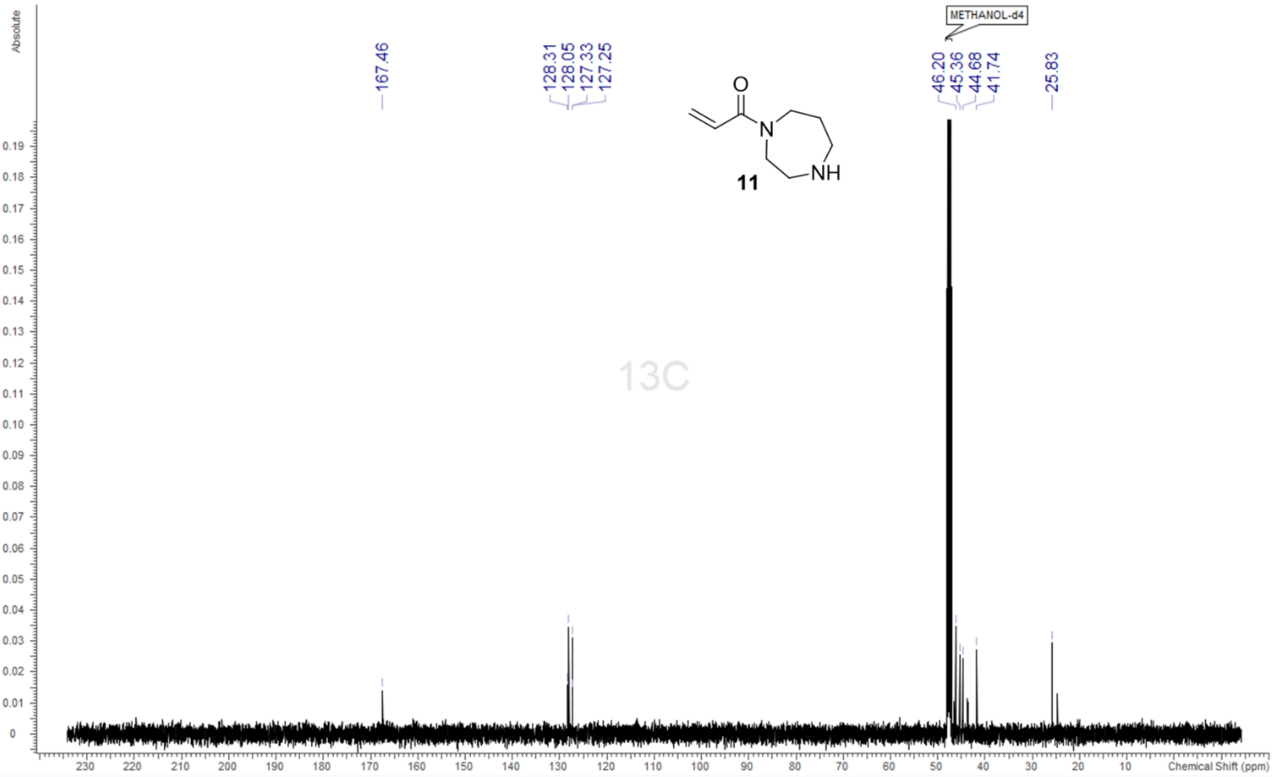

Figure S55 ^{13}C NMR (100 MHz, CD_3OD) of **11**.

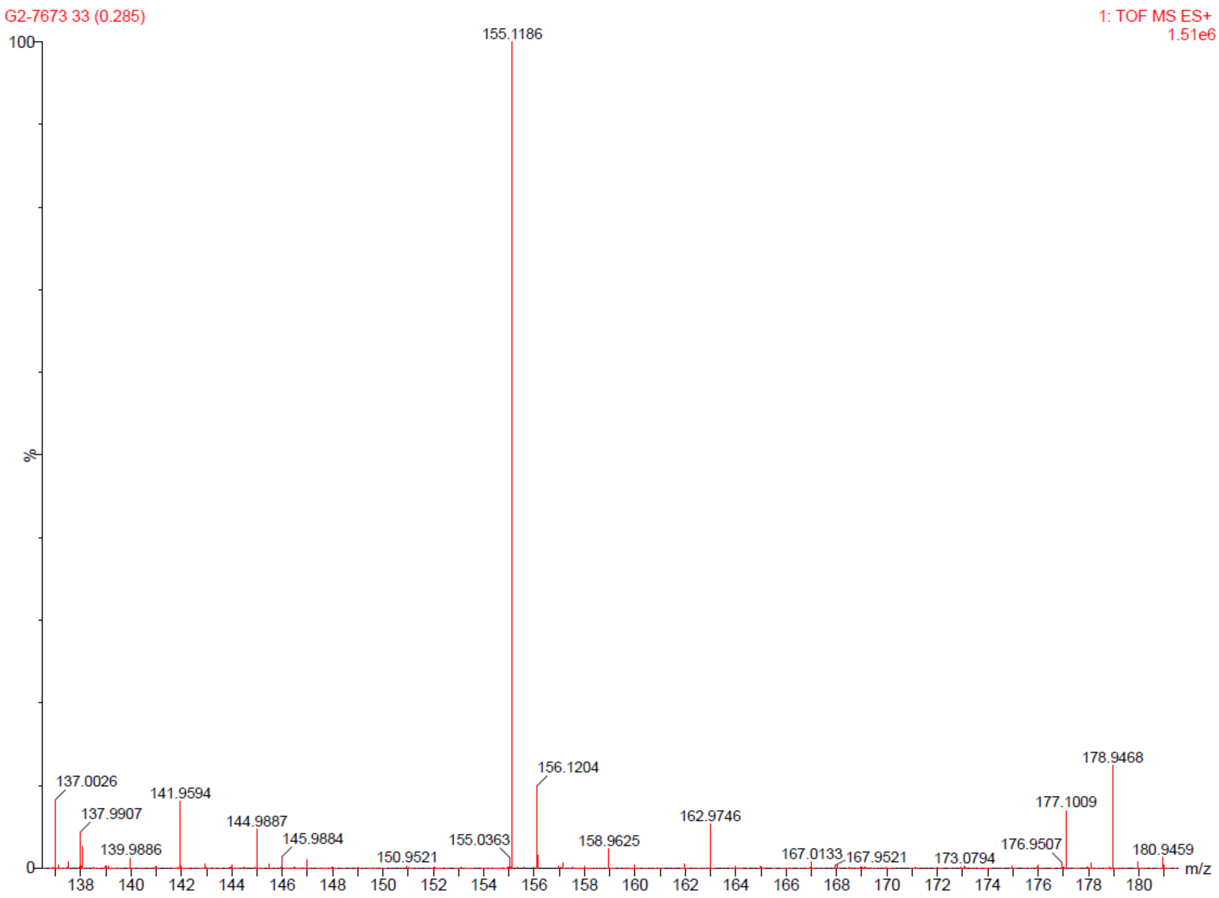

Figure S56. HMRS of **11**.

5.12. Compound 12: *N*-((1*R*,2*R*)-2-aminocyclohexyl)acrylamide

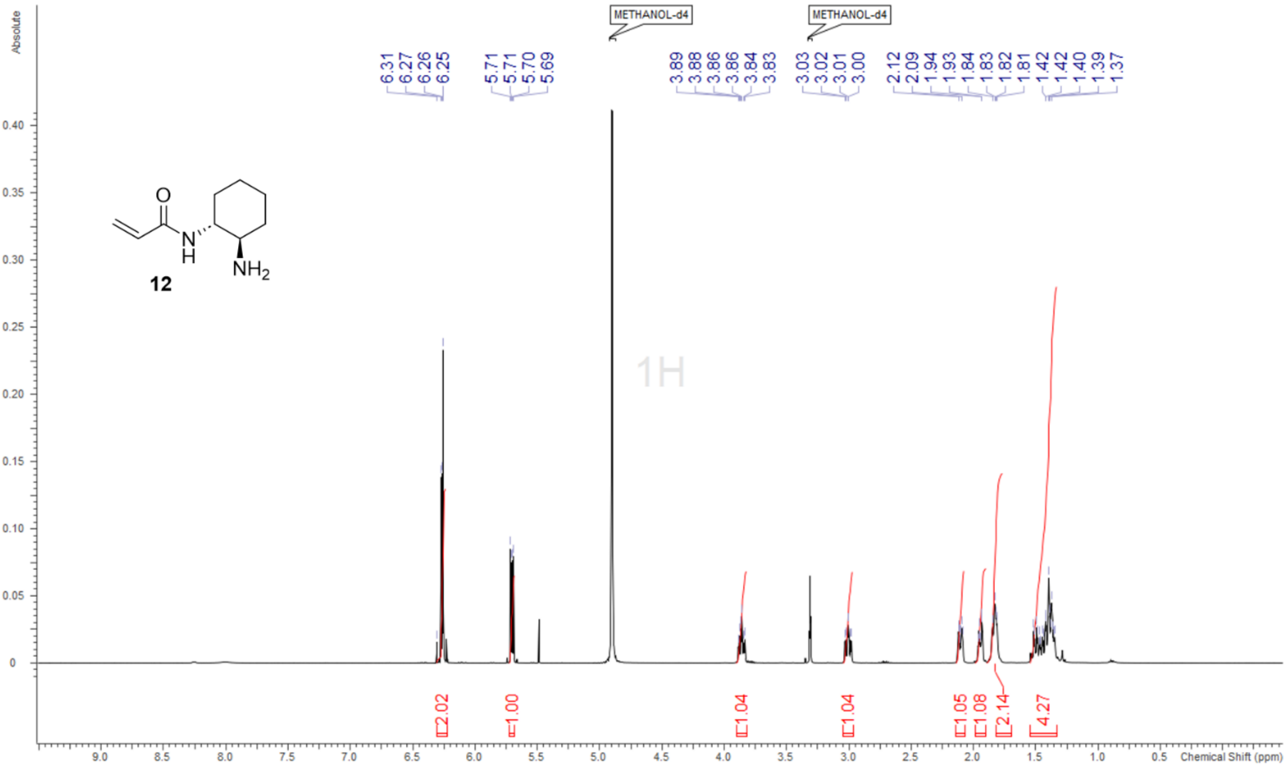

Figure S57. ^{1}H NMR (500 MHz, CD_3OD) of **12**.

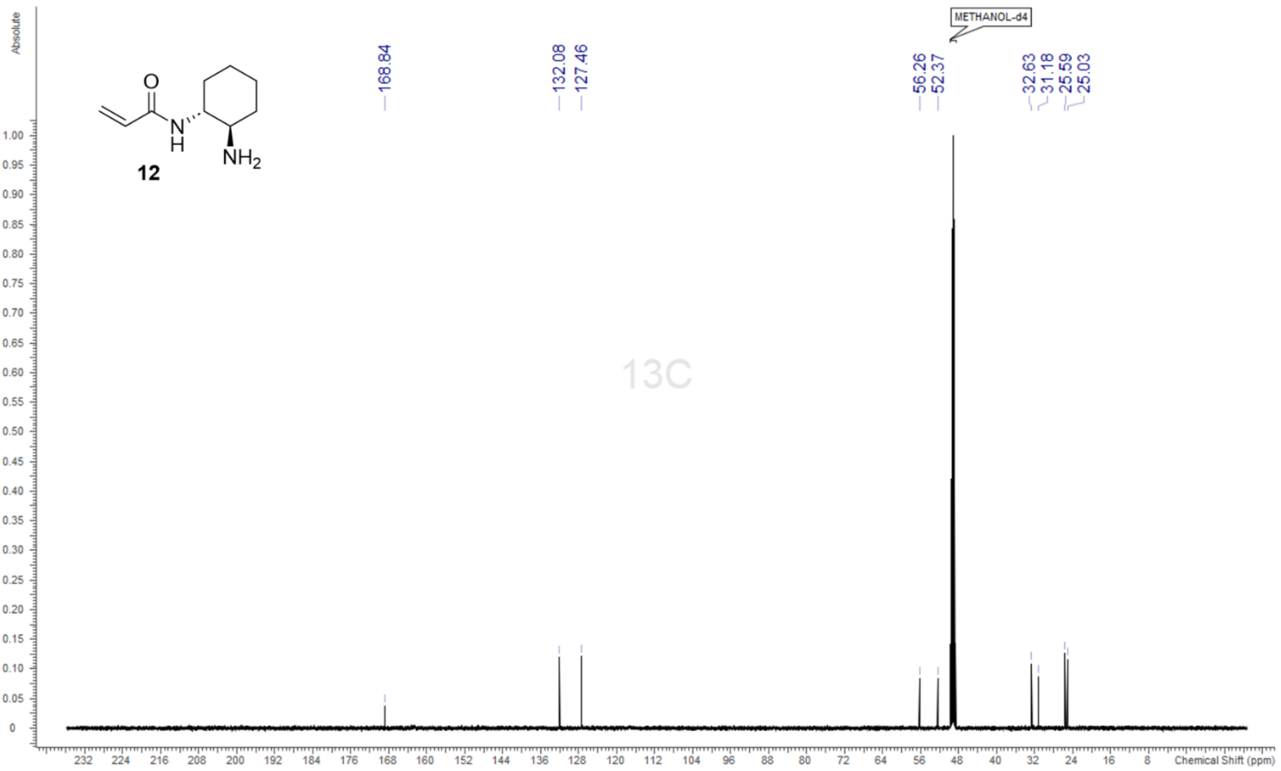

Figure S58 ^{13}C NMR (125 MHz, CD_3OD) of **12**.

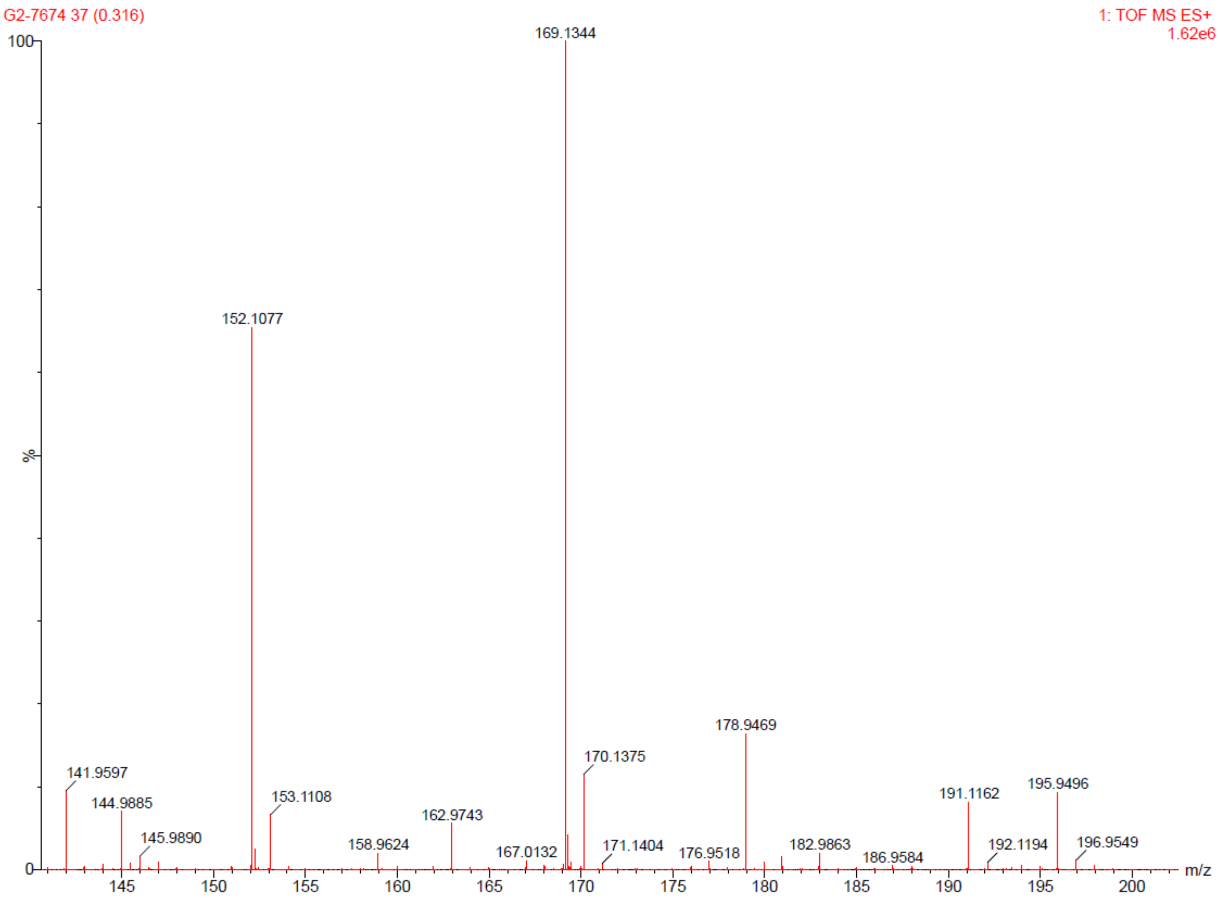

Figure S59. HMRS of **12**.

5.13. Compound 13: *N*-((1*S*,2*S*)-2-aminocyclohexyl)acrylamide

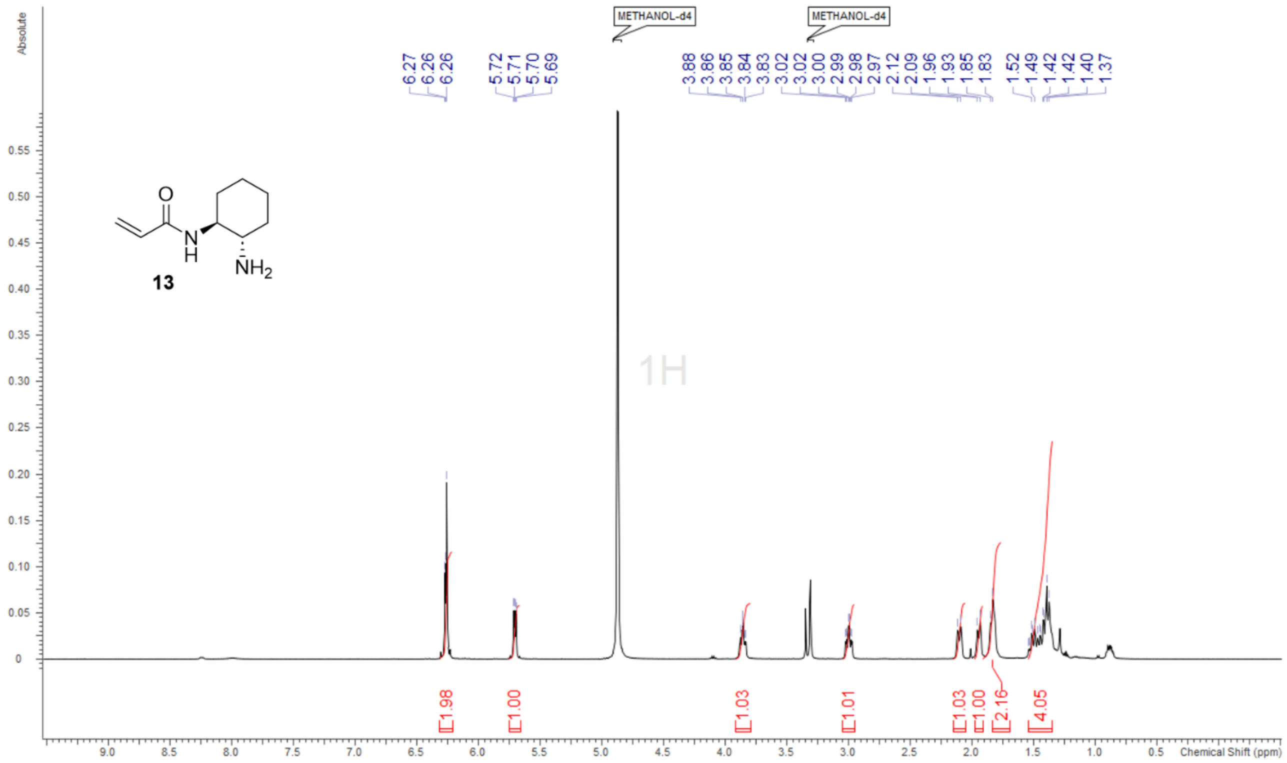

Figure S60. ^{1}H NMR (500 MHz, CD_3OD) of **13**.

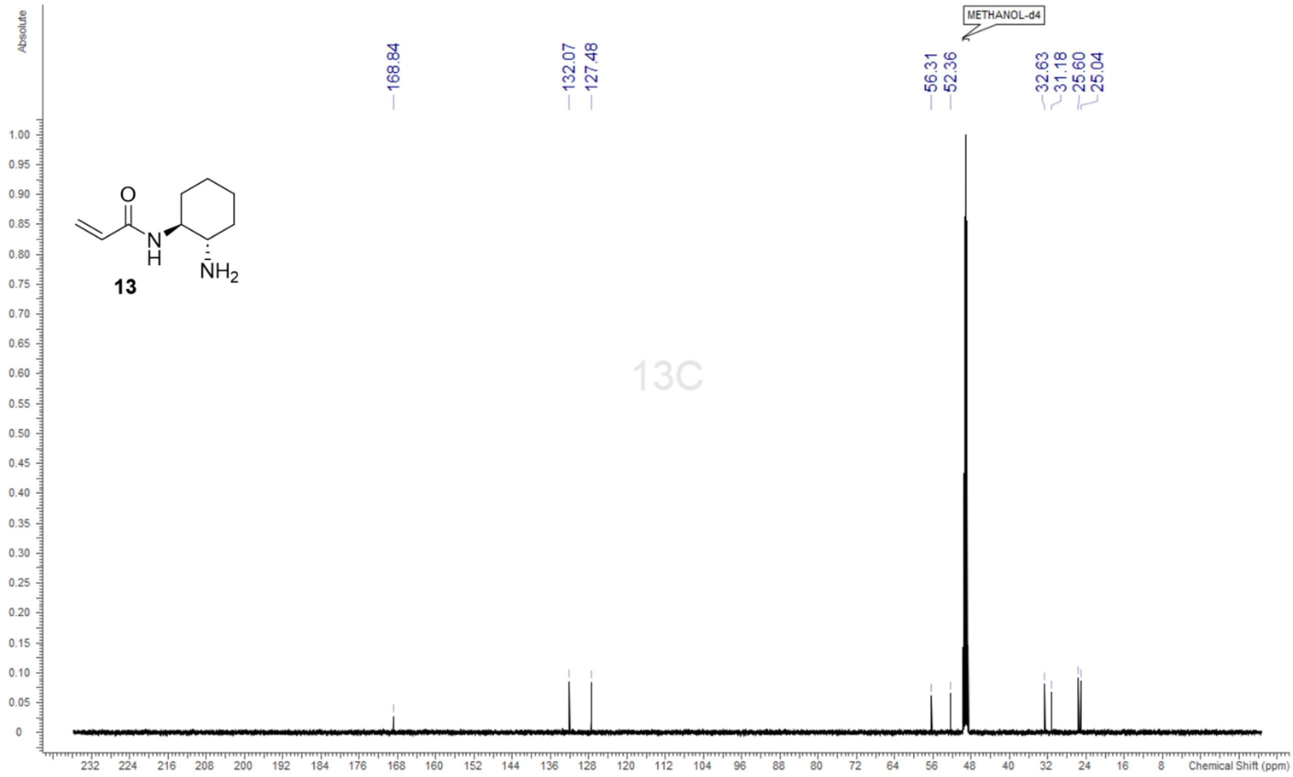

Figure S61 ^{13}C NMR (125 MHz, CD_3OD) of **13**.

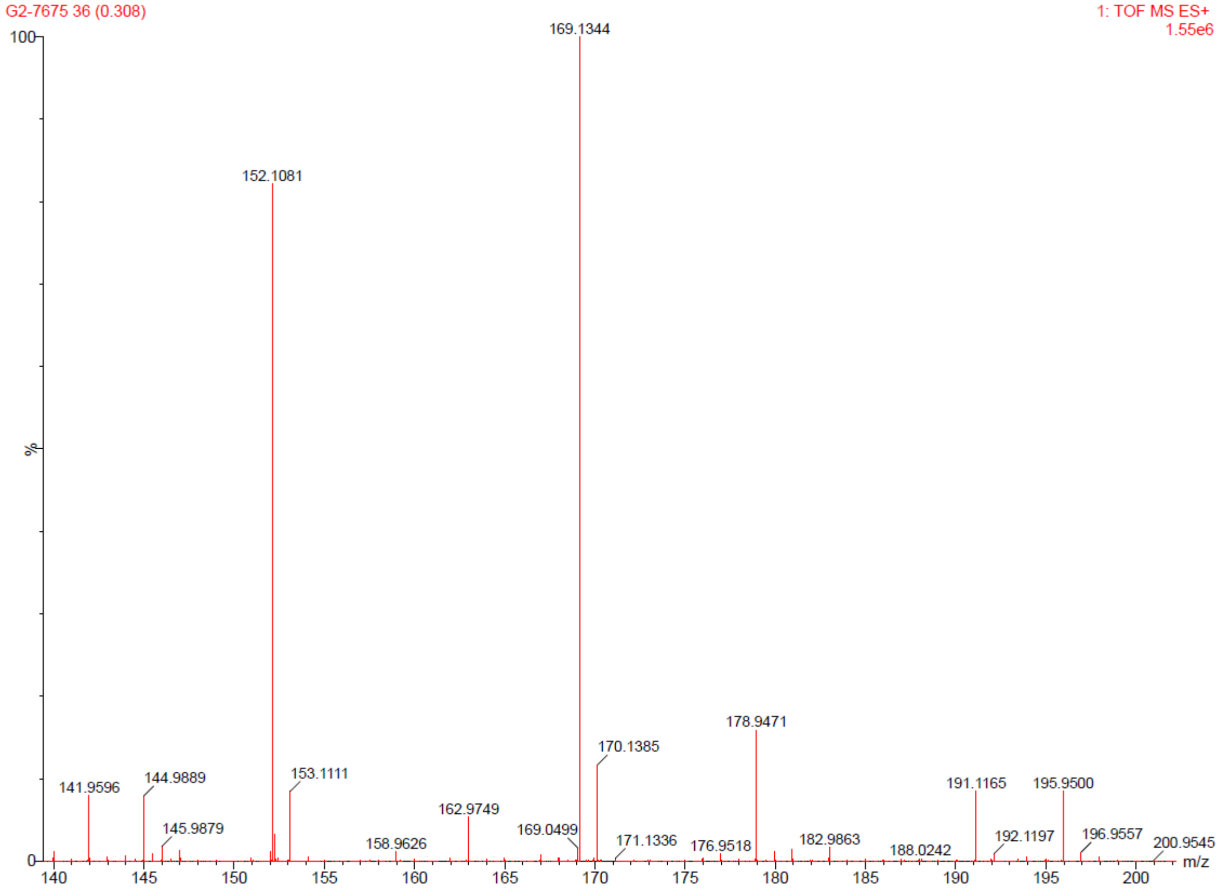

Figure S62. HMRS of **13**.

5.14. Compound 14: *N*-(2-guanidinoethyl)acrylamide

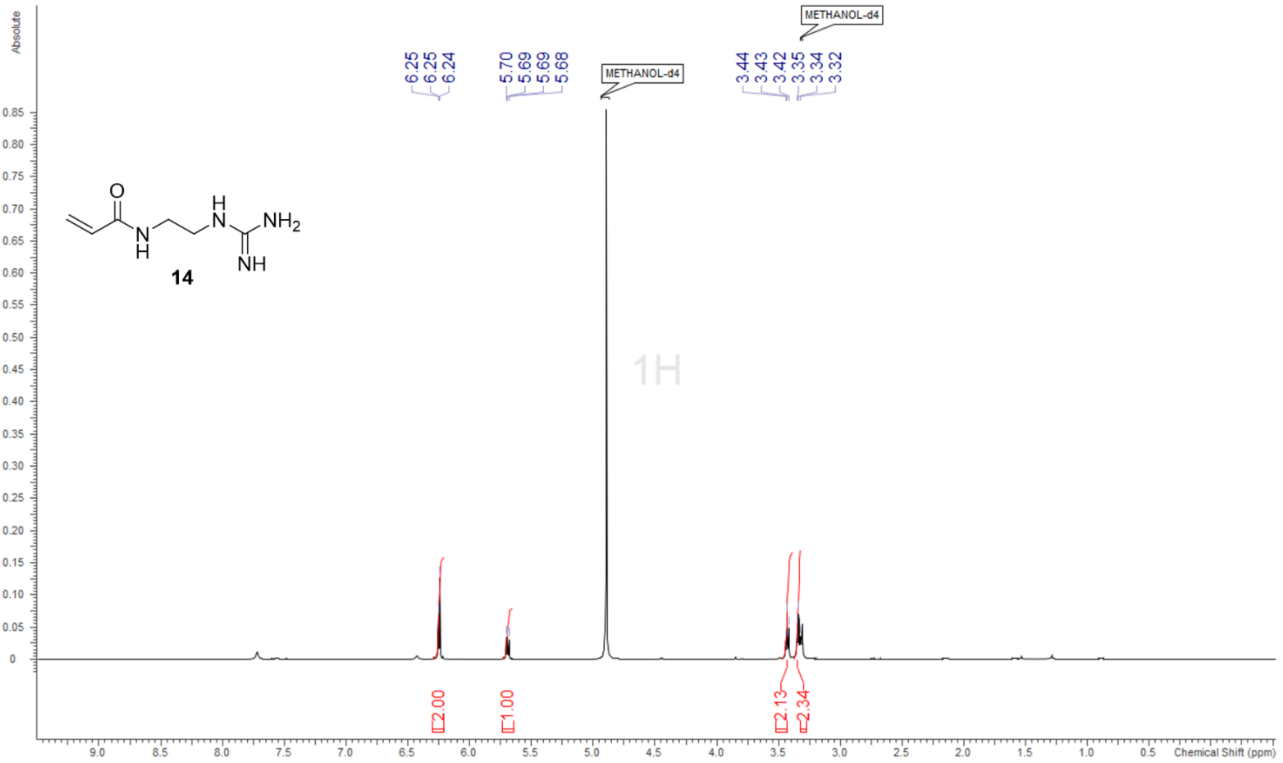

Figure S63. ^{1}H NMR (400 MHz, CD_3OD) of **14**.

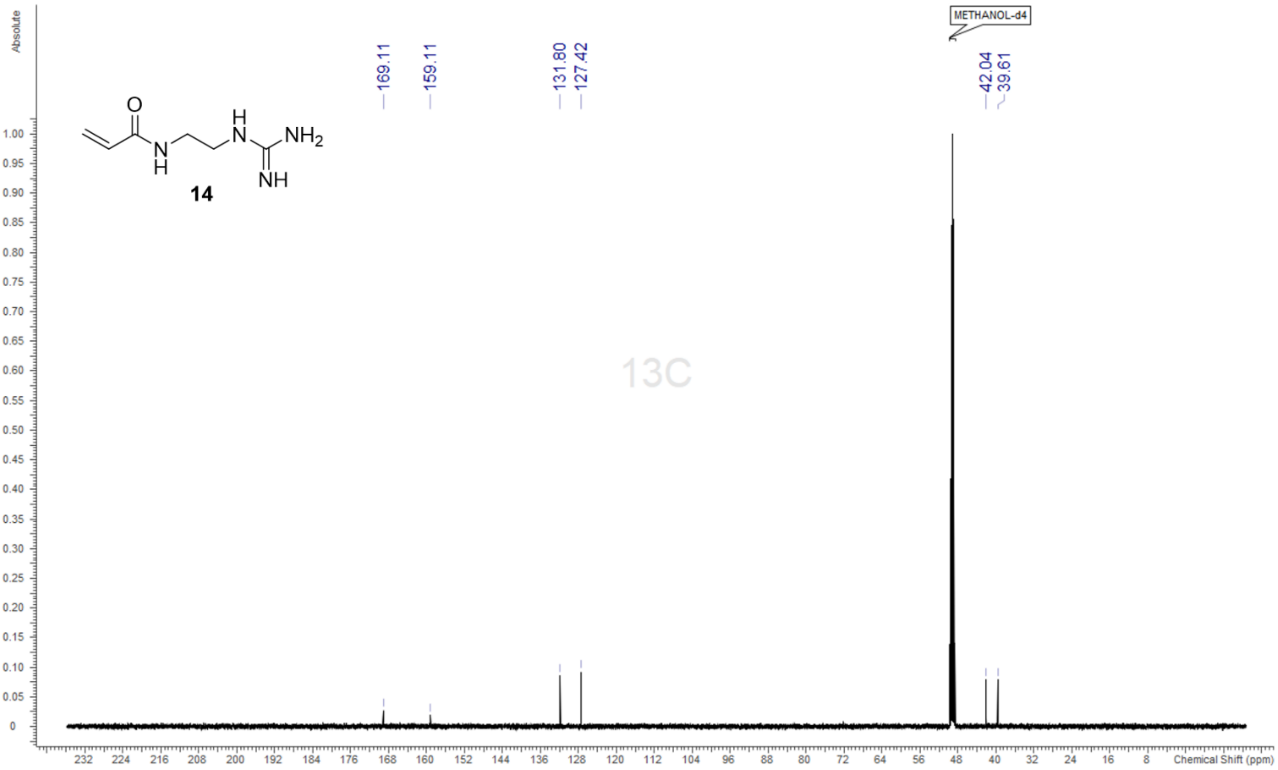

Figure S64 ^{13}C NMR (100 MHz, CD_3OD) of **14**.

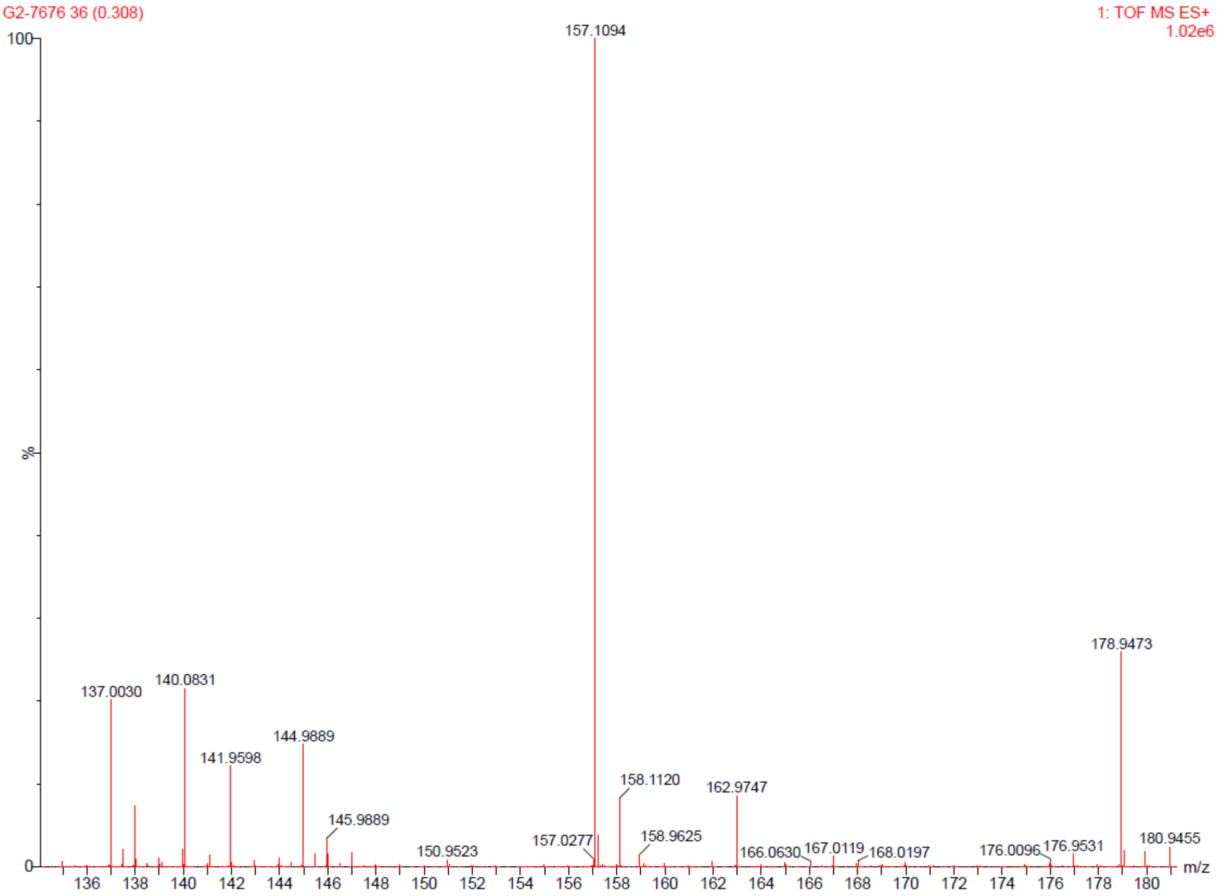

Figure S65. HMRS of **14**.

5.15. Compound 15: *N*-(2-(piperidin-1-yl)ethyl)acrylamide

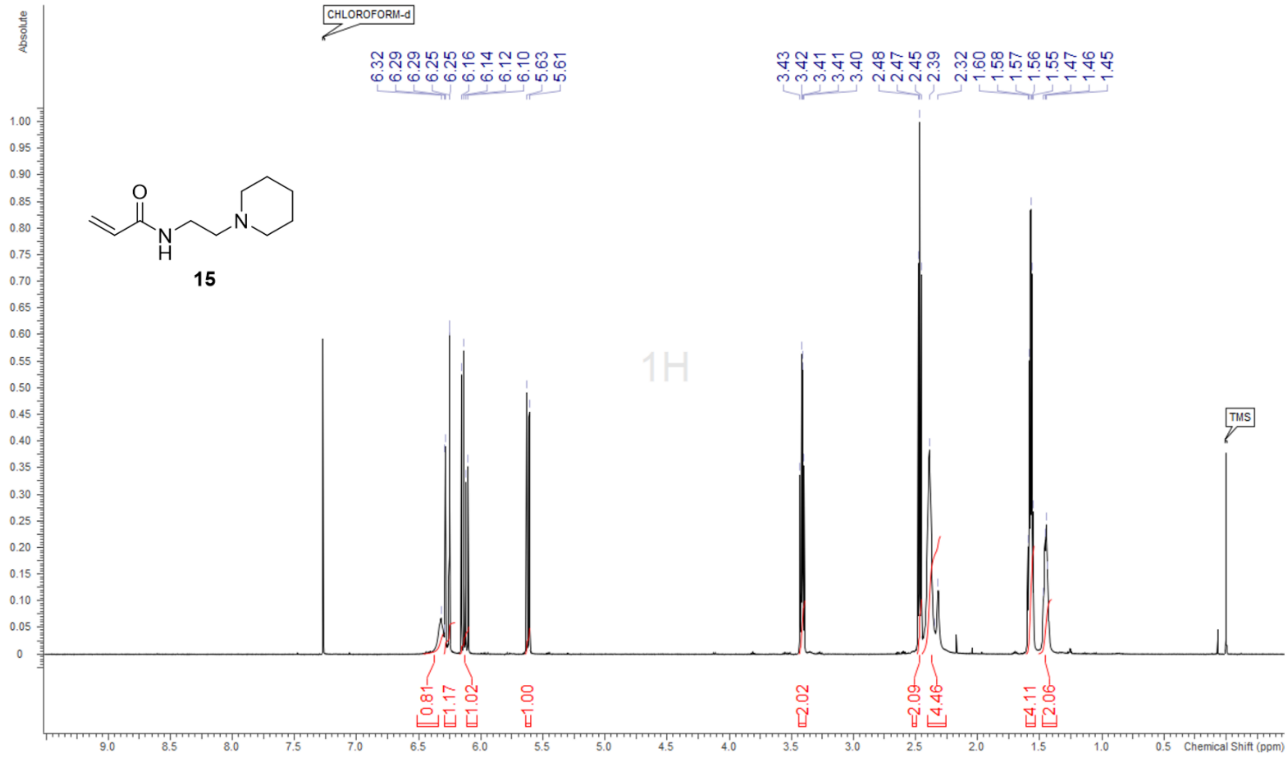

Figure S66. ^{1}H NMR (500 MHz, $CDCl_3$) of **15**.

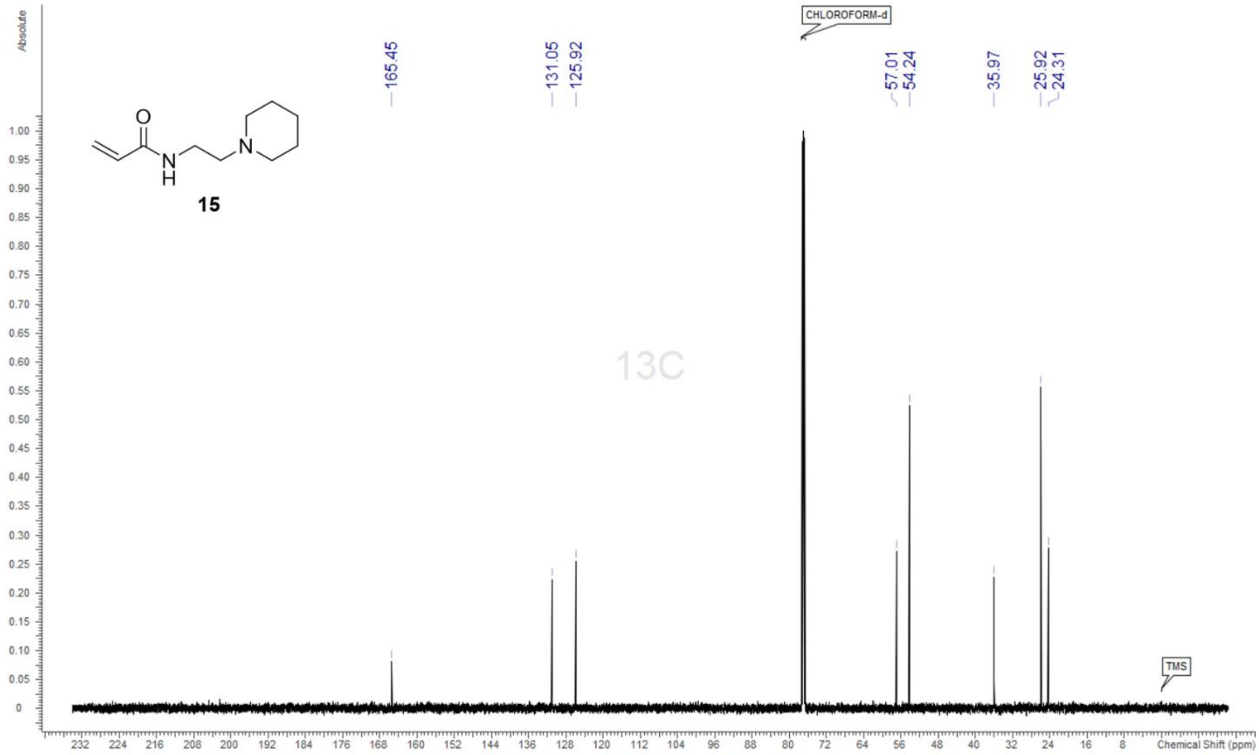

Figure S67. ^{13}C NMR (125 MHz, $CDCl_3$) of **15**.

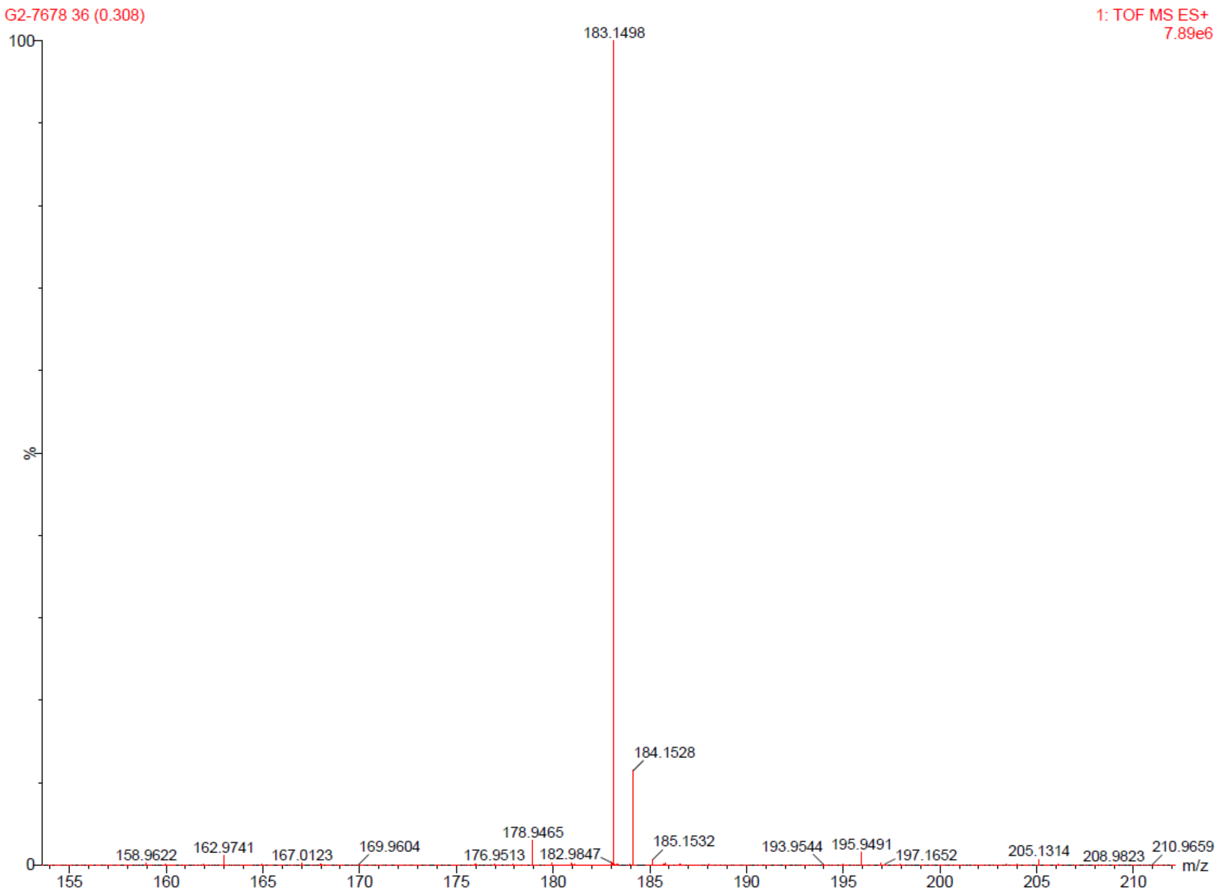

Figure S68. HMRS of **15**.

5.16. Compound 16: *N*-(2-hydroxyethyl)acrylamide

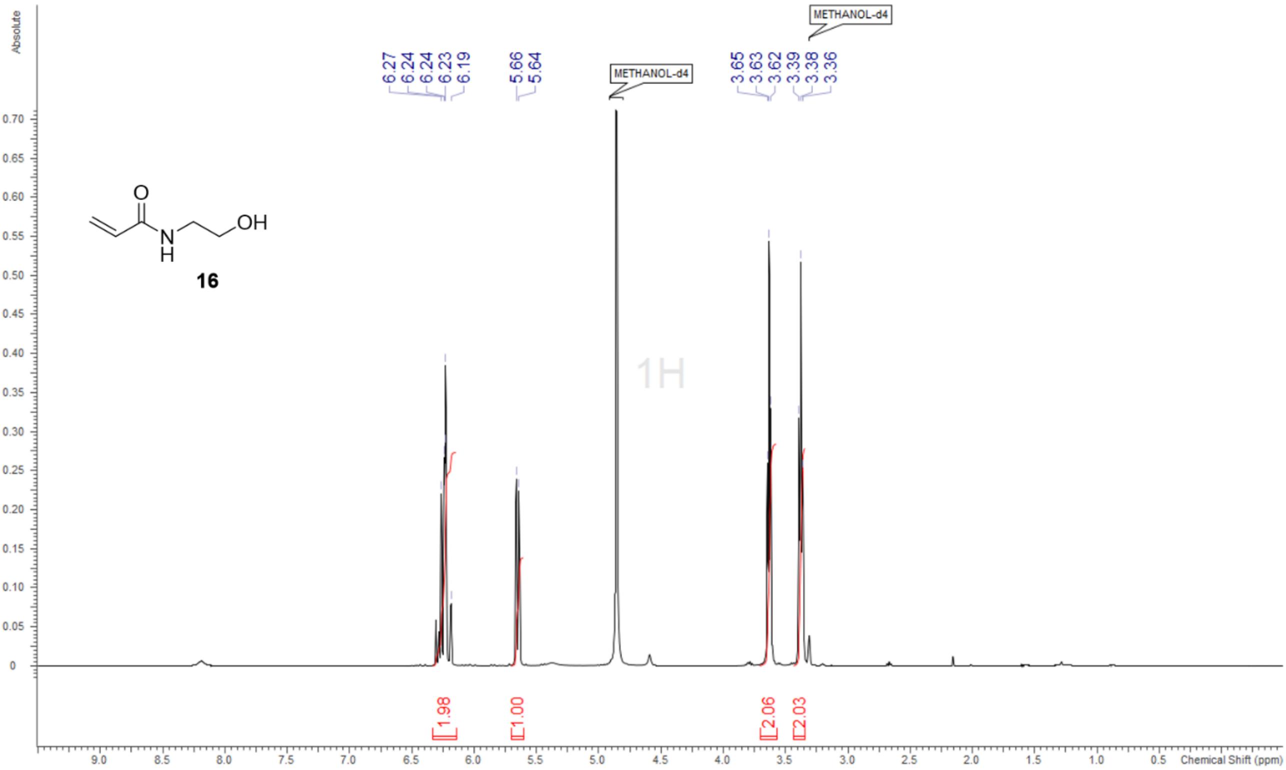

Figure S69. ^{1}H NMR (400 MHz, CD_3OD) of **16**.

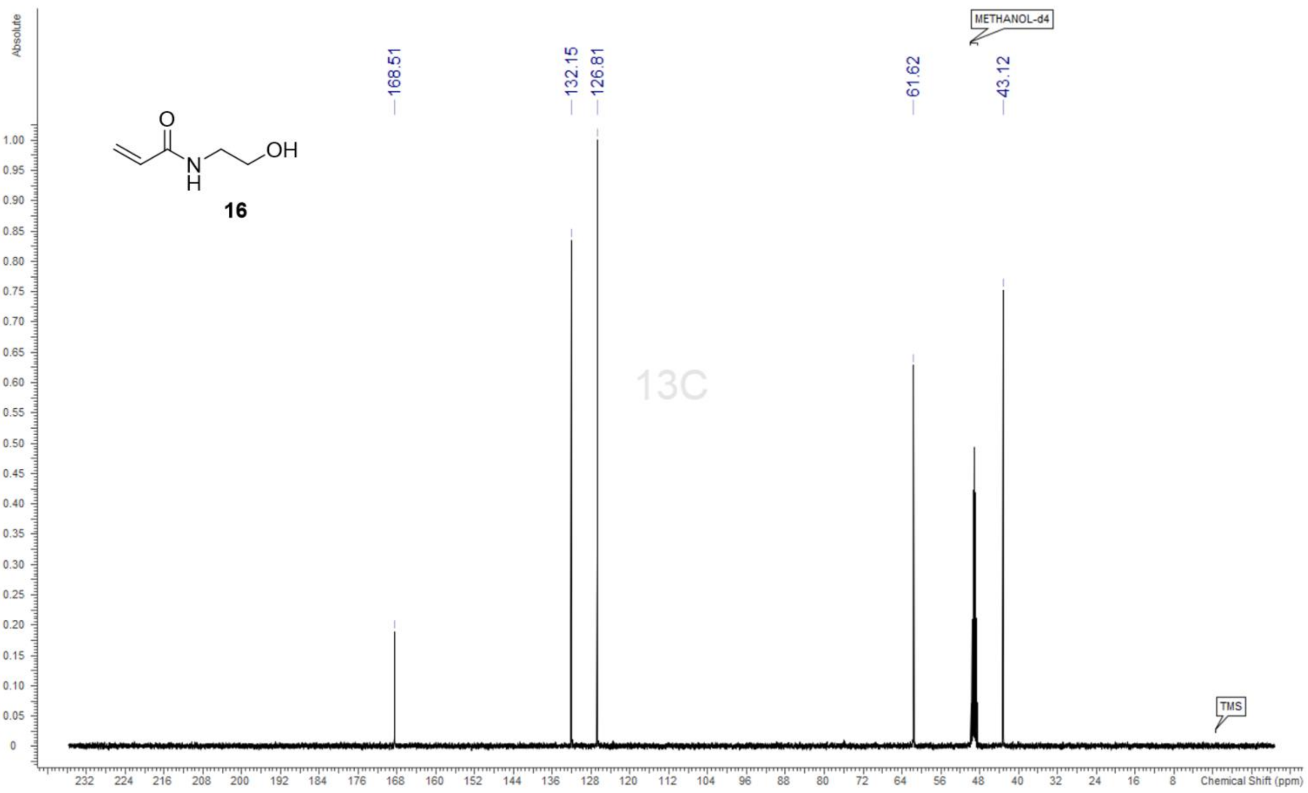

Figure S70. ^{13}C NMR (100 MHz, CD_3OD) of **16**.

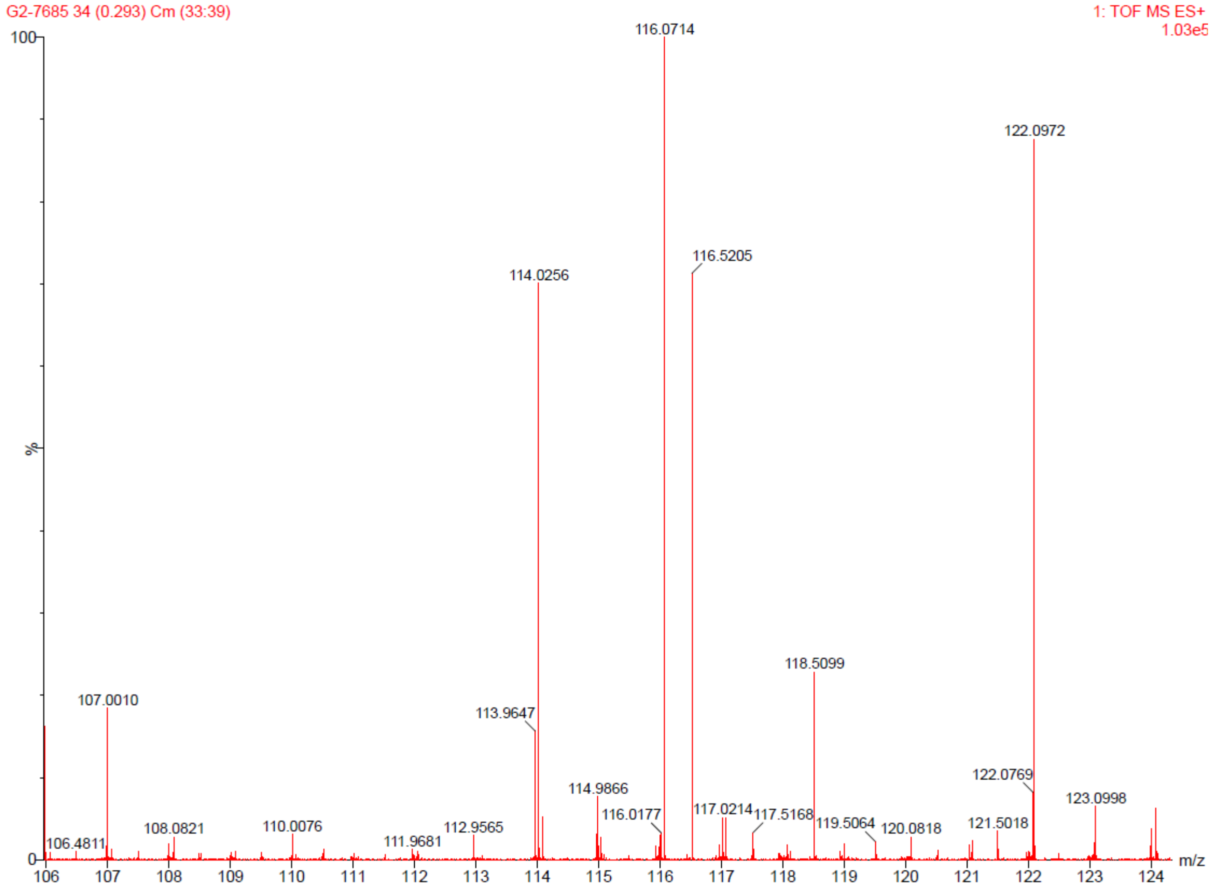

Figure S71. HMRS of **16**.

5.17. Compound 17: *N*-acryloylglycine

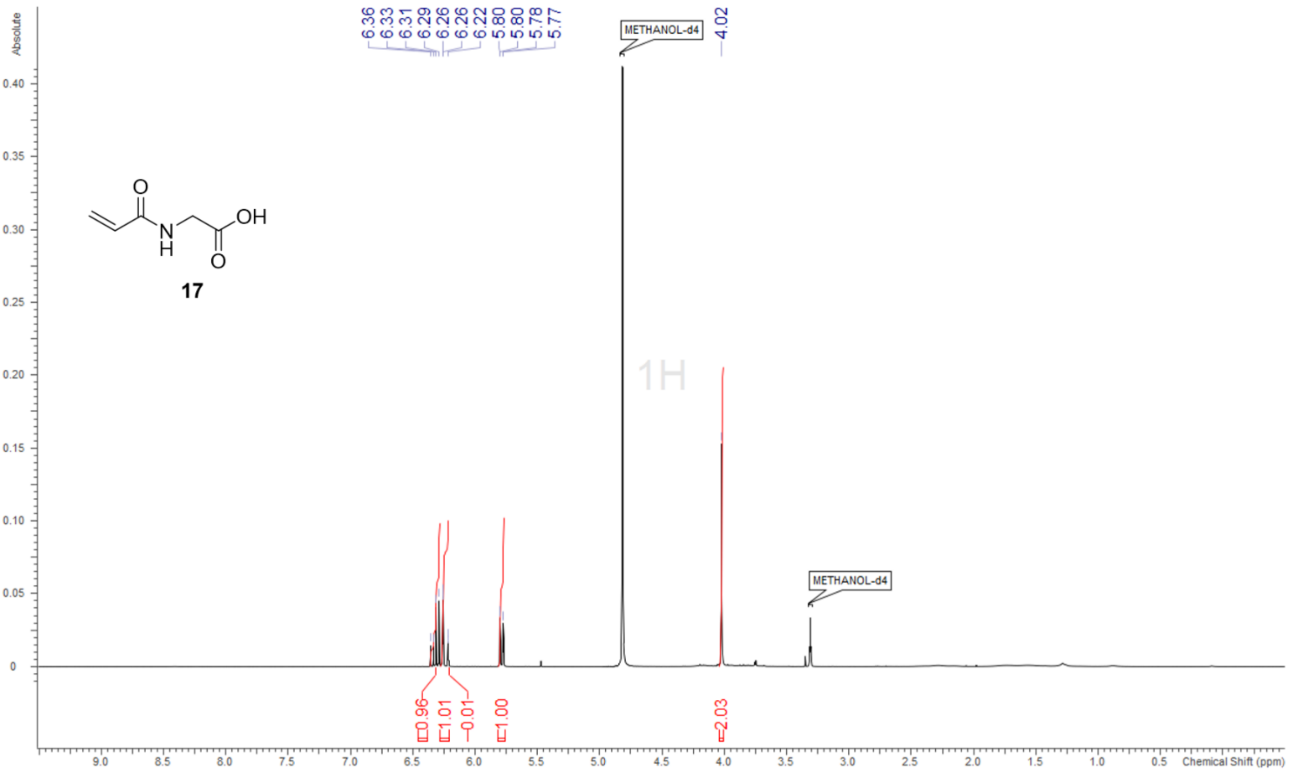

Figure S72. ^{1}H NMR (400 MHz, CD_3OD) of **17**.

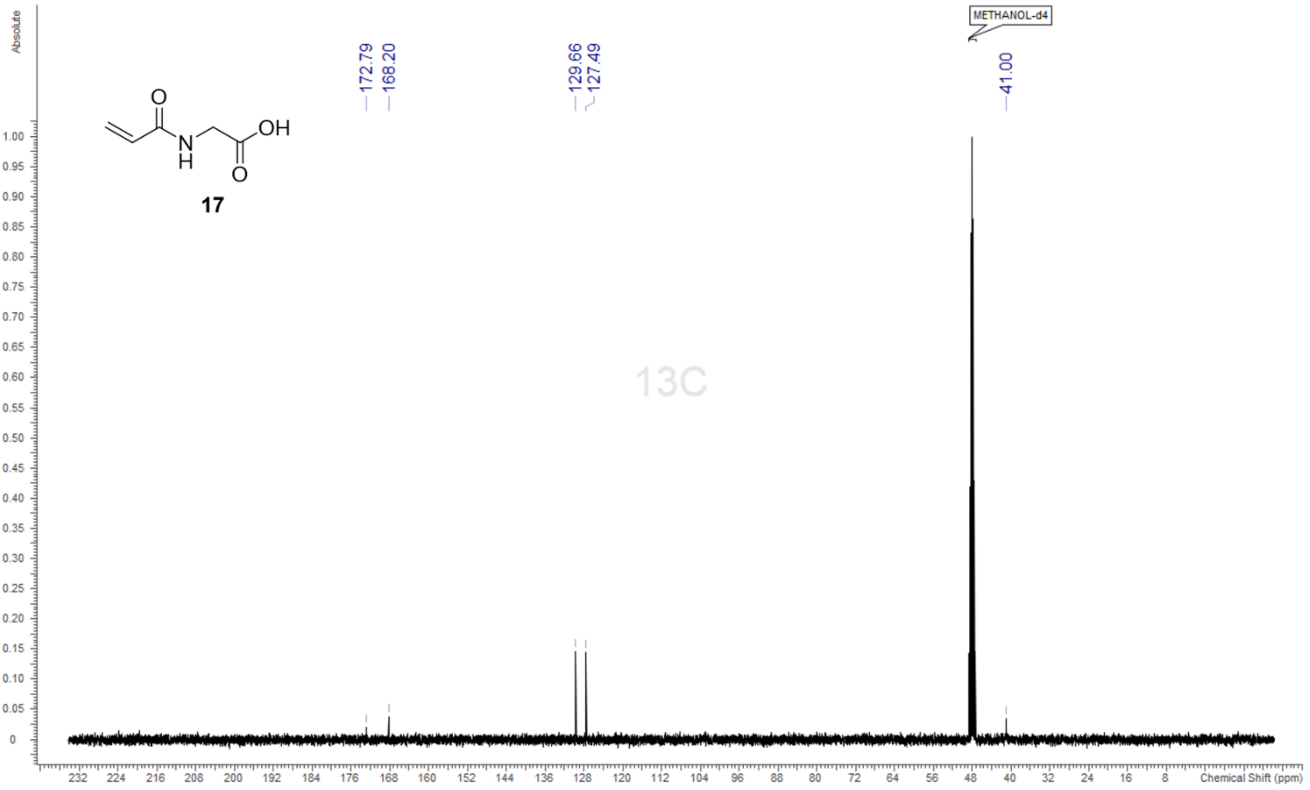

Figure S73. ^{13}C NMR (100 MHz, CD_3OD) of **17**.

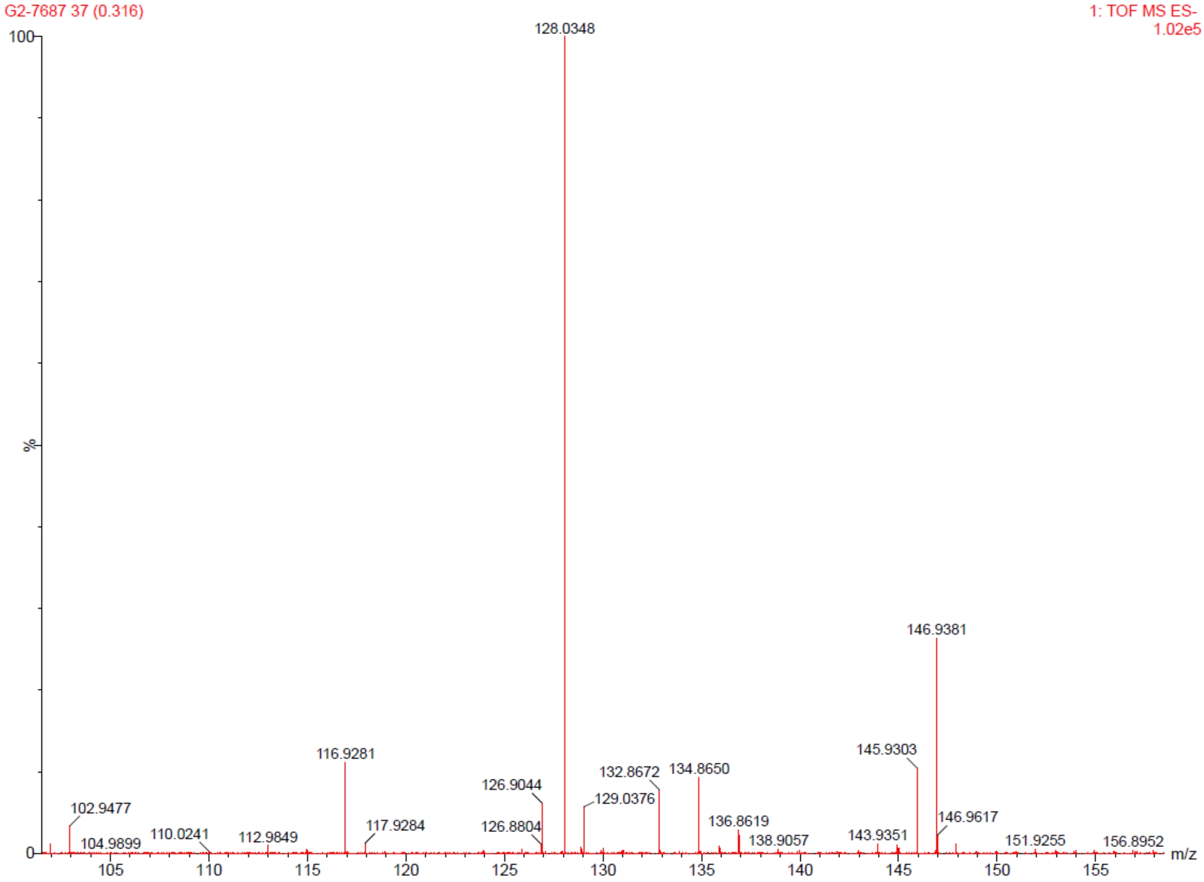

Figure S74. HMRS of **17**.

5.18. Compound 18: *N*-propylacrylamide

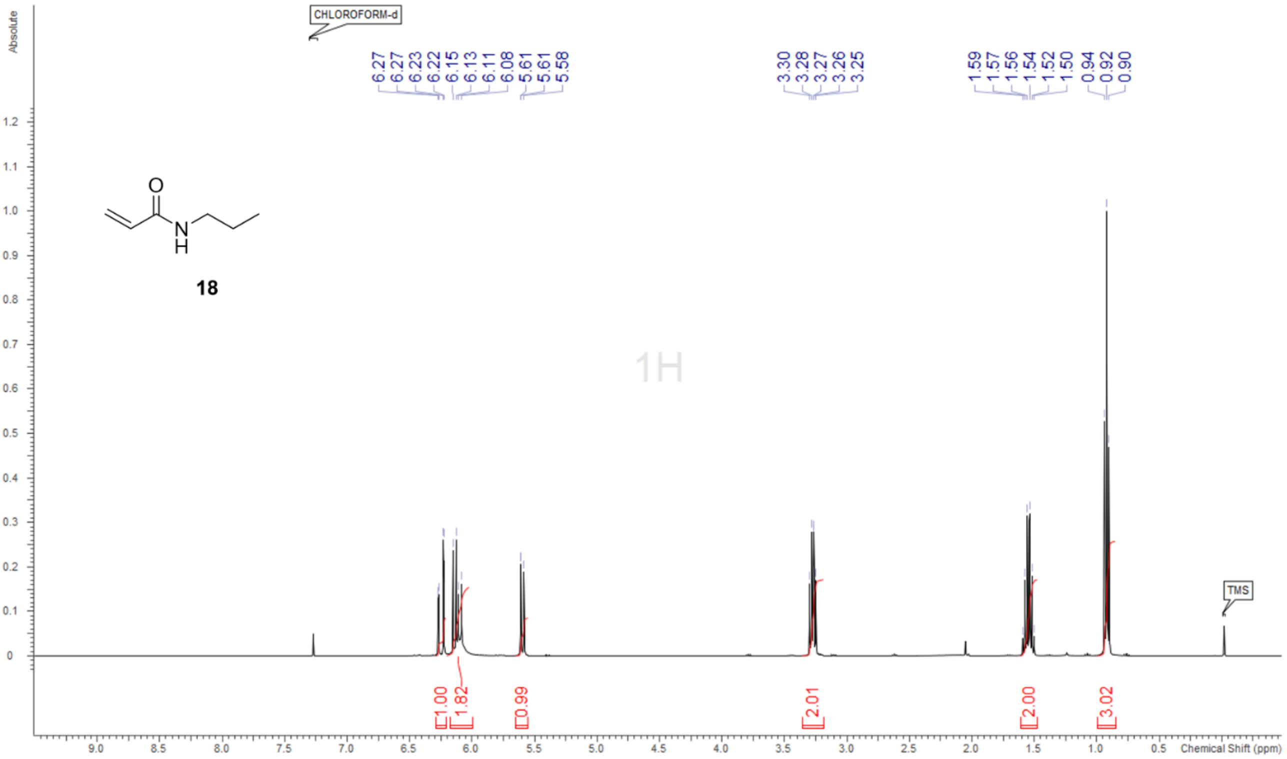

Figure S75. ^{1}H NMR (400 MHz, $CDCl_3$) of **18**.

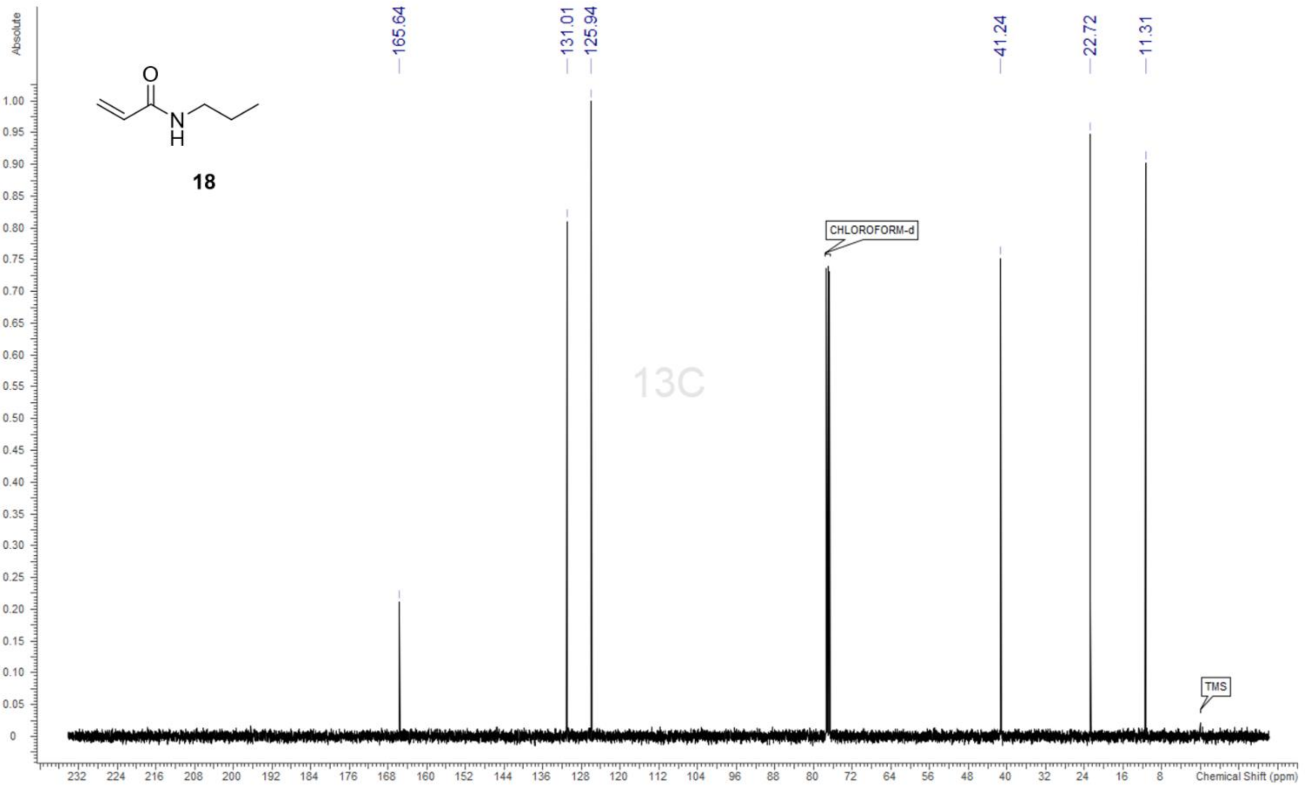

Figure S76 ^{13}C NMR (100 MHz, $CDCl_3$) of **18**.

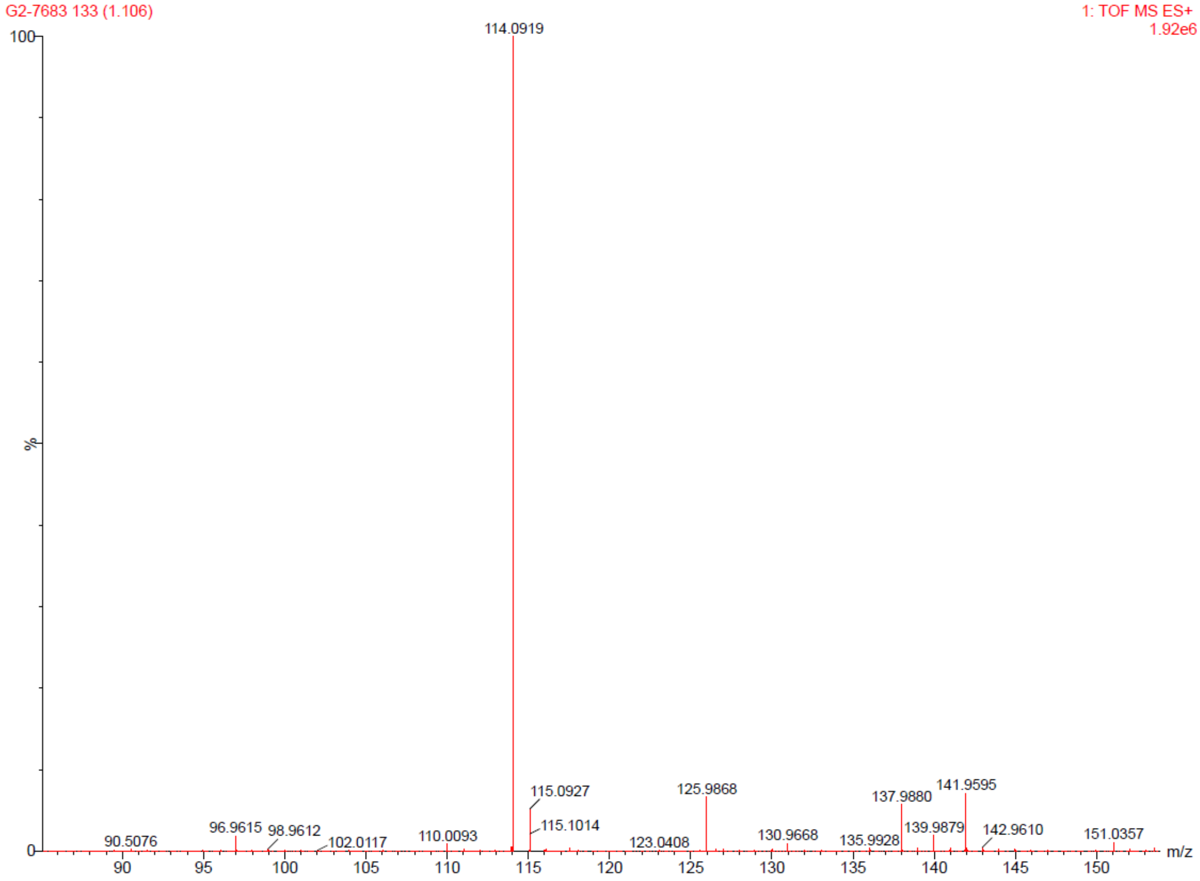

Figure S77. HMRS of **18**.

5.19. Compound 19: 2-aminoethyl acrylate

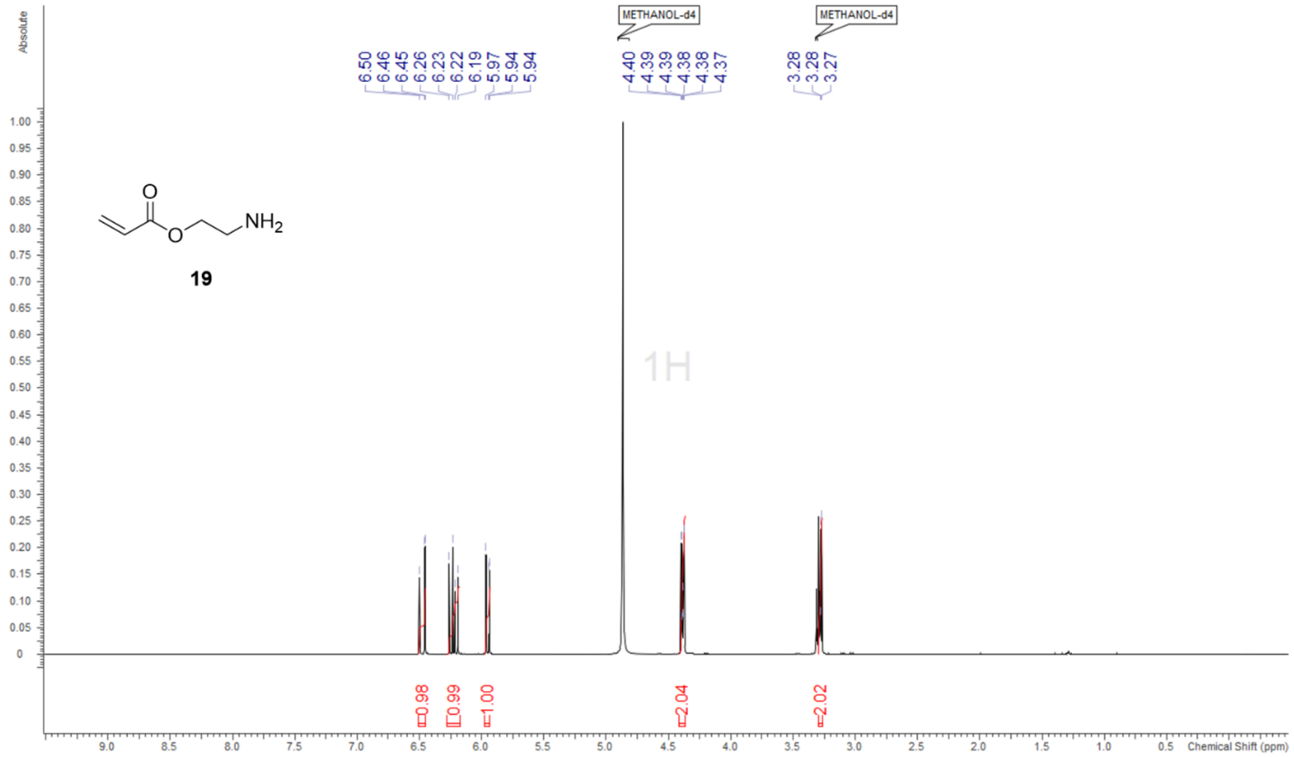

Figure S78. ^{1}H NMR (400 MHz, CD_3OD) of **19**.

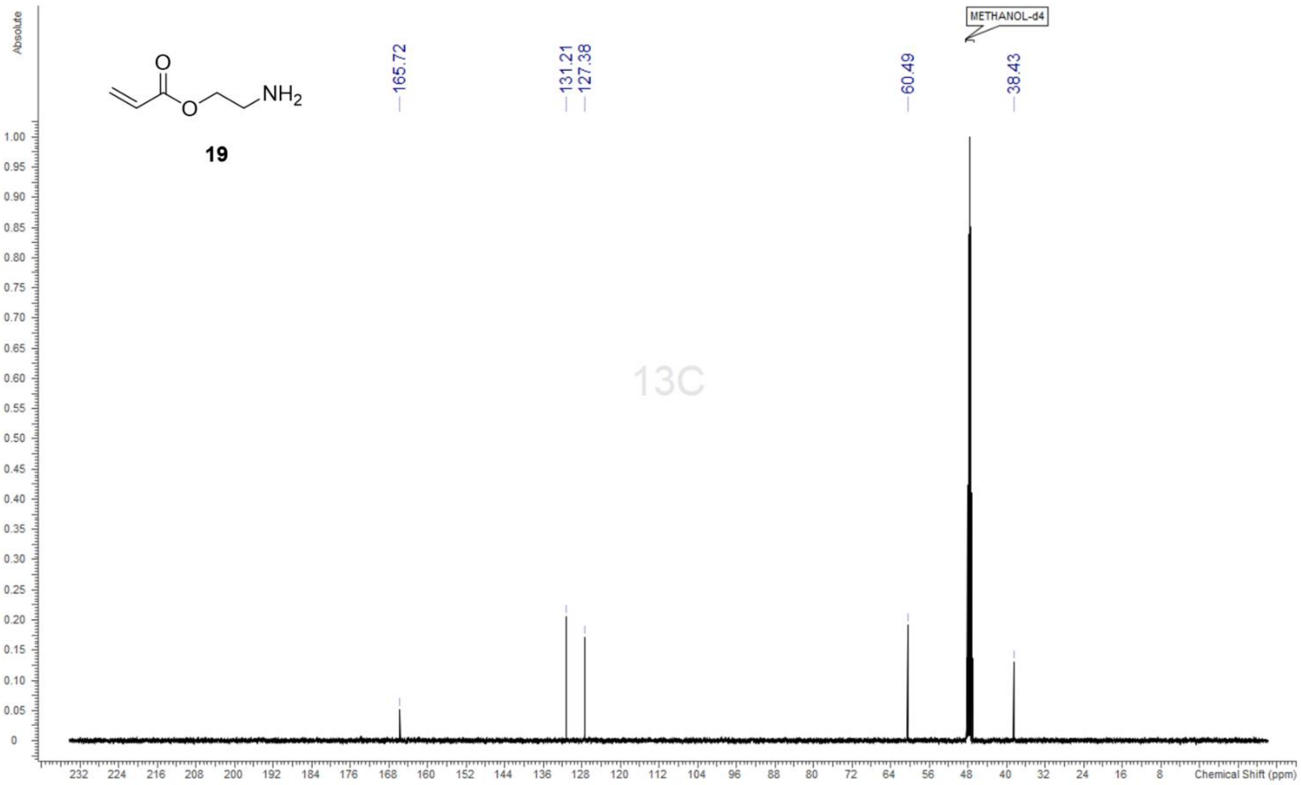

Figure S79. ^{13}C NMR (100 MHz, CD_3OD) of **19**.

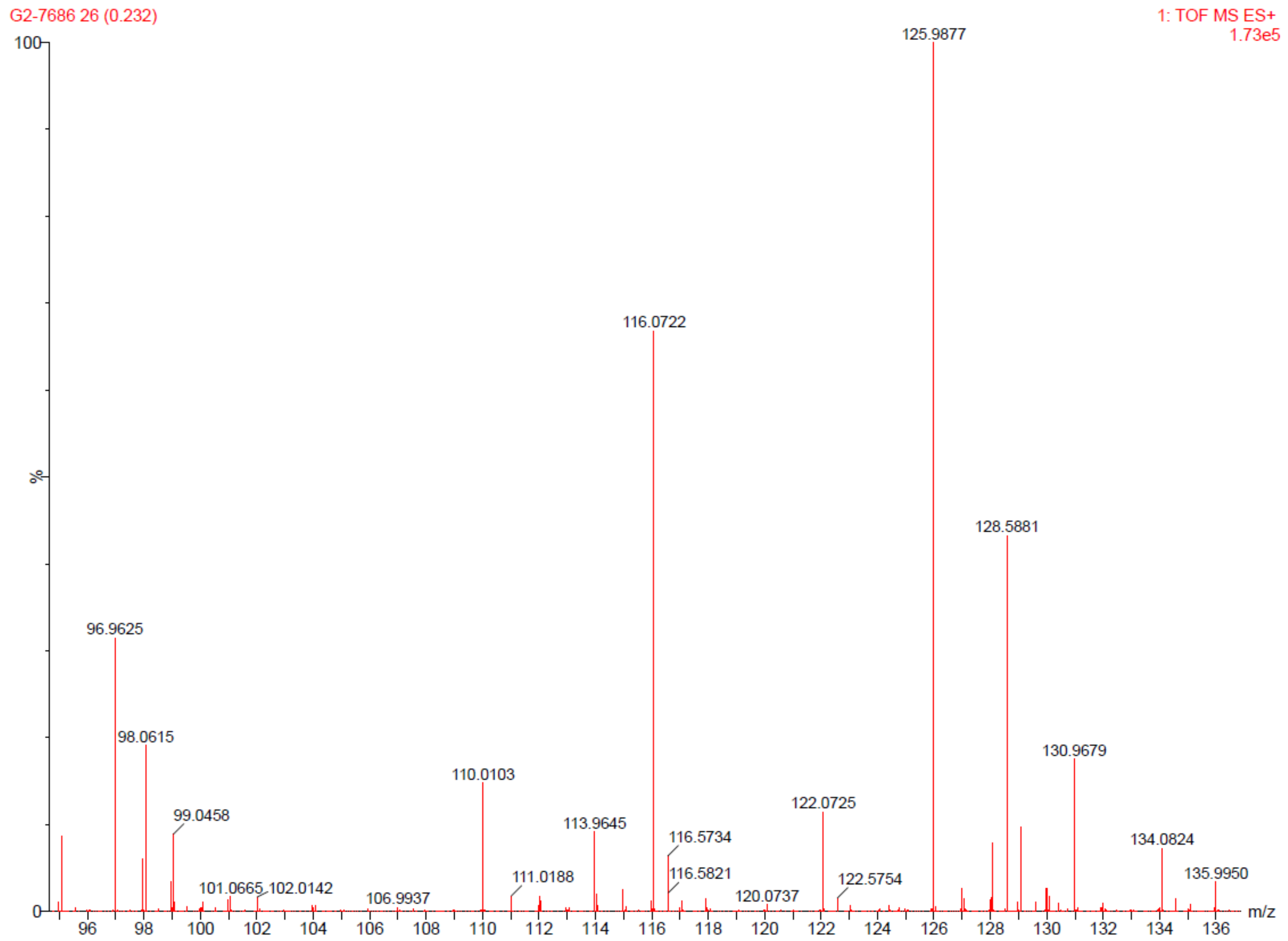

Figure S80. HMRS of **19**.

5.20. Compound 20: *N*-(2-aminoethyl)cinnamamide

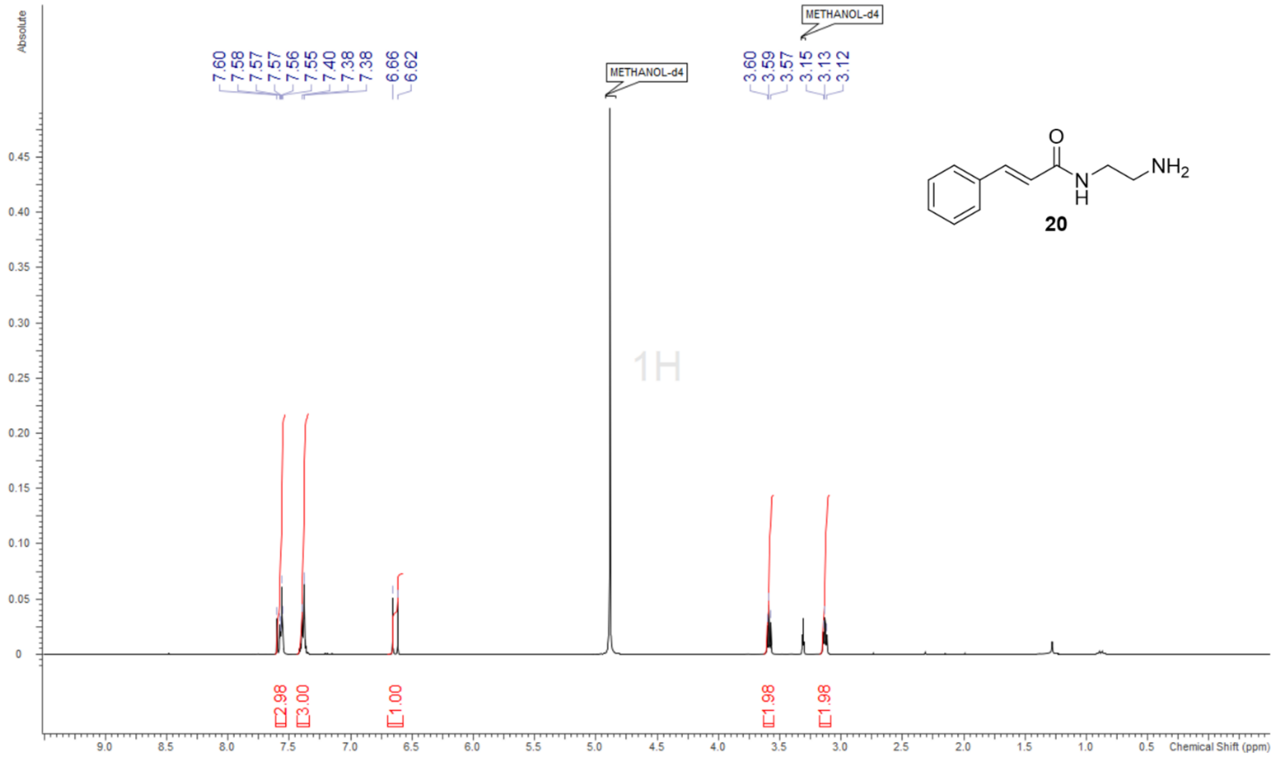

Figure S81. ^{1}H NMR (400 MHz, CD_3OD) of **20**.

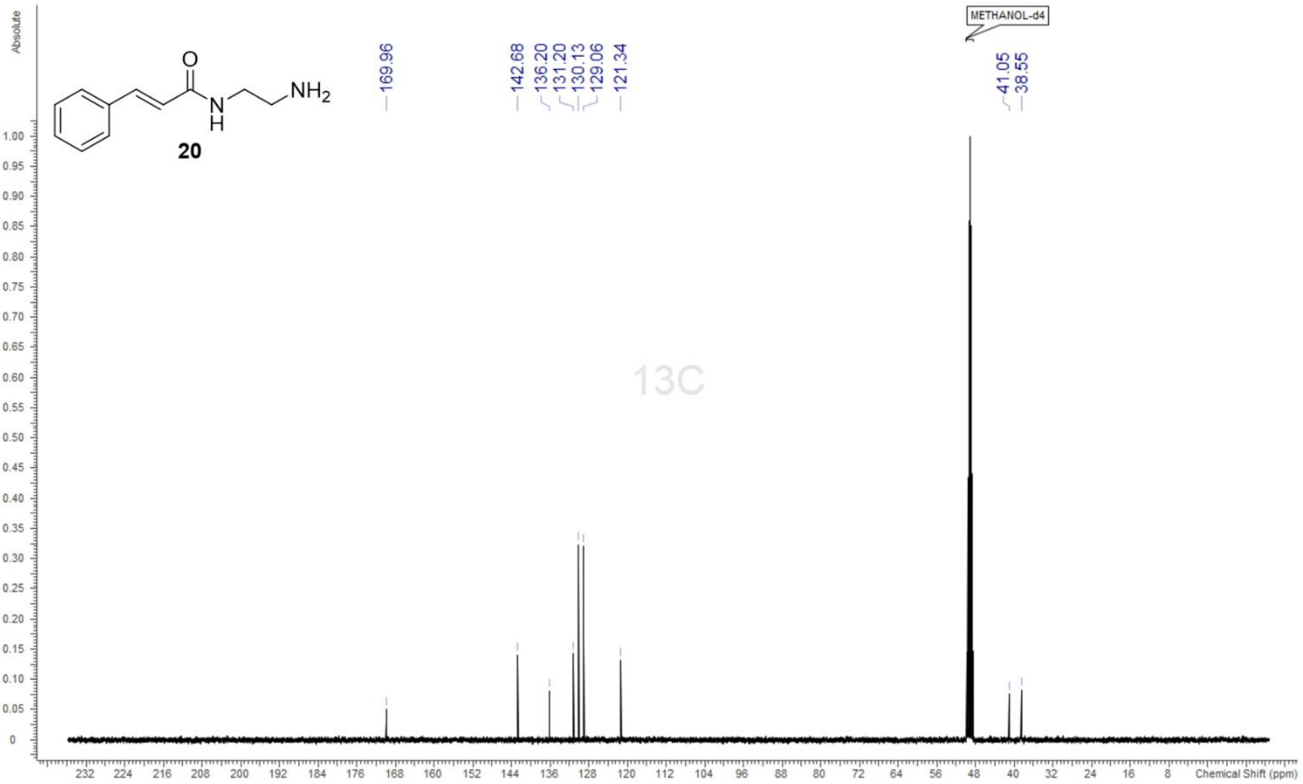

Figure S82 ^{13}C NMR (100 MHz, CD_3OD) of **20**.

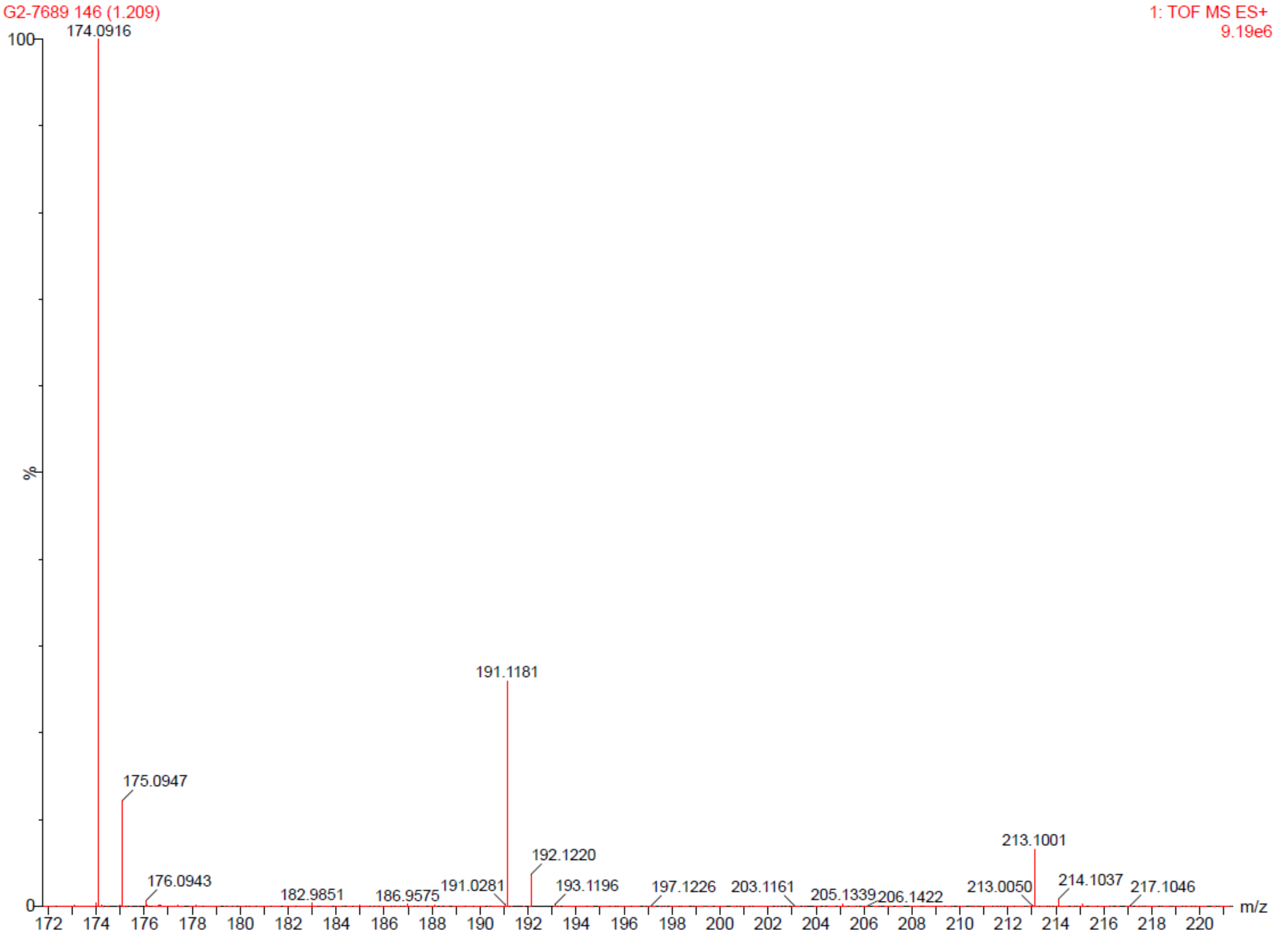

Figure S83. HMRS of **20**.

5.21. Compound 21: *N*-(2-aminoethyl)propionamide

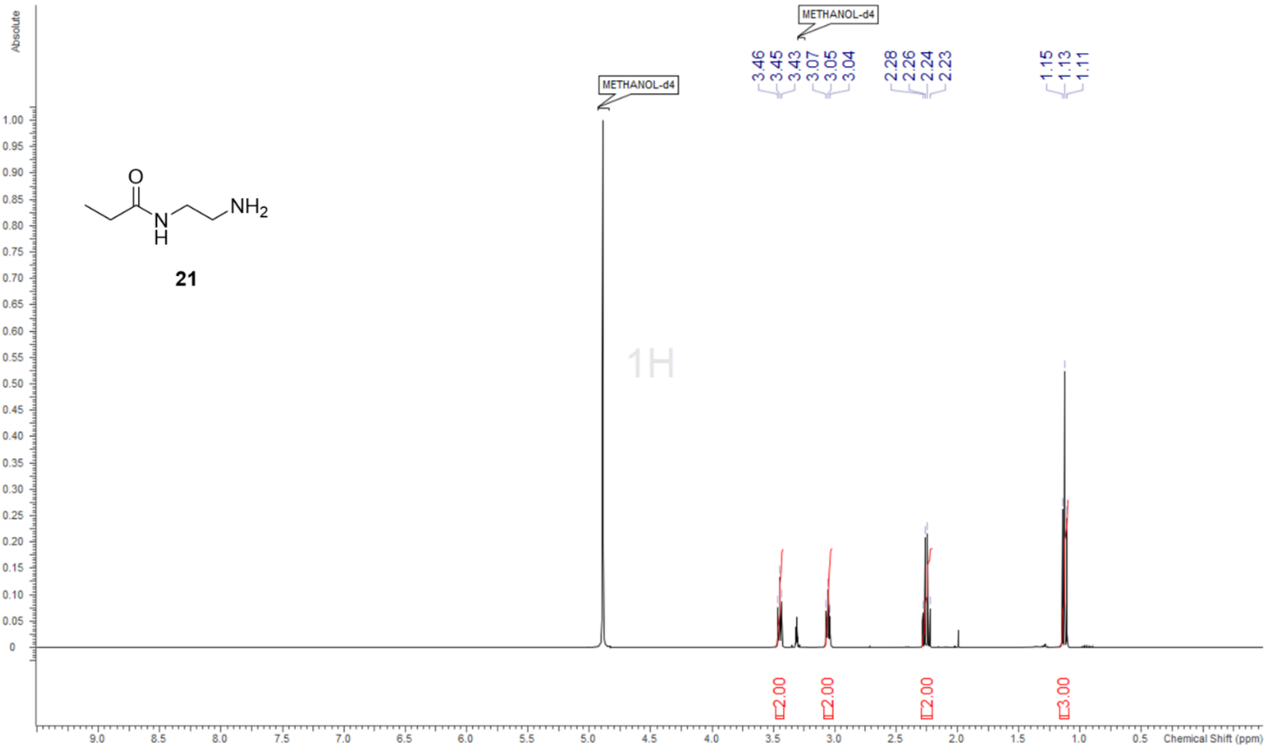

Figure S84. ^{1}H NMR (400 MHz, CD_3OD) of **21**.

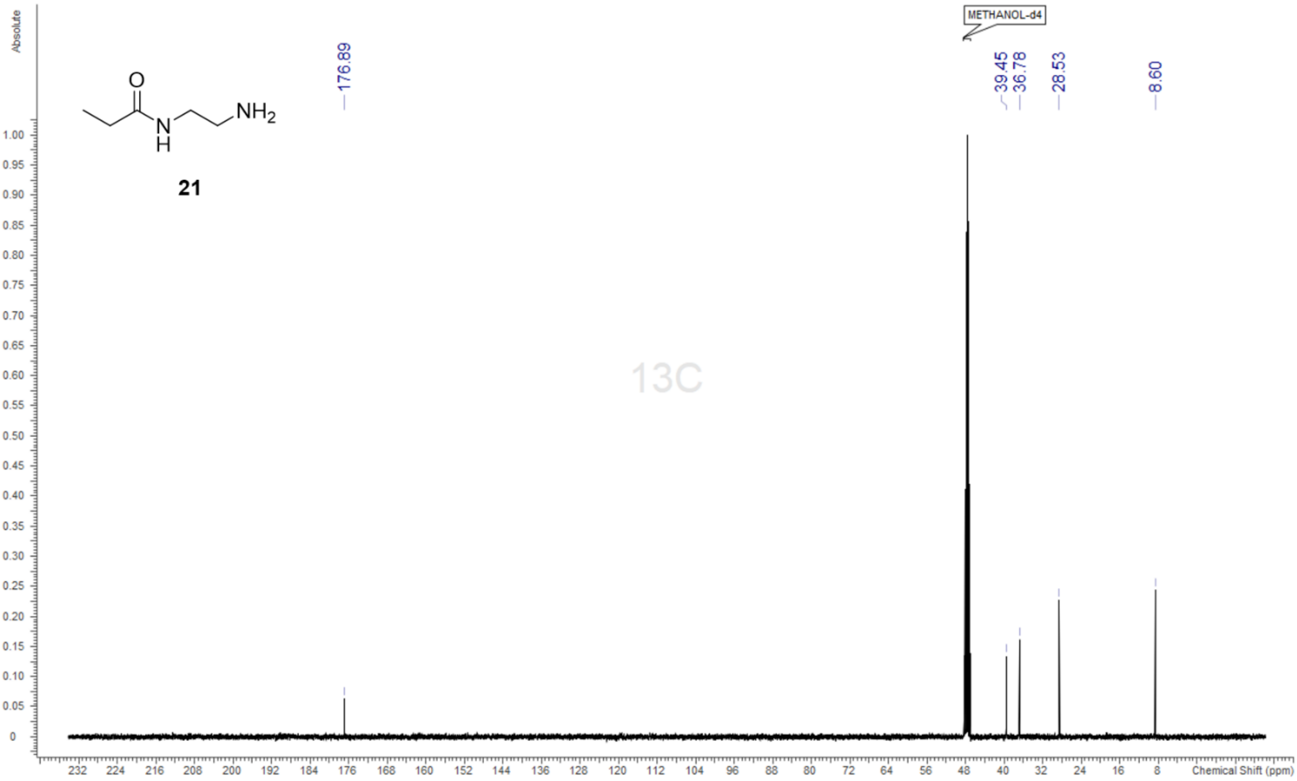

Figure S85. ^{13}C NMR (100 MHz, CD_3OD) of **21**.

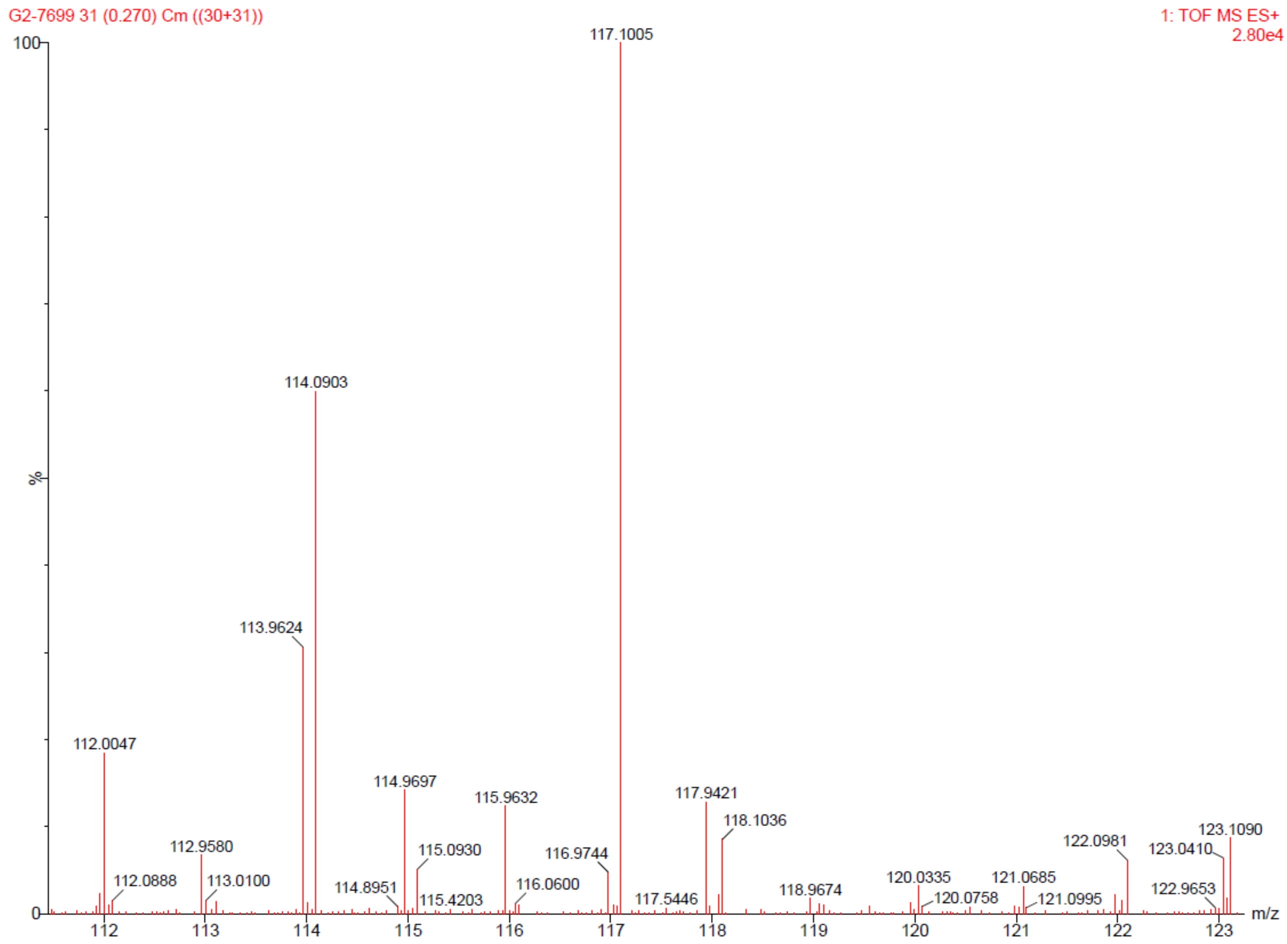

Figure S86. HMRS of **21**.

6. References

(1) Noike, M.; Ono, Y.; Araki, Y.; Tanio, R.; Higuchi, Y.; Nitta, H.; Hamano, Y.; Toyomasu, T.; Sassa, T.; Kato, N.; Dairi, T. Molecular Breeding of a Fungus Producing a Precursor Diterpene Suitable for Semi-Synthesis by Dissection of the Biosynthetic Machinery. *PLoS One* **2012**, *7* (8), e42090.

(2) Anthis, N. J.; Clore, G. M. Sequence-specific Determination of Protein and Peptide Concentrations by Absorbance at 205 Nm. *Protein Science* **2013**, *22* (6), 851–858. https://doi.org/10.1002/pro.2253.

(3) AAT Bioquest, Inc. *Quest Database™ Extinction Coefficient [TAMRA (Carboxytetramethylrhodamine)]*. https://www.aatbio.com/resources/extinction-coefficient/tamra_carboxytetramethylrhodamine.

(4) AAT Bioquest, Inc. *Quest Database™ Extinction Coefficient [Cy5 (Cyanine-5)]*. https://www.aatbio.com/resources/extinction-coefficient/cy5_cyanine_5.
